# Barbier-type *anti*-Diastereo- and Enantioselective Synthesis of β-Trimethylsilyl, Fluorinated Methyl, Phenylthio Homoallylic Alcohols

**DOI:** 10.1038/s41598-017-04986-x

**Published:** 2017-07-07

**Authors:** Rui Guo, Qin Yang, Qinshan Tian, Guozhu Zhang

**Affiliations:** State Key Laboratory of Organometallic Chemistry, Shanghai Institute of Organic Chemistry, University of Chinese Academy of Sciences, Chinese Academy of Sciences, 345 Lingling Road, Shanghai, 200032 P. R. China

## Abstract

Catalytic Asymmetric allylation of aldehydes with functionalized allylic reagents represents an important process in synthetic organic chemistry because the resulting chiral homoallylic alcohols are valuable building blocks in diverse research fields. Despite the obvious advantages of allyl halides as allylation reagent under Barbier-type conditions, catalytic asymmetric version using functionalized allyl halides remains largely underdeveloped. Here, we addressed this issue by employing a chromium-catalysis system. The use of readily available allyl bromides with γ substitutions including trimethylsilyl, fluorinated methyl and phenylthio groups provided an efficient and convenient method to introduce those privileged functionalities into homoallylic alcohols. Good yields, high anti-diastereo- and excellent enantioselectivities were achieved under mild reaction conditions.

## Introduction

**Figure 1:**
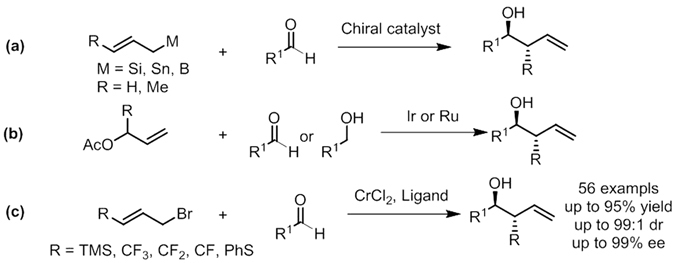
(**a**) Allylation with Silicon, Tin and Boron reagents; (**b**) Transfer hydrogenation allylation; (**c**) This work: asymmetric chromium-catalyzed allylation with functionalized allyl bromides.

Carbonyl allylation with functionalized allyl reagents has been a sustained research topic, as in this transformation, two new functionalities including one alcohol and a terminal C-C double bond are generated^1^. Thanks to the efforts from many organic chemists, traditional organometallic allylation based on Boron, Silicon and Tin reagents has become a valuable tool to generate C-C bond in a reliable and predictable way (Fig. 1a)^2–7^. Using alcohol as the equal efficient aldehyde surrogate, catalytic transfer hydrogenation protocols have considerably matured in the past decade (Fig. 1b)^8–10^, nonetheless, above mentioned methods possess several minor flaws, such as requiring the pre-generation of organometallic reagents or the usage of precious metals. At the same time, the allylation of carbonyl compounds with allylic halides using metals, typically Mg, In, as mediating reagents under Barbier-type reaction conditions is of great interest^11^. This strategy offer practical advantages as the nucleophile is generated *in situ* in the presence of electrophiles, not only circumvents the need of isolating allyl metal species, but also enables an intramolecular version.

Chromium mediated Grignard-type addition of carbohalides to aldehyde, the Nozaki-Hiyama-Kishi reaction proved to be one of the most powerful synthetic methods for carbon-carbon bond formation^12–18^, its synthetic utilities have been demonstrated in numerous complex natural products total synthesis. Despite the rapid evolution of chiral chromium ligands^19^, there remains considerable unmet challenges in enantioselective catalysis due to the generally high reactivity of organometallic allylation reagent derived from allyl halides e.g. Grignard reagents. On the other hand, asymmetric chromium catalysis holds great synthetic potential considering its extraordinary affinity towards aldehydes and mild reductive nature, together with the fruitful and ready accessible functionalized allyl halides. In line with our interests in the asymmetric carbonyl allylation with chromium complex, we recently reported enantioselective chromium catalyzed carbonyl 2-(alkoxycarbonyl)allylation leading to synthetic useful α-exo-methylene-γ-butyrolactones^20^, dearomative coupling of halomethylheteroarenes^20, 21^, chiral quaternary stereogenic centers formation^22^. Herein, we would like to report our preliminary results on incorporating synthetically useful and medicinally relevant functionalities including trimethyl silyl, fluorinated methyl and phenylthio groups into homoallylic alcohol (Fig. 1c). The resulting products are not only valuable substances themselves, but also serve as significant building blocks for further derivatizations. The use of readily available γ-functionalized allyl halides, cheap metals and chiral ligands, mild reaction conditions together with easy execution make an attractive approach which would streamline the access to a large variety of related reaction patterns.

## Results and Discussion

### *anti*-Diastereo- and Enantioselective Carbonyl (Trimethylsilyl)allylation

Organosilicon represents a privileged functionality in synthetic organic chemistry^23–25^. A variety of named reactions and useful transformations derive from the unique properties of silicon; representative examples including Peterson olefination, Brook rearrangement, Fleming-Tamao oxidation, Prins cyclization and Sakurai allylation. Thus, sustained efforts have been dedicated toward the development of efficient methods for the expedient introduction of silicon into organic molecules. Among various organosilicon compounds, allylsilane is a very important building block leading to diverse useful products including homoallylic alcohols. Chiral β-hydroxy allylsilanes and derivatives have been extensively used by Roush, Panek and others for the synthesis of 1,2- and 1,4-diols in the total synthesis of natural products^26–32^. In 2010, Krische reported an iridium catalyzed silylallylation using SEGPHOS as a chiral ligand for the synthesis of α-silyl homoallylic alcohols^33^. More recently, Barrio and Akiyama reported the chiral BrØnsted acid catalyzed carbonyl allylboration with γ-silylboronates^34, 35^. Given the broad synthetic utilities of α-silyl homoallylic alcohols, alternative methods for efficient and enantioselective synthesis of this important moiety are highly desired.

**Figure 2:**
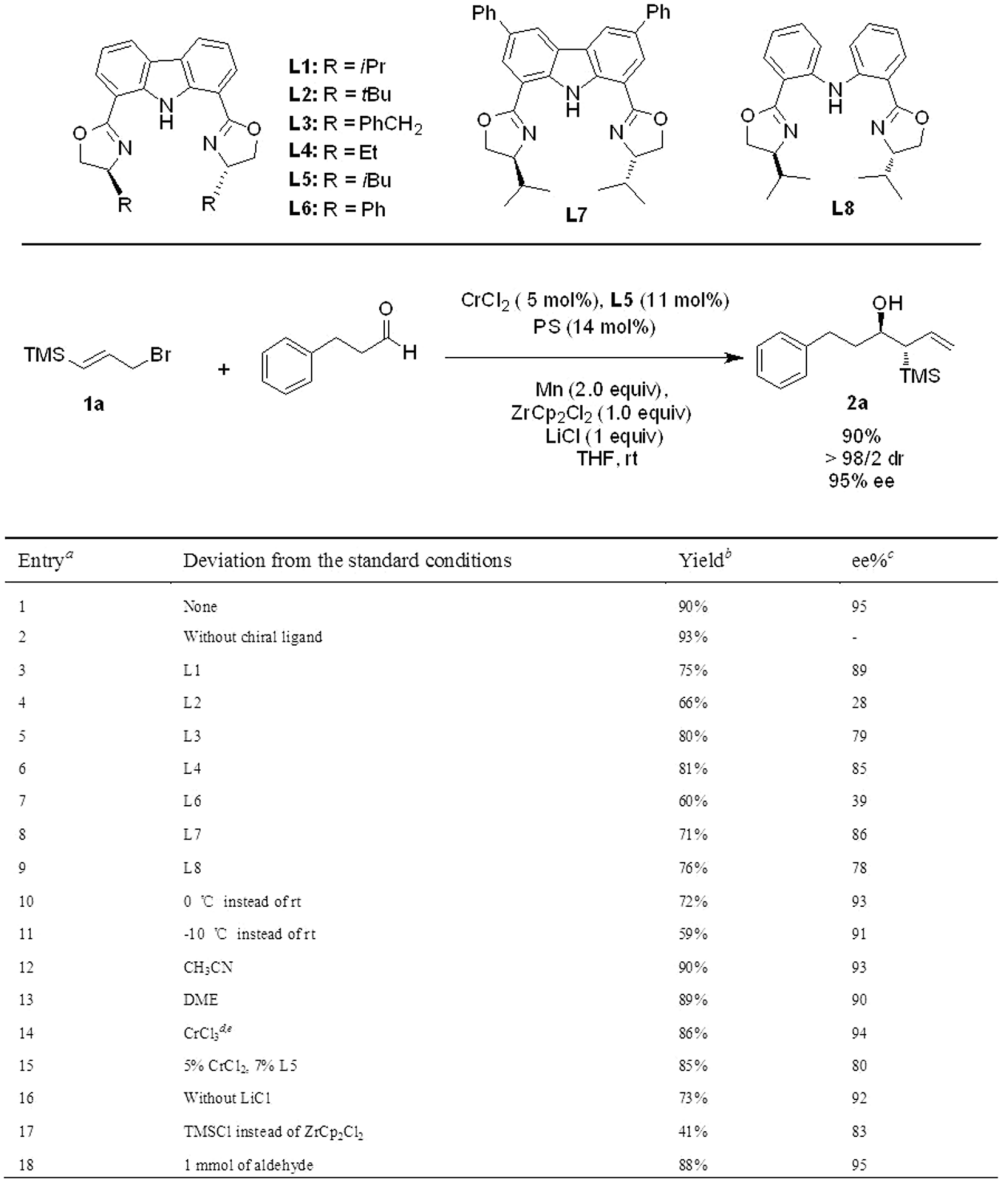
Evaluation of chiral ligands and other reaction parameters. ^a^The reactions were carried out at 0.2 mmol scale unless noted otherwise; ^b^Isolated yield; ^c^Determined by chiral HPLC analysis, the absolute configuration was determined by comparison with reported example; ^d^1 equiv of Mn was added for the complex formation; ^e^Complexation took 8 hrs.

At the outset of our study, the coupling of 3-phenylpropanal with easily accessible [(1E)-3-bromoprop-1-enyl]trimethylsilane **(1a)** was chosen as the model reaction (Fig. 2). With proton sponge as the base in the complexation step, ZrCp_2_Cl_2_ as the dissociation agent and Mn as the reducing reagent for chromium turnover. We first tested the reaction in the absence of any chiral ligands, to our delight, the desired homoallylic alcohol was generated in good yield as a single diastereomer (Fig. 2, entry 2).

We turned our attention to the development of its asymmetric variant by employing carbazole-based bisoxazoline (Nakada catalyst) as the chiral ligands^20, 36^. **L1** (R = ^i^Pr) was first examined; after quite a few trials by varying the solvents and additives based on our previous studies, product **2a** could be isolated in 75% with 89% ee (Fig. 2, entry 3). Further ligand screening revealed that an introduction of a bulkier substitution proved deleterious for the enantioselectivity, as **L2** (R = ^*t*^Bu) resulted in **2a** in 66% with only 28% ee (Fig. 2, entry 4). A comparable enantioselectivity was obtained as **L3** (R = PhCH_2_) (Fig. 2, entry 5) and **L4** (R = Et) (Fig. 2, entry 6) were employed as ligands. Finally, **L5** (R = ^*i*^Bu) gave rise to the **2a** in 90% yield with 95% ee (Fig. 2, entry 1).

All other deviations from the optimal conditions led to a decrease of the enantioselectivity and in some case even the yield. Lowering the reaction temperature didn’t benefit the overall efficiency (Fig. 2, entry 10). Solvents screening revealed that THF gave the best result, the reactions running in either CH_3_CN or DME gave **2a** in slightly lower yields and ee (Fig. 2, entry 12 and 13). Cheaper and easy handling CrCl_3_ could also be directly used, a comparable result (87% yield, 94% ee, Fig. 2, entry 14) was obtained; in this experiment, the complexation step required the addition of one equivalent of Mn metal.Entry^*a*^

It was found that ligand loadings could be lowered to 7 mol% with slight erosion of enantioselectivity (Fig. 2, entry 15). LiCl exhibited an enhancing effect on the coupling rate and enantioselectivity, which is likely to facilitate the formation of allyl species and its transmetallation to the chiral chromium complex (Fig. 2, entry 16). Both TMSCl^37–39^ and ZrCp_2_Cl_2_^40^ have been previously used as dissociating reagents, and they have various impacts on the reaction. However, in this case, TMSCl had slightly less efficiency (Fig. 2, entry 17). Notably, the reaction scale could be increased to 1 mmol with maintenance of the efficiency (Fig. 2, entry 18).

**Figure 3:**
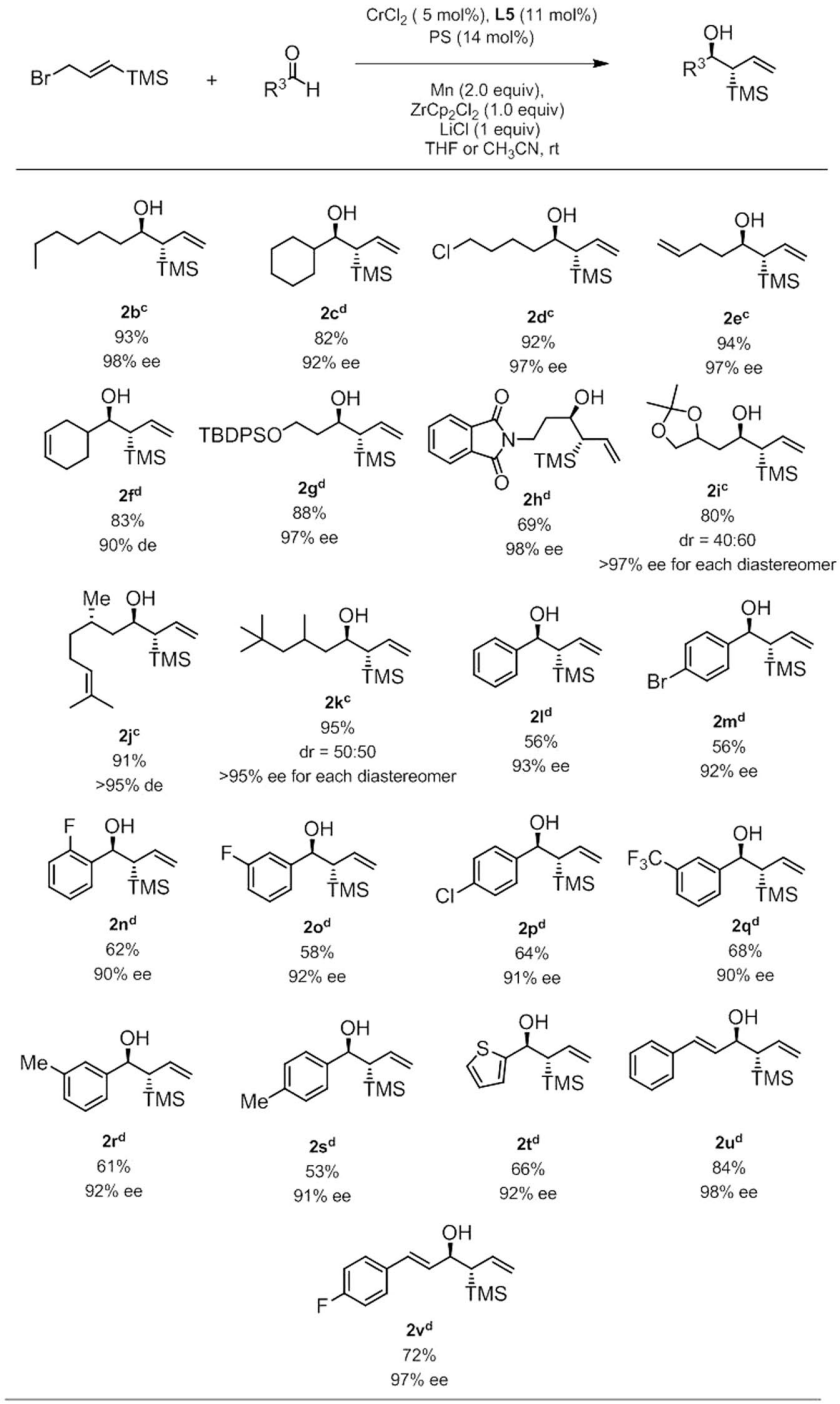
Substrate scope studies. ^a^All reactions carried out at 0.2 mmol scale under the standard conditions, ligand **L5** was used unless otherwise noted, generally over 50/1 *anti*-diastereoselectivity was observed; ^b^The absolute configurations of **2e**, **2l**, **2m**, **2p**, and **2r** were assigned by comparison with reported examples, others were by analogy; ^c^THF as solvent; ^d^CH_3_CN as solvent.

With these optimized conditions in hand (Fig. 2, entry 1), the generality of this transformation was established using a broad range of aldehydes shown in Fig. 3. Excellent *anti* - selectivity ( > 98:2) was observed for all the

cases and generally high enantioselectivity (90–98% ee) was obtained. Coupling of **1a** with representative aliphatic aldehydes including cyclohexyl carboxyaldehyde, heptaldehyde proceeded smoothly; the corresponding homoallylic alcohols **2b**, **2c** were isolated in high yields (93% and 82%) with excellent enantiomeric excess (98% and 92% ee). Substrate with a chloro group reacted well, giving **2d** in 92% yield with 97% ee. Aldehydes bearing a terminal C-C double bond or a cyclohexene moiety both participated in the (trimethylsilyl)allylation efficiently, furnishing the corresponding products **2e** and **2f** in good yields (94% and 90%) with excellent enantiomeric excess (97% and 90%). Heteroatoms with proper protecting groups such as TBDPS for oxygen atom or phthalimide for nitrogen atom are compatible under current reaction conditions, no erosion of enantiomeric excess of the corresponding products **2g** and **2h** were detected (97% ee and 98% ee, respectively). The current reaction condition tolerates a sensitive ketal moiety, protected triols **2i** was obtained in moderate yield (80%) and good ee (>97%). A naturally occurring aldehyde (−)-citronellal bearing a chiral methyl group β to the carbonyl group exhibited good reactivity and selectivity profile, the corresponding **2j** was obtained in 91% yield with 95% de. Reaction of racemic 3,5,5-trimethylhexanal provided **2k** in 95% yield with 95% ee for each diastereomer.

Attempts to expand this protocol to aryl aldehydes turned out to be successful. Benzaldehyde reacted well, giving **2l** in moderate 56% yield with 93% ee. The effect of benzene substituents was examined next. The phenyl group could be freely halogenated with Cl, Br and F without compromise of the reaction efficiency in terms of yields and ee, as products **2m** to **2p** were isolated in moderate to good yields (56%-64%) with decent ee (ranging from 91% to 94%). Electron-withdrawing CF_3_ group and weak electron-donating Me group can both be introduced into the system, leading to products **2q** to **2s** in useful level of yields (>50%) and excellent ees (>90%). Moreover, heterocycle such as thiophene was compatible under current conditions, giving the **2t** in 66% yield with 92% ee. To our delight, another important class of aldehydes, α, β-unsaturated aldehydes are suitable substrates for this chemistry as well, it was found that even higher enantiomeric excesses 97% ee for **2u** and 98% ee for **2v** were obtained from cinnamaldehyde and 4-fluorocinnamaldehyde.

Notably, for several aliphatic aldehydes (**2c**, **2f**, **2g**, **2h**), most aromatic and α, β-unsaturated aldehydes, CH_3_CN was a better solvent than THF in terms of bigger ee value, generally over 20% difference was observed.

### *anti*-Diastereo- and Enantioselective Carbonyl (fluoronated methyl) allylation

**Figure 4:**
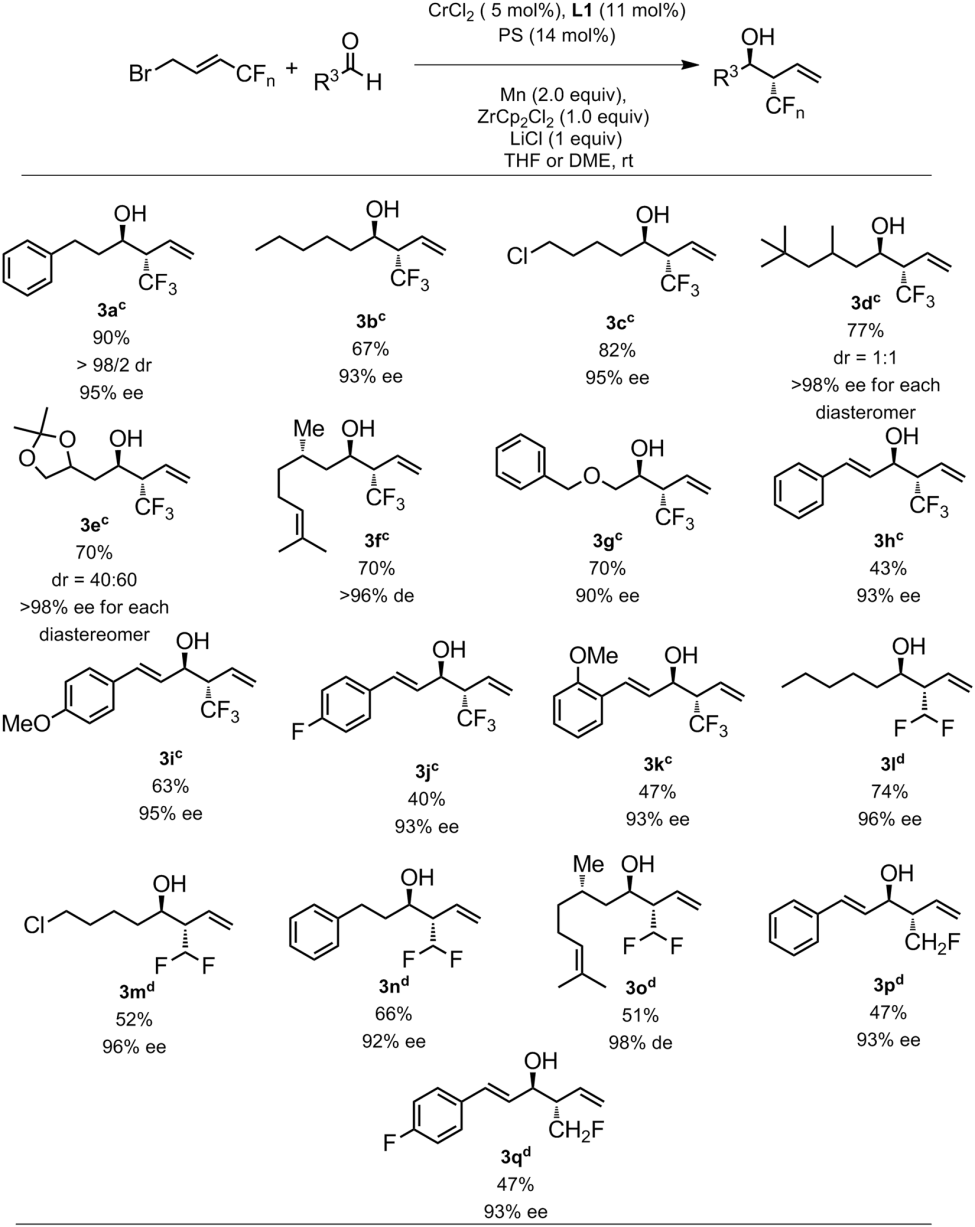
Substrate scope studies. ^a,b a^All reactions carried out at 0.2 mmol scale under the standard conditions, ligand **L1** was used unless otherwise noted, generally over 50/1 *anti*-diastereoselectivity was observed; ^b^The absolute configurations of **3a**, **3g**, and **3h** were assigned by comparison with reported examples and X-Ray single crystal analysis of **6**, others were by analogy; ^c^THF as solvent; ^d^DME as solvent.

The introduction of fluorine atoms into organic molecules often leads to dramatic changes in their properties such as solubility, metabolic stability, and bioavailability^41^. Additionally, fluoroalkyl groups, especially the trifluoromethyl, difluoromethyl groups are strongly electron-withdrawing and highly hydrophobic. Because of these desirable properties, fluoroalkylated compounds are widely used in materials science, argochemistry and medicinal chemistry^42, 43^. Crotylation of carbonyl compounds constitutes one significant transformation in synthetic organic chemistry, as the resulting β-methyl homoallylic alcohols serve as indispensable segment in numerous polyketide natural products and advanced intermediates leading to molecules with bio- or medicinal significance. Thus, enantioselective introduction of fluorinated methyl group into the homoallylic system have been an important subject. However, to the best of our knowledge, only a few methods have been reported^44–46^. In 2010, Krische reported an iridium catalyzed (trifluromethyl) allylation using SEGPHOS as a chiral ligand under the transfer hydrogenation conditions^47^. Despite the above mentioned elegant strategies for introduction of trifluoromethyl group, the demand for alternative efficient methods, and the lack of practical protocol for introducing difluoromethyl and monofluoromethyl groups prompted us to explore chromium-catalyzed asymmetric carbonyl allylation with (fluorinated methyl) allyl halides. γ-trifluoromethylallyl halides are simple and abundant chemicals and easy to prepare. Directly use of them as carbonyl allylation reagents to generate α-trifluoromethyl homoallylic alcohols in racemic manner have been investigated in indium catalysis^48–51^. However, neither asymmetric version nor (difluoro- or monofluoromethyl) allylation has been reported. We began with investigating the cross-coupling between γ-trifluoromethylallyl bromide and dihydro cinnamaldehyde. After examination of a considerable variety of reaction parameters, we were pleased to find the anticipated trifluomethylated homoallylic alcohol could be obtained in 90% isolated yield with 95% ee as a single diastereomer. Ligands screening revealed that **L1** was the optimal ligand (Figs 3a and 4).

This highly enantioselective synthesis of **3a** can also be expanded to reactions with a variety of aldehydes, and high enantioselectivity (93–98% ee) was obtained (Fig. 4). Heptaldehyde participated in this reaction efficiently; the corresponding **3b** was isolated in moderate yield with excellent enantiomeric excess (93% ee). A terminal chloro group was tolerated under current reaction condition; desired **3c** was obtained in 82% yield with 95% ee. Reaction of (−)-citronellal bearing a chiral methyl group β to the carbonyl group proceeded well to provide **3d** in 70% yield with 96% ee. Substrate with a sensitive ketal moiety was also amenable to the (trifluoro methyl)allylation, giving good overall yield with excellent ee for both diastereoisomers. A range of α, β-unsaturated aldehydes including unsubstitued, *para*-methoxy, *ortho*-methoxy and *para*-fluoro cinnaldehyde reacted well to provide the products (**3h**–**3k**) in useful level of yields with excellent ee. However, our attempts to apply the aryl aldehydes to the coupling reaction failed. After assay the full scope of (trifluoromethyl) allylation, we turned our attention to the unprecedented (difluoromethyl) allylation. To our delight, the couplings between γ-difluoromethylallyl bromide with four aliphatic aldehydes including dihydro cinnamaldehyde, hexanal, 5-chloropetanal and (−)-citronellal proceeded well to afford the desired difluoromethylated homoallylic alcohols in moderate to good yields with excellent enantiomeric excess (92–98% ee). However, further scope studies revealed that aryl and α, β-unsaturated aldehydes are not good substrates under current reaction conditions. We then tested the challenging (monofluoromethyl) allylation due to the potential competing (monobromomethyl) allylation. As anticipated, the reactions proceeded sluggishly with most of the aldehydes tested. Nevertheless, we are pleased to find that two α, β-unsaturated aldehydes are suitable substrates under current reaction conditions. The corresponding **3p** and **3q** were obtained in moderate yields in excellent ee (93% each).

Notably, for (difluoro- or monofluoromethyl) allylation, DME was a better solvent than THF in terms of bigger ee value, generally over 10% difference was observed.

### *anti*-Diastereo- and Enantioselective Carbonyl (phenylthio) allylation

Sulfur-derived functional groups are ubiquitous in synthetic organic chemistry, pharmaceutical industry, material science and food chemistry, which were evidenced by the fact that over 326 FDA approved drugs containing sulfur functionalities^52–58^. Among them, thioether is especially popular and can be found in a broad range of pharmaceuticals and natural products^59, 60^. Although there is a vast array of methods have been developed to incorporating a sulfur into a specific position in a molecule, the catalytic asymmetric construction of a sulfide-bearing carbon centers is still rare^61, 62^. Thus, the development of an efficient and convenient synthetic method, using readily available building blocks would be of meaningful importance in both the synthetic organic chemistry and pharmaceuticals advancement.

**Figure 5:**
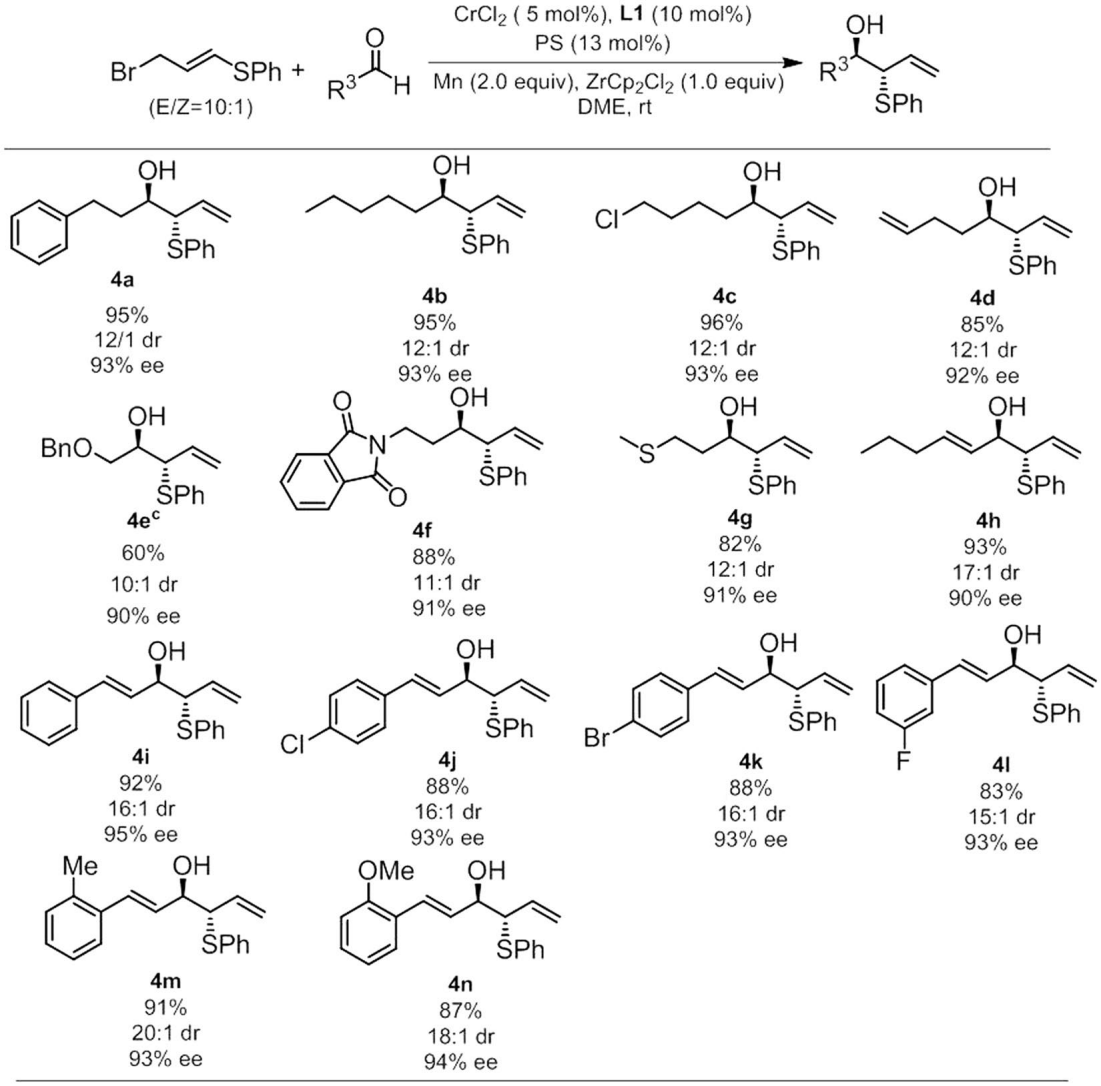
Substrate scope studies. ^a^All reactions carried out at 0.1 mmol scale under the standard conditions, ligand **L1**was used unless otherwise noted; ^b^The absolute configurations of **4e** were assigned by comparison with reported examples, others were by analogy. ^c^at 0 °C for 24 h.

In our attempt to introduce a phenylthio unit into the homoallylic system, model reaction between (*E*)-(3-bromoprop-1-en-1-yl)(phenyl) sulfane and 3-phenylpropanal was selected to perform in the presence of **L1**-CrCl_2_ complex under previously established conditions. To our delight, the desired α-benzylthio homoallylic alcohol was isolated in moderate yield with good dr and excellent ee. After a few reaction optimization trials, the product **4a** was obtained in 95% isolated yield with 12:1 dr and 93% ee (Figs 4a and 5). Notably, the diastereoselectivity dropped to 2:1 in the absence of chiral ligand. Having obtained the optimized reaction conditions, the issues with respect to the functional group tolerance were thus addressed, and the results are summarized in the Fig. 5. Reaction of linear hexanal gave similar results in terms of yield and selectivity (**4b**). Substrates with synthetically useful functional groups such as Cl and terminal double bond (**4c** and **4d**) participated in this reaction efficiently, to deliver the products in decent yield with good dr and excellent ee. We were pleased to find that heteroatoms including O, N, S with proper protecting groups are well tolerated in this reaction, which offers the opportunity for further synthetic elaborations. Beside aliphatic aldehydes, α, β-unsaturated aldehydes are amenable substrates under current reaction conditions. An equal level of yield and enantioselectivity with even higher diastereoselectivity was observed from the reaction of (*E*)-hex-2-enal. An aryl conjugated enal proved to be beneficial, an improved diastereoselectivity and enantioselectivity were achieved for Cinnamaldehyde. Furthermore, substrates bearing substituents such as *para*-Cl, *para*-Br, *meta*-F, *ortho*-MeO and *ortho*-Me on the aryl ring of cinnamaldehyde also were engaged well in this reaction to furnish the desired products in good yields with synthetically useful level of diastereoselectivity and excellent enantioselecitivity.

**Figure 6:**
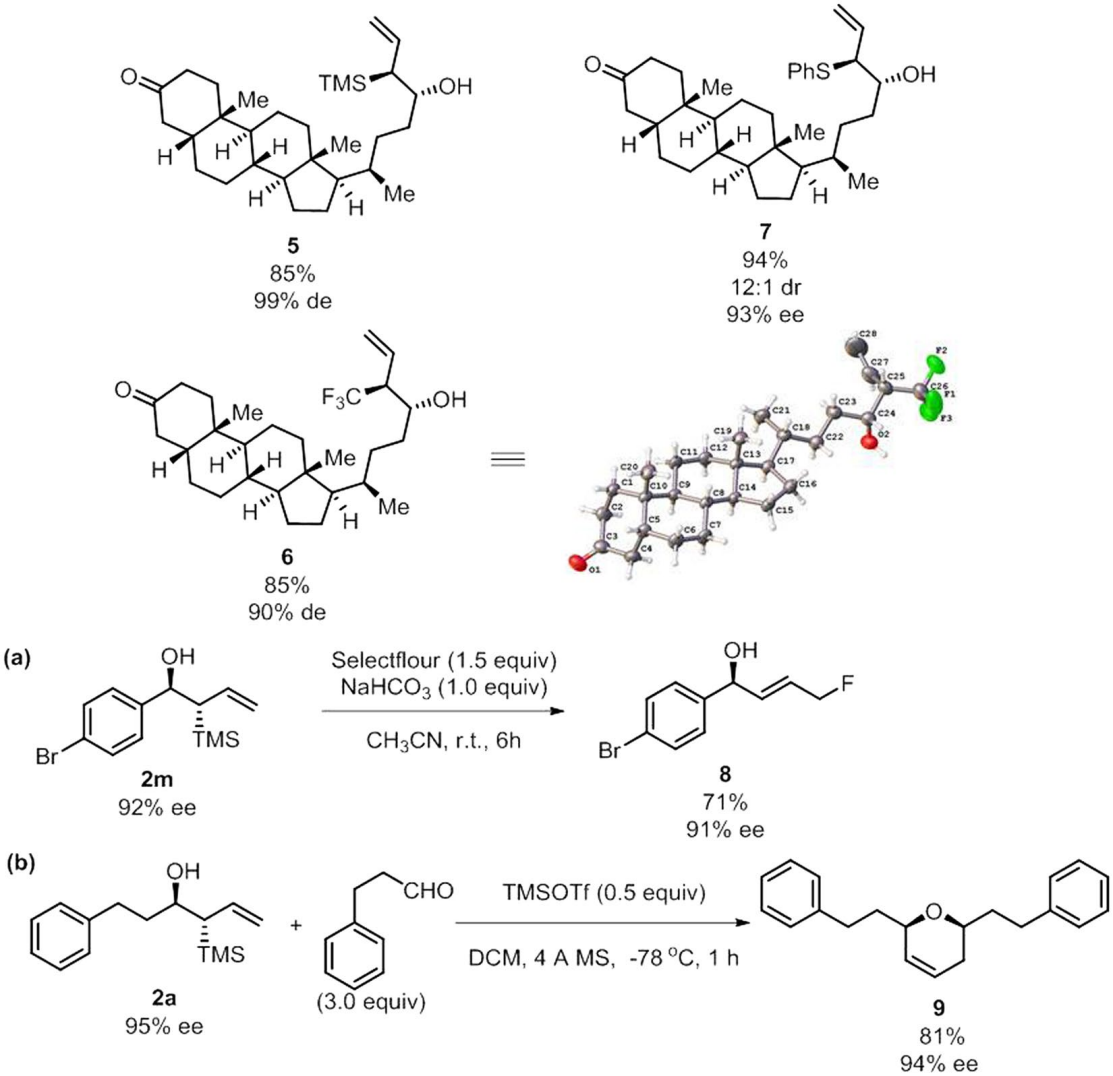
Synthetic utilities.

To demonstrate the potential of this protocol in the synthesis of relatively complex molecules, an aldehyde derived from natural occurring lithocholic acid was subjected to the reactions with three allyl bromides, the desired homoallylic alcohols were obtained in good yields with excellent de. The synthetic utility of the resulting homoallylic alcohols were further illustrated in two short transformations of allylsilane (Fig. 6). Following a reported procedure, treatment of **2m** with Selectfluor under buffered reaction conditions afforded product **8** in good yield, with almost complete preservation of the optical purity^34^. Finally, **2a** underwent Prins cyclization with dihydrocinnamaldehyde in the presence of TMSOTf to furnish the synthetically useful dihydropyran **9** in good yield and excellent diastereoselectivity with high optical purity.

## Conclusions

In summary, an efficient synthesis of chiral β-functionalized homoallylic alcohols has been achieved through chromium catalyzed asymmetric allylation of aldehydes with three types of γ-substituted allyl bromides. This protocol features readily available allylation reagents, convenient operation and mild reaction condition, broad functional group tolerance and high levels of diastereoselectivity and excellent enantioselectivity. The synthetic value of this methodology was demonstrated in two short transformations. We positively believe this method will find applications in the enantioselective synthesis of related pharmaceutical compounds and biomolecules.

## Methods

(For details of the synthetic procedures, see Supplementary Methods pages 3–7).

General Procedure: To a mixture of anhydrous chromium(II) chloride (1.2 mg, 0.01 mmol, 5.0 mmol%), 1,8-bis((S)-4-((R)-sec-butyl)-4,5-dihydrooxazol-2-yl)-9H-carbazole (**L-5**, 9.0 mg, 0.022 mmol, 10.8 mmol%) and Proton sponge (6.0 mg, 0.028 mmol, 14.0 mmol%) was added THF (1.0 ml) under an nitrogen atmosphere. The mixture was stirred vigorously at room temperature for 3 hours before it was transferred into a vessel charged with Zr(Cp)_2_Cl_2_ (60.0 mg, 0.2 mmol, 1.0 eq.), LiCl (8.4 mg, 0.2 mmol, 1.0 eq.) and Manganese powder (22.0 mg, 0.4 mmol, 2.0 eq.). Then (E)-(3-bromoprop-1-en-1-yl)trimethylsilane (77 mg, 0.4 mmol, 2.0 eq.) and aldehyde (0.2 mmol, 1.0 eq.) were added in succession. The resulting suspension was left stirred at room temperature overnight. After the full consumption of aldehyde, the reaction mixture was diluted with undried EA and the resulting suspension was filtered over a pad of silica gel using EA as eluent. Volatiles were evaporated in vacuo. The residue was purified by chromatography to afford the product.

### (3R,4S)-1-phenyl-4-(trimethylsilyl)hex-5-en-3-ol (2a)

^1^H NMR (400 MHz, CDCl_3_): δ 7.25–7.17 (m, 2H), 7.16–7.05 (m, 3H), 5.73 (dt, *J* = 17.1, 10.4 Hz, 1H), 4.98 (dd, *J* = 10.4, 1.6 Hz,1H), 4.88 (d, *J* = 17.1 Hz,1H), 3.75 (dd, *J* = 12.7, 5.2 Hz, 1H), 2.72–2.53 (m, 2H), 1.79–1.68 (m, 2H), 1.65 (dd, *J* = 10.6, 5.4 Hz, 1H), 1.54 (br, 1H), −0.02 (s, 9H); ^13^C NMR (101 MHz, CDCl_3_):δ 142.1, 135.7, 128.4, 128.3, 125.7, 115.2, 71.0, 42.6, 39.0, 32.3, −2.0; IR (neat) cm^−1^ $\tilde{{\rm{v}}}$: 3456,3027,1247,899,839,749, 698;HRMS (EI(+), 70 eV): C_15_H_24_OSi [M-H]^+^: calcd. 247.1596, found.247.1513; [α]_D_^20^ = +23.2 (c = 1.00, CH_2_Cl_2_); HPLC (Chiralcel OD-H column, hexanes:i-PrOH = 95:5, 0.5 mL/min, 210 nm), t_minor_ = 9.8 min, t_major_ = 12.1 min, 95% ee.

### (3S,4R)-3-(trimethylsilyl)dec-1-en-4-ol (2b)

^1^H NMR (400 MHz, CDCl_3_): δ 5.79 (dt, *J* = 17.1, 10.4 Hz, 1H), 5.03 (dd, *J* = 10.2, 2.1 Hz, 1H), 4.92 (dd, *J* = 17.1, 2.0 Hz, 1H), 3.79 (dt, *J* = 7.5, 5.1 Hz, 1H), 1.67 (dd, *J* = 10.6, 5.3 Hz, 1H), 1.52–1.36 (m, 3H), 1.35–1.20 (m, 8H), 0.88 (t, *J* = 6.7 Hz, 3H), 0.04 (s, 9H); ^13^C NMR (101 MHz, CDCl_3_):δ 135.9, 115.0, 71.6, 42.6, 37.3, 31.8, 29.3, 25.8, 22.6, 14.1, −2.0. IR (neat) cm^−1^ $\tilde{{\rm{v}}}$: 3696, 2962,2856, 1261, 1093, 1021, 800, 690; HRMS (EI(+), 70 eV): C_13_H_28_OSi [M-H]^+^: calcd. 227.1909, found 227.1826; [α]_D_^20^ = +2.8 (c = 2.00, CH_2_Cl_2_); Enantiomeric excess was determined by HPLC analysis of the 3,5-nitrobenzoate derivative of the product (Chiralcel OD-H column, hexanes:i-PrOH = 98:2, 0.4 mL/min, 210 nm), t_major_ = 13.9 min, t_minor_ = 15.3 min, 96% ee.

### (1R,2S)-1-cyclohexyl-2-(trimethylsilyl)but-3-en-1-ol (2c)

^1^H NMR (400 MHz, CDCl_3_): δ 5.82 (dt, *J* = 17.1, 10.5 Hz, 1H), 5.00 (dd, *J* = 10.2, 2.2 Hz, 1H), 4.89 (dd, *J* = 17.1, 2.0 Hz, 1H), 3.43 (dd, *J* = 7.2, 4.5 Hz, 1H),1.91–1.82 (m, 2H), 1.79–1.58 (m, 4H), 1.42–1.32 (m, 1H), 1.30–1.05 (m, 4H), 0.95 (dtd, *J* = 15.8, 12.3, 2.6 Hz, 2H), 0.05 (d, *J* = 11.7 Hz, 9H); ^13^C NMR (101 MHz, CDCl_3_):δ 135.6, 114.4,76.2, 42.4, 39.3, 29.3, 28.5, 26.4, 26.2, 25.9, −2.1; IR (neat) cm^−1^ $\tilde{{\rm{v}}}$: 3492, 2926,2854, 1450, 1248, 1037, 895, 839, 693; HRMS (EI(+), 70 eV): C_13_H_26_OSi[M-OH]^+^: calcd. 209.1753, found: 209.1720;[α]_D_^20^ = −6.1 (c = 0.20, CH_2_Cl_2_); Enantiomeric excess was determined by HPLC analysis of the 3,5-nitrobenzoate derivative of the product (Chiralcel OD-H column, hexanes:i-PrOH = 99:1, 0.3 mL/min, 210 nm), t_major_ = 23.0 min, t_minor_ = 25.2 min, 92% ee.

### (3S,4R)-8-chloro-3-(trimethylsilyl)oct-1-en-4-ol (2d)

^1^H NMR (400 MHz, CDCl_3_): δ 5.78 (dt, *J* = 17.1, 10.4 Hz, 1H), 5.04 (dd, *J* = 10.2, 2.0 Hz, 1H), 4.93 (dd, *J* = 17.1, 1.8 Hz, 1H), 3.79 (d, *J* = 4.6 Hz, 1H), 3.53 (t, *J* = 6.7 Hz, 2H), 1.85–1.73 (m, 2H), 1.71–1.60 (m, 2H), 1.59–1.40 (m, 4H), 0.04 (s, 9H).^13^C NMR (101 MHz, CDCl_3_): δ 135.6, 115.3, 71.3, 45.0, 42.6, 36.4, 32.5, 23.2, −2.0. IR (neat) cm^−1^ $\tilde{{\rm{v}}}$: 3463, 2959, 1625, 1449, 1412, 1256, 1089, 1014, 838, 796, 692; HRMS (EI(+), 70 eV): C_11_H_23_ClOSi [M-H]^+^: calcd. 233.1207, found 233.1124; [α]_D_^20^ = +1.5 (c = 1.50, CH_2_Cl_2_); Enantiomeric excess was determined by HPLC analysis of the 3,5-nitrobenzoate derivative of the product (Chiralcel OD-H column, hexanes:i-PrOH = 95:5, 0.5 mL/min, 210 nm), t_major_ = 14.7 min, t_minor_ = 19.5 min, 97% ee.

### (3S,4R)-3-(trimethylsilyl)octa-1,7-dien-4-ol (2e)

^1^H NMR (400 MHz, CDCl_3_): δ 5.93–5.69 (m, 2H), 5.16–4.82 (m, 4H), 3.82 (dd, *J* = 12.6, 5.3 Hz, 1H), 2.24–2.06 (m, 2H), 1.67 (dd, *J* = 10.6, 5.2 Hz, 1H), 1.64–1.49 (m, 3H), 0.04 (s, 9H); ^13^C NMR (101 MHz, CDCl_3_):δ 138.5, 135.8, 115.1, 114.7, 71.0, 42.7, 36.3, 30.3, −2.0; IR (neat) cm^−1^ $\tilde{{\rm{v}}}$: 3359, 2922, 2852, 1734, 1658, 1279, 1253, 1087, 801, 700; HRMS (EI(+), 70 eV): C_11_H_22_OSi[M-H]^+^: calcd. 197.1440, found197.1368; [α]_D_^20^ = +5.3 (c = 0.20, CH_2_Cl_2_); Enantiomeric excess was determined by HPLC analysis of the 3,5-nitrobenzoate derivative of the product (Chiralcel OD-H column, hexanes:i-PrOH = 98:2, 0.4 mL/min, 210 nm), t_major_ = 17.0 min, t_minor_ = 20.2 min, 97% ee.

### (1R,2S)-1-(cyclohex-3-en-1-yl)-2-(trimethylsilyl)but-3-en-1-ol (2 f)

^1^H NMR (400 MHz, CDCl_3_): δ 5.84 (dt, *J* = 17.7, 10.4 Hz, 1H), 5.74–5.57 (m, 2H), 5.01 (d, *J* = 10.2 Hz, 1H), 4.91 (d, *J* = 17.1 Hz, 1H), 3.53 (d, *J* = 34.4 Hz, 1H), 2.21–1.79 (m, 5H), 1.77–1.38 (m, 4H), 0.05 (s, 9H).^13^C NMR (101 MHz, CDCl_3_):δ 135.4, 135.2, 127.0, 126.2, 126.1, 114.6, 75.6, 75.6, 39.6, 39.3, 38.6, 38.5,28.0, 27.4, 25.2, 25.1,24.5, −2.1, −2.2; IR (neat) cm^−1^ $\tilde{{\rm{v}}}$: 2963, 1261, 1091, 1020, 866, 799, 700; HRMS (EI(+), 70 eV): C_13_H_24_OSi [M-H]^+^: calcd. 223.1596, found 223.1514; [α]_D_^20^ = −1.2 (c = 0.40, CH_2_Cl_2_). Enantiomeric excess was determined by HPLC analysis of the 3,5-nitrobenzoate derivative of the product (Chiralcel OD-H column, hexanes:i-PrOH = 99.5:0.5, 0.4 mL/min, 210 nm), t_major_ = 26.8 min, 27.8 min, t_minor_ = 29.5 min, 31.3 min, 90% de.

### (3R,4S)-1-((tert-butyldiphenylsilyl)oxy)-4-(trimethylsilyl)hex-5-en-3-ol (2g)

^1^H NMR (400 MHz, CDCl_3_): δ 7.71 (dd, *J* = 7.6, 1.4 Hz, 4H), 7.50–7.38 (m, 6H), 5.93 (dt, *J* = 17.1, 10.4 Hz, 1H), 5.01 (dd, *J* = 10.3, 2.3 Hz, 1H), 4.89 (ddd, *J* = 17.1, 2.2, 0.6 Hz, 1H), 4.17 (ddd, *J* = 9.3, 4.1, 2.9 Hz, 1H), 3.92–3.78 (m, 2H), 2.75 (s, 1H), 1.90–1.78 (m, 1H), 1.64 (dd, *J* = 10.6, 4.4 Hz, 1H), 1.62–1.55 (m, 1H), 1.08 (s, 9H), 0.09 (s, 9H).^13^C NMR (101 MHz, CDCl_3_): δ 136.2, 135.6, 135.5, 133.2, 133.1, 129.8, 129.7, 127.7, 114.0, 70.9, 63.2, 43.2, 39.0, 26.8, 19.0, −2.1; IR (neat) cm^−1^ $\tilde{{\rm{v}}}$: 3522, 3071, 2955, 1624, 1469, 1426, 1390, 1248, 1081, 899, 837, 738, 702; HRMS (EI(+), 70 eV): C_25_H_38_O_2_Si_2_[M+H]^+^: calcd. 427.2410, found 427.2484; [α]_D_^20^ = +0.8 (c = 1.00, CH_2_Cl_2_); HPLC (Chiralcel OD-H column, hexanes:i-PrOH = 99:1, 0.4 mL/min, 210 nm), t_major_ = 9.5 min, t_minor_ = 10.1 min, 97% ee.

### 2-((3R,4S)−3-hydroxy-4-(trimethylsilyl)hex-5-en-1-yl)isoindoline-1,3-dione (2h)

^1^H NMR (400 MHz, CDCl_3_): δ 7.85 (dd, *J* = 5.4, 3.1 Hz, 2H), 7.72 (dd, *J* = 5.4, 3.0 Hz, 2H), 5.82 (dt, *J* = 17.1, 10.4 Hz, 1H), 5.01 (dd, *J* = 10.2, 2.0 Hz, 1H), 4.89 (dd, *J* = 17.1, 1.8 Hz, 1H), 3.91–3.72 (m, 3H), 2.64 (d, *J* = 4.5 Hz, 1H), 1.84 (ddd, *J* = 15.0, 10.2, 5.2 Hz, 1H), 1.78–1.68 (m, 1H), 1.62 (dd, *J* = 10.6, 4.0 Hz, 1H), 0.00 (d, *J* = 6.1 Hz, 9H); ^13^C NMR (101 MHz, CDCl_3_): δ 174.6, 168.9, 135.5, 134.0, 132.0, 123.3, 115.0, 68.3, 42.5, 36.1, 35.1, −2.2; IR (neat) cm^−1^ $\tilde{{\rm{v}}}$: 3503, 2957,1707, 1621,1251, 1049, 838, 794, 719; HRMS (EI(+), 70 eV): C_17_H_23_NO_3_Si[M-H]^+^: calcd. 316.1447, found 316.1364;[α]_D_^20^ = −2.9 (c = 1.00, CH_2_Cl_2_). HPLC (Chiralcel OD-H column, hexanes:i-PrOH = 98:2, 0.4 mL/min, 210 nm), t_major_ = 27.5 min, t_minor_ = 29.8 min, 98% ee.

### (2R,3S)-1-(2,2-dimethyl-1,3-dioxolan-4-yl)-3-(trimethylsilyl)pent-4-en-2-ol (2i)

Minor: ^1^H NMR (400 MHz, CDCl_3_): δ 5.92 (dt, *J* = 17.2, 10.4 Hz, 1H), 4.98 (dd, *J* = 10.2, 2.1 Hz, 1H), 4.86 (dd, *J* = 17.2, 2.2 Hz, 1H), 4.25 (ddd, *J* = 10.1, 8.4, 3.3 Hz, 1H), 4.07 (dd, *J* = 8.0, 6.0 Hz, 2H), 3.54 (t, *J* = 7.7 Hz, 1H), 3.05 (br, 1H), 1.75 (dt, *J* = 14.2, 9.8 Hz, 1H), 1.61–1.51 (m, 2H), 1.41 (s, 3H), 1.36 (s, 3H), 0.05 (s, 9H); ^13^C NMR (101 MHz, CDCl_3_): δ 135.8, 114.0, 109.4, 76.1, 71.1, 69.8, 43.3, 40.6, 26.9, 25.8, −2.2. Major: ^1^H NMR (400 MHz, CDCl_3_): δ 5.80 (dt, *J* = 17.1, 10.4 Hz, 1H), 5.04 (dd, *J* = 10.3, 2.0 Hz, 1H), 4.94 (dd, *J* = 17.1, 2.0 Hz, 1H), 4.39–4.28 (m, 1H), 4.08 (dd, *J* = 8.1, 6.1 Hz, 2H), 3.58 (t, *J* = 7.8 Hz, 1H), 2.14 (br, 1H), 1.73–1.69 (m, 1H), 1.69–1.60 (m, 2H), 1.41 (s, 3H), 1.36 (s, 3H), 0.05 (s, 9H); ^13^C NMR (101 MHz, CDCl_3_): δ 136.0, 115.3, 108.6, 73.8, 69.5, 68.3, 43.8, 40.5, 26.9, 25.6, −1.9. IR (neat) cm^−1^ $\tilde{{\rm{v}}}$: 3500, 3074, 2934,1625, 1457, 1375, 1247, 1061, 838, 693;HRMS (EI(+), 70 eV): C_13_H_26_O_3_Si[M-H]^+^: calcd. 257.1651, found 257.1569; [α]_D_^20^ = +9.3 (c = 2.60, CH_2_Cl_2_). Enantiomeric excess was determined by HPLC analysis of the 3,5-nitrobenzoate derivative of the product (Chiralcel OD-H column, hexanes:i-PrOH = 80:20, 1 mL/min, 210 nm), t_minor_ = 4.8 min, 5.9 min, t_major_ = 5.2 min, 10.7 min, >97% de.

### (3S,4R,6S)-6,10-dimethyl-3-(trimethylsilyl)undeca-1,9-dien-4-ol (2j)

^1^H NMR (400 MHz, CDCl_3_): δ 5.78 (dt, *J* = 17.1, 10.5 Hz, 1H), 5.10 (t, *J* = 7.1 Hz, 1H), 5.04 (dd, *J* = 10.3, 2.0 Hz, 1H), 4.93 (dd, *J* = 17.0, 1.5 Hz, 1H), 3.95–3.85 (m, 1H), 2.04–1.93 (m, 2H), 1.72–1.57 (m, 8H), 1.50–1.46 (m, 1H), 1.35–1.27 (m, 2H), 1.23–1.13 (m, 2H), 0.90 (d, *J* = 6.6 Hz, 3H), 0.04 (s, 9H); ^13^C NMR (101 MHz, CDCl_3_):δ 136.2,131.2, 124.8, 115.1, 69.0, 44.8, 43.7, 37.9, 29.0, 25.7, 25.4, 19.0, 17.6, −1.9; IR (neat) cm^−1^ $\tilde{{\rm{v}}}$: 2963,1261, 1093, 1020, 866, 799, 700; HRMS (EI(+), 70 eV): C_16_H_32_OSi [M-H]^+^: calcd. 267.2222, found 267.2139; [α]_D_^20^ = +9.4 (c = 0.40, CH_2_Cl_2_); Enantiomeric excess was determined by HPLC analysis of the 3,5-nitrobenzoate derivative of the product (Chiralcel OD-H column, hexanes:i-PrOH = 99:1, 0.3 mL/min, 210 nm), t_major_ = 19.2 min, t_minor_ = 22.0 min, 97% de.

### (3S,4R)-6,8,8-trimethyl-3-(trimethylsilyl)non-1-en-4-ol (2k)

^1^H NMR (400 MHz, CDCl_3_): δ 5.87–5.72 (m, 1H), 5.04 (d, *J* = 10.2 Hz, 1H), 4.93 (d, *J* = 17.0 Hz, 1H), 3.87 (m, 1H), 1.78–1.56 (m, 2H), 1.55–1.34 (m, 2H), 1.33–1.15 (m, 2H), 1.06 (m, 1H), 0.95–0.91 (m, 3H), 0.90–0.84 (m, 9H), 0.04(s, 9H); ^13^C NMR (101 MHz, CDCl_3_):δ 136.2, 135.6, 115.1,115.0, 69.6, 69.2, 52.0, 51.2, 47.3, 47.2, 43.7, 42.2, 31.2, 31.0, 30.1, 30.0, 26.0, 25.8, 23.4, 21.9, −1.8, −2.0; IR (neat) cm^−1^ $\tilde{{\rm{v}}}$: 2954, 1626, 1471, 1366, 1249, 1023, 898, 839, 691; HRMS (EI(+), 70 eV): C_15_H_32_OSi[M-OH]^+^: calcd. 239.2222, found 239.2190; [α]_D_^20^ = +10.6 (c = 3.50, CH_2_Cl_2_); Enantiomeric excess was determined by HPLC analysis of the 3,5-nitrobenzoate derivative of the product (Chiralcel OD-H column, hexanes:i-PrOH = 99.5:0.5, 0.4 mL/min, 230 nm), t_major_ = 14.9 min, t_minor_ = 18.0 min, 95% de.

### (1R,2S)-1-phenyl-2-(trimethylsilyl)but-3-en-1-ol (2l)

^1^H NMR (400 MHz, CDCl_3_) δ 7.36–7.24 (m, 5H), 5.86 (dt, *J* = 17.1, 10.3 Hz, 1H), 5.11 (dd, *J* = 10.3, 1.9 Hz, 1H), 5.03 (ddd, *J* = 17.1, 1.8, 0.7 Hz, 1H), 4.80 (d, *J* = 8.4 Hz, 1H), 2.22 (d, *J* = 1.6 Hz 1H), 2.08 (dd, *J* = 10.2, 8.6 Hz, 1H), −0.20 (s, 9H); ^13^C NMR (101 MHz, CDCl_3_):δ 143.6, 136.6, 128.4, 127.8, 126.9, 116.0,74.5, 45.6, −2.4; IR (neat) cm^−1^ $\tilde{{\rm{v}}}$: 3458, 2952, 1626, 1248, 907,764, 699;HRMS (EI(+), 70 eV): C_13_H_20_OSi[M-OH]^+^: calcd. 203.1283., found 203.1249; [α]_D_^20^ = −16.3 (c = 1.00, CH_2_Cl_2_); HPLC (Chiralcel OD-H column,hexanes:i-PrOH = 95:5, 1 mL/min, 210 nm), t_major_ = 7.0 min, t_minor_ = 12.1 min, 93% ee. The reported value^[7]^ for the (1S,2R)-enantiomer (95% ee) is [α]_D_^25^ = +47.0 (c = 1.0; CHCl_3_).

### (1R,2S)-1-(4-bromophenyl)-2-(trimethylsilyl)but-3-en-1-ol (2m)

^1^H NMR (400 MHz, CDCl_3_): δ 7.45 (d, *J* = 8.2 Hz, 2H), 7.21 (d, *J* = 8.4 Hz, 2H), 5.82 (dt, *J* = 17.1, 10.3 Hz, 1H), 5.09 (dd, *J* = 10.3, 1.7 Hz, 1H), 4.98 (ddd, *J* = 17.1, 1.7 Hz, 0.8Hz,1H), 4.77 (d, *J* = 8.0 Hz, 1H), 2.23 (br, 1H), 1.99 (dd, *J* = 10.3, 8.1 Hz, 1H), −0.16 (s, 9H); ^13^C NMR (101 MHz, CDCl_3_):δ 142.8, 135.9, 131.4, 128.5, 121.4, 116.3,73.8, 45.5, −2.4; IR (neat) cm^−1^ $\tilde{{\rm{v}}}$: 3435, 2957,1626, 1486, 1409, 1250, 1088,1009, 909, 837, 693.08; HRMS (EI(+), 70 eV): C_13_H_19_BrOSi [M-OH]^+^: calcd. 281.0389, found 281.0356; [α]_D_^20^ = −19.6 (c = 1.10, CH_2_Cl_2_); HPLC (Chiralcel OD-H column, hexanes:i-PrOH = 95;5, 1 mL/min, 210 nm), t_minor_ = 6.2 min, t_major_ = 6.9 min, 92% ee. The reported value^[7]^ for the (1S,2R)-enantiomer (94% ee) is [α]_D_^25^ = +10.3 (c = 1.0; CHCl_3_).

### (1R,2S)-1-(2-fluorophenyl)−2-(trimethylsilyl)but-3-en-1-ol (2n)

^1^H NMR (400 MHz, CDCl_3_): δ 7.45–7.39 (m, 1H), 7.25–7.20 (m, 1H), 7.16–7.10 (m, 1H), 7.04–6.97 (m, 1H), 5.85 (dt, *J* = 17.2, 10.4 Hz, 1H), 5.17 (dd, *J* = 7.9, 2.9 Hz, 1H), 5.08 (dd, *J* = 10.3, 1.9 Hz, 1H), 4.98 (dd, *J* = 17.0, 1.8 Hz, 1H), 2.18–2.10 (m, 2H), −0.12 (s, 9H).^13^C NMR (101 MHz, CDCl_3_): δ159.8 (d, *J*_*C-F*_ = 246.4 Hz), 136.0, 130.9 (d, *J*_*C-F*_ = 13.0 Hz), 128.9 (d, *J*_*C-F*_ = 8.4 Hz), 128.4 (d, *J*_*C-F*_ = 4.5 Hz), 124.0 (d, *J*_*C-F*_ = 3.4 Hz), 116.0, 115.3 (d, *J*_*C-F*_ = 22.2 Hz), 68.0, 44.2, −2.5. IR (neat) cm^−1^ $\tilde{{\rm{v}}}$: 3359, 2925,1626, 1487, 1456,1249, 1055, 911, 841, 758, 693;HRMS (EI(+), 70 eV): C_13_H_19_FOSi [M-OH]^+^: calcd:221.1189, found:221.1157; [α]_D_^20^ = −17.5 (c = 1.00, CH_2_Cl_2_). HPLC (Chiralcel OD-H column, hexanes:i-PrOH = 95:5, 0.4 mL/min, 210 nm), t_major_ = 13.4 min, t_minor_ = 14.2 min, 90% ee.

### (1R,2S)-1-(3-fluorophenyl)-2-(trimethylsilyl)but-3-en-1-ol (2o)

^1^H NMR (400 MHz, CDCl_3_): δ 7.33–7.24 (m, 1H), 7.13–7.03 (m, 2H), 6.96 (td, *J* = 8.4, 1.8 Hz, 1H), 5.82 (dt, *J* = 17.1, 10.3 Hz, 1H), 5.10 (dd, *J* = 10.3, 1.7 Hz, 1H), 4.99 (dd, *J* = 17.1, 1.1 Hz, 1H), 4.81 (d, *J* = 7.9 Hz, 1H), 2.26 (br, 1H), 2.01 (dd, *J* = 10.2, 8.0 Hz, 1H), −0.15 (s, 9H). ^13^C NMR (101 MHz, CDCl_3_):δ 162.9 (d, *J*_*C-F*_ = 247.4 Hz), 146.4 (d, *J* = 6.7 Hz), 135.9, 129.8 (d, *J*_*C-F*_ = 8.2 Hz), 122.4 (d, *J*_*C-F*_ = 2.8 Hz), 116.3, 114.6 (d, *J*_*C-F*_ = 21.2 Hz), 113.6 (d,*J*_*C-F*_ = 22.2 Hz), 73.9, 45.5, −2.4; IR (neat) cm^−1^ $\tilde{{\rm{v}}}$: 2956, 2927, 1669, 1592, 1451,1257, 1090, 1029, 841, 801, 696; HRMS (EI(+), 70 eV): C_13_H_19_FOSi[M-H]^+^: calcd. 237.1189, found 237.1105; [α]_D_^20^ = −15.2 (c = 0.70, CH_2_Cl_2_); HPLC (Chiralcel OD-H column, hexanes:i-PrOH = 95:5, 1.0 mL/min, 210 nm), t_major_ = 6.4 min, t_minor_ = 12.4 min, 92% ee.

### (1R,2S)−1-(4-chlorophenyl)-2-(trimethylsilyl)but-3-en-1-ol (2p)

^1^H NMR (400 MHz, CDCl_3_): δ 7.34–7.22 (m, 4H), 5.83 (dt, *J* = 17.1, 10.2 Hz, 1H), 5.10 (d, *J* = 10.2 Hz, 1H), 4.99 (d, *J* = 17.2 Hz, 1H), 4.79 (d, *J* = 8.0 Hz, 1H), 2.22 (br, 1H), 2.00 (dd, *J* = 10.1, 8.3 Hz, 1H), −0.16 (s, 9H).^13^C NMR (101 MHz, CDCl_3_):δ 142.2, 136.0, 133.3, 128.5, 128.2, 116.3,73.8, 45.6, −2.4; IR (neat) cm^−1^ $\tilde{{\rm{v}}}$: 3714, 3075, 2953,1625, 1491, 1410, 1248, 1010, 911, 836, 694; HRMS (EI(+), 70 eV): C_13_H_19_ClOSi [M-OH]^+^calcd:237.0894, found:237.0861;[α]_D_^20^ = −12.3 (c = 2.00, CH_2_Cl_2_); HPLC (Chiralcel OD-H column, hexanes:i-PrOH = 95:5, 0.4 mL/min, 210 nm), t_minor_ = 14.7 min, t_major_ = 15.5 min, 91% ee.The reported value^[7]^ for the (1S,2R)-enantiomer (59% ee) is [α]_D_^25^ = +4.8 (c = 1.0; CHCl_3_).

### Materials

NMR spectra were recorded at room temperature on the following spectrometers: Agilent (400 MHz) and VARIAN (400 MHz). Chemical shifts are given in ppm and coupling constants in Hz. ^1^H spectra were calibrated in relation to the reference measurement of TMS (0.00 ppm). ^13^C spectra were calibrated in relation to deuterated solvents, namely CDCl_3_ (77.16 ppm). The following abbreviations were used for ^1^H NMR spectra to indicate the signal multiplicity: s (singlet), d (doublet), t (triplet), q (quartet) and m (multiplet) as well as combinations of them. When combinations of multiplicities are given the first character noted refers to the largest coupling constant. High performance liquid chromatography (HPLC) was carried out with Agilent 1260 Infinity on a UV spectrophotometric detector (210 nm, Agilent). For ESI^+^-spectra and EI^-^HR (GC-TOF) spectrometer was applied. Infrared Spectroscopy (IR) was processed on an FT-IR spectrometer named Nicolet 380. The method is denoted in brackets. For the most significant bands the wave number $\tilde{{\rm{v}}}$: (cm^−1^) is given.

Chemicals were purchased from commercial suppliers. Unless stated otherwise, all the substrates and solvents were purified and dried according to standard methods prior to use. Reactions requiring inert conditions were carried out in glove box.

## Electronic supplementary material

Dataset 1
