# Supplementary material for: Barbier-type anti-Diastereo- and Enantioselective Synthesis of β-Trimethylsilyl, Fluorinated Methyl, Phenylthio Homoallylic Alcohols
Source: Sci Rep. 2017 Jul 7;7:4873. doi: 10.1038/s41598-017-04986-x (PMC5501818; doi:10.1038/s41598-017-04986-x)
Supplement: Supplementary file 1 — Dataset 1 [file 41598_2017_4986_MOESM1_ESM.doc]

# SUPPORTING INFORMATION

Barbier-type *anti*-Diastereo- and Enantioselective Synthesis of β-Trimethylsilyl, Fluorinated Methyl, Phenylthio Homoallylic Alcohols

Rui Guo, Qin Yang, Qinshan, Tian, Guozhu Zhang

State Key Laboratory of Organometallic Chemistry, Shanghai Institute of Organic Chemistry, Chinese Academy of Sciences, 345 Lingling Road, Shanghai 200032, P. R. China

**CONTENTS**

1. General Information **S2**
2. Experimental Details **S3**

(1).Preparation and Characterization of Ligands **S3**

(2).Preparation and Characterization of Materials **S3**

(3).General Procedure **S5**

(4).The Characterization of Products **S7**

(5).Reference **S33**

3. Copies of NMR Spectra and HPLC Chromatographs **S34**

1. **General Information:**

NMR spectra were recorded at room temperature on the following spectrometers: Agilent (400 MHz) and VARIAN (400 MHz). Chemical shifts are given in ppm and coupling constants in Hz. ^1^H spectra were calibrated in relation to the reference measurement of TMS (0.00 ppm). ^13^C spectra were calibrated in relation to deuterated solvents, namely CDCl_3_ (77.16 ppm). The following abbreviations were used for ^1^H NMR spectra to indicate the signal multiplicity: s (singlet), d (doublet), t (triplet), q (quartet) and m (multiplet) as well as combinations of them. When combinations of multiplicities are given the first character noted refers to the largest coupling constant. High performance liquid chromatography (HPLC) was carried out with Agilent 1260 Infinity on a UV spectrophotometric detector (210 nm, Agilent). For ESI^+^-spectra and EI^-^HR (GC-TOF) spectrometer was applied. Infrared Spectroscopy (IR) was processed on an FT-IR spectrometer named Nicolet 380. The method is denoted in brackets. For the most significant bands the wave number ṽ (cm^-1^) is given.

Chemicals were purchased from commercial suppliers. Unless stated otherwise, all the substrates and solvents were purified and dried according to standard methods prior to use. Reactions requiring inert conditions were carried out in glove box.

1. **Experimental Details**
2. **Preparation and Characterization of Ligands：**

Symmertric bis(dihydrooxazol)carbazole ligands^[1]^ **L-1**, **L-2**, **L-3**, **L-4**, **L-6, L-7** and symmertric bisoxazoline ligand^[2]^ **L-8** were synthesized and characterized according to the literatures reported as known compounds.

**1,8-bis((S)-4-((R)-sec-butyl)-4,5-dihydrooxazol-2-yl)-9H-carbazole (L-5)**

According to the literatures reported^[1]^, ligand **L-5** was prepared from 3-chloro-2-((2-cyanophenyl)amino)benzonitrile and (2S)-2-amino-3-methylpentan-1-ol over two steps as yellow solid.

^1^H NMR (400 MHz, CDCl_3_) δ 12.02 (s, 1H), 8.18 (d, *J* = 7.7 Hz, 2H), 7.91 (dd, *J* = 7.6, 0.7 Hz, 2H), 7.25 (t, *J* = 7.6 Hz, 2H), 4.46 (dd, *J* = 9.5, 8.0 Hz, 2H), 4.37-4.28 (m, 2H), 4.17 (t, *J* = 7.9 Hz, 2H), 1.94-1.82 (m, 2H), 1.74 (d, *J* = 20.0 Hz, 2H), 1.37-1.23 (m, 2H), 1.00 (t, *J* = 7.4 Hz, 6H), 0.95 (d, *J* = 6.7 Hz, 6H); ^13^C NMR (101 MHz, CDCl_3_) δ 162.54, 139.03, 125.81, 123.52, 123.43, 118.78, 110.61, 71.82, 69.56, 39.89, 26.38, 14.88, 11.91; IR (neat) cm^-1^ ṽ: 3344, 2963, 1643, 1597, 1492, 1369, 1275, 1210, 1134,1052, 983, 790, 750, 707; HRMS (EI(+), 70 eV) : C_26_H_31_N_3_O_2_ [M]^+^: calcd. 417.2416, found 417.2418; [α]_D_^20^ = 172.6 (c = 1.67, CH_2_Cl_2_).

1. **Preparation and Characterization of Materials**

**Preparation of (E)-4,4,4-trifluorobut-2-en-1-ol (1):**^[3]^ To a suspension of anhydrous AlCl_3_ (3.37 g, 25.3 mmol) in diethyl ether (50 mL) at 0°C was added LiAlH_4_ (2.82 g,74.4 mmol)dropwise. The resulting mixture was stirred at this temperature for 15 min. A solution of(E)-ethyl 4,4,4-trifluorobut-2-enoate (4.44 mL, 29.74 mmol) in diethyl ether (10 mL) was then added at 0°C and stirring was continued for a further 4 h. The reaction mixture was subsequently quenched with 2.82 mL water, then added 2.82 mL NaOH aqueous solution (30%) and 9 mL water at 0°C. The resulting mixture was stirred at room temperature for 30 min and filtered, and the solids were washed with diethyl ether. The combined ethereal phases were concentrated in vacuo to give crude (E)-4,4,4-trifluorobut-2-en-1-ol (3.4 g, 27 mmol) without further purification.

**Preparationof (E)-4-bromo-1,1,1-trifluorobut-2-ene:**^[3]^ To a solution of (E)-4,4,4-trifluorobut-2-en-1-ol (3.4 g, 27 mmol) in diethyl ether (20 mL) at -10°C was slowly added PBr_3_ (3.9 mL, 41 mmol). The reaction mixture was stirred for 1 h at this temperature and then allowed to warm to room temperature. After stirring for a further 12 h, water was slowly added to quench the reaction.The product was extracted with diethyl ether, washed with water and brine, and dried with anhydrous MgSO_4_. After filtration, the ethereal solution was fractionally distilled to afford (E)-4-bromo-1,1,1-trifluorobut-2-ene (2.0 g, 10.6 mmol, 37% yield, two steps); ^1^H NMR (400 MHz, CDCl_3_) δ 6.61 – 6.46 (m, 1H), 5.97 – 5.85 (m, 1H), 3.98 (d, *J* = 6.8 Hz).

**Preparation of (E)-4,4-difluorobut-2-en-1-ol:**To a suspension of anhydrous AlCl_3_ (1.74 g, 13 mmol) in diethyl ether (20 mL) at 0°C was added LiAlH_4_ (1.45 g, 38 mmol) dropwise. The resulting mixture was stirred at this temperature for 15 min. A solution of ethyl 4,4-difluorocrotonate^[2]^ (2.3 g, 15.3 mmol) in diethyl ether (5 mL) was then added at 0°C and stirring was continued for a further 4 h. The reaction mixture was subsequently quenched with 1.5 mL water, then added 1.5 mL NaOH aqueous solution (30%) and 4.5 mL water at 0°C. The resulting mixture was stirred at room temperature for 30 min and filtered, and the solids were washed with diethyl ether. The combined ethereal phases were concentrated in vacuo to give crude (E)-4,4-difluorobut-2-en-1-ol (1.73 g, 16.0 mmol) without further purification.

**Preparation of (E)-4-bromo-1,1-difluorobut-2-ene**: To a solution of 1,1,1-trifluoro-4-hydroxylbut-2-ene (1.73 g, 16.0 mmol) in diethyl ether (10 mL) at -10°C was slowly added PBr_3_ (3.0 mL, 32 mmol). The reaction mixture was stirred for 1 h at this temperature and then allowed to warm to room temperature. After stirring for a further 12 h, water was slowly added to quench the reaction.The product was extracted with diethyl ether, washed with water and brine, and dried with anhydrous MgSO_4_andconcentrated.The residue was purified by distillation to afford pure (E)-4-bromo-1,1-difluorobut-2-ene (1.27 g, 7.4 mmol, 48% yield, two steps); ^1^H NMR (300 MHz, CDCl_3_) δ 6.43 – 5.65 (m, 3H), 4.00 – 3.89 (m, 2H); ^19^F NMR (376 MHz, CDCl_3_) δ -112.37- -112.62 (m, 2F); ^13^C NMR (100 MHz, CDCl_3_) δ 133.9 (t, *J* = 12.2Hz), 126.6 (t, *J* = 23.6Hz), 113.6 (t, *J* = 227.7Hz), 29.2; HRMS (EI(+), 70 eV) :C_4_H_5_F_2_Br[M]^+^: calcd. 169.9543, found 169.9549; IR (neat) cm^-1^ ṽ:2964, 2918, 1409, 1262, 1095, 1024, 865, 803, 698.

**Preparation of(E)-4-fluorobut-2-en-1-ol:**To a suspension of ethyl 4-fluorobut-2-enoate^[4,5]^ (2.5 g, 18.9 mmol) in diethyl ether (16 mL) at - 78°C was added DIBAL-H (1.5 M in toluene) (28 mL,42 mmol) dropwise. The resulting mixture was stirred at this temperature for 4h.

The reaction mixture was quenched was quenched by24 mL MeOH and 1.5 mL NaOHaqueous solution (30%), then the mixture was extracted with diethyl ether, washed with brine, and dried with anhydrous MgSO_4_, then concentrated to give crude (E)-4-fluorobut-2-en-1-ol (1.34 g, 14.9 mmol) without further purification.

**Preparation of (E)-4-bromo-1,1-difluorobut-2-ene**:To a solution of (E)-4-fluorobut-2-en-1-ol (1.29 g, 14.3 mmol) in diethyl ether (8 mL) at -10°C was slowly added PBr_3_ (2.7 mL, 28.7 mmol). The reaction mixture was stirred for 1 h at this tem- perature and then allowed to warm to room temperature. After stirring for a further 12 h, water was slowly added to quench the reaction. The product was extracted with diethyl ether, washed with water and brine, and dried with anhydrous MgSO_4_andconcentrated.The residue was purified by distillation to afford pure (E)-1-bromo-4-fluorobut-2-ene (0.87 g, 5.71 mmol, 31% yield, two steps); ^1^H NMR (400 MHz, CDCl_3_) δ 6.12 – 5.83 (m, 2H), 4.94 (d, *J* = 5.2 Hz, 1H), 4.83 (d, *J* = 5.2 Hz, 1H), 4.00 – 3.93 (m, 2H); ^19^F NMR (376 MHz, CDCl_3_)δ -215.32 - -215.69 (m, 1F); ^13^C NMR (100 MHz, CDCl_3_) δ 129.9 (d, *J* = 12.3 Hz), 129.2 (d, *J* = 16.8 Hz), 81.9 (d, *J* = 163.9 Hz), 31.0; HRMS (EI(+), 70 eV) : C_4_H_6_FBr[M]^+^: calcd. 151.9637, found 151.9639; IR (neat) cm^-1^ ṽ: 2932, 2862, 1667, 1378, 1293, 1250, 1209, 1081, 1014, 797, 691.

1. **General Procedure:**

**method A:** To a mixture of anhydrous chromium(II) chloride (1.2 mg, 0.01mmol, 5.0 mmol%), 1,8-bis((S)-4-((R)-sec-butyl)-4,5-dihydrooxazol-2-yl)-9H-carbazole (**L-5**, 9.0 mg,0.022mmol, 10.8mmol%) and Proton sponge (6.0mg, 0.028 mmol, 14.0 mmol%) was added THF (1.0 ml) under an nitrogen atmosphere. The mixture was stirred vigorously at room temperature for 3hours before it was transferred into a vessel charged with Zr(Cp)_2_Cl_2_ (60.0 mg, 0.2 mmol, 1.0 eq.), LiCl (8.4 mg, 0.2 mmol, 1.0 eq.) and Manganese powder (22.0 mg, 0.4mmol, 2.0 eq.). Then (E)-(3-bromoprop-1-en-1-yl)trimethylsilane (77 mg, 0.4mmol, 2.0 eq.) and aldehyde (0.2mmol, 1.0 eq.) were added in succession. The resulting suspension was left stirred at room temperature overnight. After the full consumption of aldehyde, the reaction mixture was diluted with undried EA and the resulting suspension was filtered over a pad of silica gel using EA as eluent. Volatiles were evaporated in vacuo. The residue was purified by chromatographyafforded the product.

**method B:** To a mixture of anhydrous chromium(II) chloride (1.2 mg, 0.01 mmol, 5.0mmol%), 1,8-bis((S)-4-((R)-sec-butyl)-4,5-dihydrooxazol-2-yl)-9H-carbazole ( **L-5**, 9.0 mg, 0.022mmol, 10.8mmol%) and Proton sponge (6.0 mg, 0.028 mmol, 14.0 mmol%) was added CH_3_CN (1.0 ml) under an nitrogen atmosphere. The mixture was stirred vigorously at room temperature for 3 hours before it was transferred into a vessel charged with Zr(Cp)_2_Cl_2_ (60.0 mg, 0.2 mmol, 1.0 eq.), LiCl (8.4 mg, 0.2 mmol, 1.0 eq.) and Manganese powder (22.0 mg, 0.4 mmol, 2.0 eq.). Then (E)-(3-bromoprop-1-en-1-yl)trimethylsilane (77 mg, 0.4 mmol, 2.0 eq.) and aldehyde (0.2 mmol, 1.0 eq.) were added in succession. The resulting suspension was left stirred at room temperature overnight. After the full consumption of aldehyde, the reaction mixture was diluted with undried EA and the resulting suspension was filtered over a pad of silica gel using EA as eluent. Volatiles were evaporated in vacuo. The residue was purified by chromatographyafforded the product.

**method C:** To a mixture of anhydrous chromium(II) chloride (1.2 mg, 0.01mmol, 5.0mmol%), 1,8-bis((S)-4-isopropyl-4,5-dihydrooxazol-2-yl)-9H-carbazole (**L-1**, 7.8 mg,0.020 mmol, 10.0 mmol%) and Proton sponge (4.4 mg,0.021 mmol, 10.2 mmol%) was added THF (0.4 ml) under an nitrogen atmosphere. The mixture was stirred vigorously at room temperature for 3hours before it was transferred into a vessel charged with Zr(Cp)_2_Cl_2_ (58.5 mg, 0.2 mmol, 1.0 eq.), CsI (52.0 mg, 0.2 mmol, 1.0 eq.) and Manganese powder (13.2 mg, 0.24mmol, 1.2 eq.). Then(E)-4-bromo-1,1,1-trifluorobut-2-ene (75.6 mg, 0.4 mmol, 2.0 eq.) and aldehyde (0.2 mmol, 1.0 eq.) were added in succession. The resulting suspension was left stirred at room temperature overnight. After the full consumption of aldehyde, the reaction mixture was diluted with undried EA and the resulting suspension was filtered over a pad of silica gel using EA as eluent. Volatiles were evaporated in vacuo. The residue was purified by chromatographyafforded the product.

**method D:** To a mixture of anhydrous chromium(II) chloride (1.2 mg, 0.01mmol, 5.0mmol%), 1,8-bis((S)-4-isopropyl-4,5-dihydrooxazol-2-yl)-9H-carbazole (**L-1**, 7.8 mg,0.020mmol, 10.0mmol%) and Proton sponge (4.4mg,0.021mmol, 10.2mmol%) was added DME (0.4 ml) under an nitrogen atmosphere. The mixture was stirred vigorously at room temperature for 3hours before it was transferred into a vessel charged with Zr(Cp)_2_Cl_2_ (58.5 mg, 0.2 mmol, 1.0 eq.), CsI (52.0 mg, 0.2 mmol, 1.0 eq.) and Manganese powder (13.2 mg, 0.24 mmol, 1.2 eq.). Then (E)-4-bromo-1,1,1-trifluorobut-2-ene (75.6 mg, 0.4 mmol, 2.0 eq.) and aldehyde (0.2 mmol, 1.0 eq.) were added in succession. The resulting suspension was left stirred at room temperature overnight. After the full consumption of aldehyde, the reaction mixture was diluted with undried EA and the resulting suspension was filtered over a pad of silica gel using EA as eluent. Volatiles were evaporated in vacuo. The residue was purified by chromatographyafforded the product.

**method E:** To a mixture of anhydrous chromium(II) chloride (0.6 mg, 0.005 mmol, 5.0 mmol%), 1,8-bis((S)-4-isopropyl-4,5-dihydrooxazol-2-yl)-9H-carbazole (**L-1**, 3.9 mg,0.010 mmol, 10.0 mmol%) and Proton sponge (2.8 mg, 0.013 mmol, 13 mmol%) was added DME (0.5 ml) under an nitrogen atmosphere. The mixture was stirred vigorously at room temperature for 3 hours before it was transferred into a vessel charged with Zr(Cp)_2_Cl_2_ (30 mg, 0.1 mmol, 1.0 eq.), and Manganese powder (11 mg, 0.20 mmol, 2.0 eq.). Then (3-chloroprop-1-en-1-yl)(phenyl)sulfane (E/Z=10:1) (36.8 mg, 0.2 mmol, 2.0 eq.) and aldehyde (0.1 mmol, 1.0 eq.) were added in succession. The resulting suspension was left stirred at room temperature overnight. After the full consumption of aldehyde, the reaction mixture was diluted with undried EA and the resulting suspension was filtered over a pad of silica gel using EA as eluent. Volatiles were evaporated in vacuo. The residue was purified by chromatographyafforded the product.

The ee was determined by HPLC analysis with Chiralcel OD and IE et al. columns. The absolute configuration was assigned by comparing the optical rotation with that of reported examples.^[6,7,8,9]^

1. **The Characterization of Products:**

**(3R,4S)-1-phenyl-4-(trimethylsilyl)hex-5-en-3-ol (2a)**

According to the general procedure with method A, **2a** (46.2 mg, 0.186 mmol) was prepared from Phenylpropyl aldehyde (26.8 mg, 0.20 mmol) as yellow oil in 93% yield.

^1^H NMR (400 MHz, CDCl_3_): δ 7.25 -7.17 (m, 2H), 7.16 – 7.05 (m, 3H), 5.73 (dt, *J* = 17.1, 10.4 Hz, 1H), 4.98 (dd,*J* = 10.4, 1.6 Hz,1H), 4.88 (d, *J* = 17.1Hz,1H), 3.75 (dd, *J* = 12.7, 5.2 Hz, 1H), 2.72 – 2.53 (m, 2H), 1.79 – 1.68 (m, 2H), 1.65 (dd, *J* = 10.6, 5.4 Hz, 1H), 1.54 (br, 1H), -0.02 (s, 9H); ^13^C NMR (101 MHz, CDCl_3_):δ 142.1, 135.7, 128.4, 128.3, 125.7, 115.2, 71.0, 42.6, 39.0, 32.3, -2.0; IR (neat) cm^-1^ ṽ: 3456,3027,1247,899,839,749, 698;HRMS (EI(+), 70 eV) : C_15_H_24_OSi [M-H]^+^: calcd. 247.1596, found.247.1513; [α]_D_^20^ = + 23.2 (c = 1.00, CH_2_Cl_2_); HPLC (Chiralcel OD-H column, hexanes:i-PrOH = 95:5, 0.5 mL/min, 210 nm), t_minor_ = 9.8 min, t_major_ = 12.1 min, 95% ee.

**(3S,4R)-3-(trimethylsilyl)dec-1-en-4-ol (2b)**

According to the general procedure with method A, **2b** (43.4 mg, 0.19 mmol) was prepared from Heptaldehyde (22.8 mg, 0.20 mmol) as yellow oil in 95% yield.

^1^H NMR (400 MHz, CDCl_3_): δ 5.79 (dt, *J* = 17.1, 10.4 Hz, 1H), 5.03 (dd, *J* = 10.2, 2.1 Hz, 1H), 4.92 (dd, *J* = 17.1, 2.0 Hz, 1H), 3.79 (dt, *J* = 7.5, 5.1 Hz, 1H), 1.67 (dd, *J* = 10.6, 5.3 Hz, 1H), 1.52 – 1.36 (m, 3H), 1.35 – 1.20 (m, 8H), 0.88 (t, *J* = 6.7 Hz, 3H), 0.04 (s, 9H); ^13^C NMR (101 MHz, CDCl_3_):δ 135.9, 115.0, 71.6, 42.6, 37.3, 31.8, 29.3, 25.8, 22.6, 14.1, -2.0.

IR (neat) cm^-1^ ṽ: 3696, 2962,2856, 1261, 1093, 1021, 800, 690; HRMS (EI(+), 70 eV) : C_13_H_28_OSi [M-H]^+^: calcd. 227.1909, found 227.1826; [α]_D_^20^ = +2.8 (c = 2.00, CH_2_Cl_2_); Enantiomeric excess was determined by HPLC analysis of the 3,5-nitrobenzoate derivative of the product (Chiralcel OD-H column, hexanes:i-PrOH = 98:2, 0.4 mL/min, 210 nm), t_major_ = 13.9 min, t_minor_ = 15.3 min, 96% ee.

**(1R,2S)-1-cyclohexyl-2-(trimethylsilyl)but-3-en-1-ol (2c)**

According to the general procedure with method B, **2c** (37.1 mg, 0.164 mmol) was prepared from cyclohexanecarboxaldehyde (22.4 mg, 0.20 mmol) as yellow oil in 82% yield.

^1^H NMR (400 MHz, CDCl_3_): δ 5.82 (dt, *J* = 17.1, 10.5 Hz, 1H), 5.00 (dd, *J* = 10.2, 2.2 Hz, 1H), 4.89 (dd, *J* = 17.1, 2.0 Hz, 1H), 3.43 (dd, *J* = 7.2, 4.5 Hz, 1H),1.91 –1.82 (m, 2H), 1.79 – 1.58 (m, 4H), 1.42 – 1.32 (m, 1H), 1.30 – 1.05 (m, 4H), 0.95 (dtd, *J* = 15.8, 12.3, 2.6 Hz, 2H), 0.05 (d, *J* = 11.7 Hz, 9H); ^13^C NMR (101 MHz, CDCl_3_):δ 135.6, 114.4,76.2, 42.4, 39.3, 29.3, 28.5, 26.4, 26.2, 25.9, -2.1; IR (neat) cm^-1^ ṽ: 3492, 2926,2854, 1450, 1248, 1037, 895, 839, 693; HRMS (EI(+), 70 eV) : C_13_H_26_OSi[M-OH]^+^: calcd. 209.1753, found: 209.1720;[α]_D_^20^ = -6.1 (c = 0.20, CH_2_Cl_2_); Enantiomeric excess was determined by HPLC analysis of the 3,5-nitrobenzoate derivative of the product (Chiralcel OD-H column, hexanes:i-PrOH = 99:1, 0.3 mL/min, 210 nm), t_major_ = 23.0 min, t_minor_ = 25.2 min, 92% ee.

**(3S,4R)-8-chloro-3-(trimethylsilyl)oct-1-en-4-ol (2d)**

According to the general procedure with method A, **2d** (43.1 mg, 0.184 mmol) was prepared from 5-chloropentanal (24.0 mg, 0.20 mmol) as yellow oil in 92% yield.

^1^H NMR (400 MHz, CDCl_3_): δ 5.78 (dt, *J* = 17.1, 10.4 Hz, 1H), 5.04 (dd, *J* = 10.2, 2.0 Hz, 1H), 4.93 (dd, *J* = 17.1, 1.8 Hz, 1H), 3.79 (d, *J* = 4.6 Hz, 1H), 3.53 (t, *J* = 6.7 Hz, 2H), 1.85 – 1.73 (m, 2H), 1.71 – 1.60 (m, 2H), 1.59 – 1.40 (m, 4H), 0.04 (s, 9H).^13^C NMR (101 MHz, CDCl_3_): δ 135.6, 115.3, 71.3, 45.0, 42.6, 36.4, 32.5, 23.2, -2.0. IR (neat) cm^-1^ ṽ: 3463, 2959, 1625, 1449, 1412, 1256, 1089, 1014, 838, 796, 692; HRMS (EI(+), 70 eV) : C_11_H_23_ClOSi [M-H]^+^: calcd. 233.1207, found 233.1124; [α]_D_^20^ = +1.5 (c = 1.50, CH_2_Cl_2_); Enantiomeric excess was determined by HPLC analysis of the 3,5-nitrobenzoate derivative of the product (Chiralcel OD-H column, hexanes:i-PrOH = 95:5, 0.5 mL/min, 210 nm), t_major_ = 14.7 min, t_minor_ = 19.5 min, 97% ee.

**(3S,4R)-3-(trimethylsilyl)octa-1,7-dien-4-ol (2e)**

According to the general procedure with method A, **2e** (37.3 mg, 0.188 mmol) was prepared from 4-pentenal (16.8 mg, 0.20 mmol) as yellow oil in 94% yield.

^1^H NMR (400 MHz, CDCl_3_): δ 5.93 – 5.69 (m, 2H), 5.16 – 4.82 (m, 4H), 3.82 (dd, *J* = 12.6, 5.3 Hz, 1H), 2.24 – 2.06 (m, 2H), 1.67 (dd, *J* = 10.6, 5.2 Hz, 1H), 1.64 – 1.49 (m, 3H), 0.04 (s, 9H); ^13^C NMR (101 MHz, CDCl_3_):δ 138.5, 135.8, 115.1, 114.7, 71.0, 42.7, 36.3, 30.3, -2.0; IR (neat) cm^-1^ ṽ: 3359, 2922, 2852, 1734, 1658, 1279, 1253, 1087, 801, 700; HRMS (EI(+), 70 eV) : C_11_H_22_OSi[M-H]^+^: calcd. 197.1440, found197.1368; [α]_D_^20^ = + 5.3 (c = 0.20, CH_2_Cl_2_); Enantiomeric excess was determined by HPLC analysis of the 3,5-nitrobenzoate

derivative of the product (Chiralcel OD-H column, hexanes:i-PrOH = 98:2, 0.4 mL/min, 210 nm), t_major_ = 17.0 min, t_minor_ = 20.2 min, 97% ee. The optical rotation is coincident with the reported example^[6]^.

**(1R,2S)-1-(cyclohex-3-en-1-yl)-2-(trimethylsilyl)but-3-en-1-ol (2f)**

According to the general procedure with method B, **2f** (37.2 mg, 0.166 mmol) was prepared from 3-cyclohexene-1-carboxaldehyde (22.0 mg, 0.20 mmol) as yellow oil in 83% yield.

^1^H NMR (400 MHz, CDCl_3_): δ 5.84 (dt, *J* = 17.7, 10.4 Hz, 1H), 5.74 – 5.57 (m, 2H), 5.01 (d, *J* = 10.2 Hz, 1H), 4.91 (d, *J* = 17.1 Hz, 1H), 3.53 (d, *J* = 34.4 Hz, 1H), 2.21 – 1.79 (m, 5H), 1.77 – 1.38 (m, 4H), 0.05 (s, 9H).^13^C NMR (101 MHz, CDCl_3_):δ 135.4, 135.2, 127.0, 126.2, 126.1, 114.6, 75.6, 75.6, 39.6, 39.3, 38.6, 38.5,28.0, 27.4, 25.2, 25.1,24.5, -2.1, -2.2.

; IR (neat) cm^-1^ ṽ: 2963, 1261, 1091, 1020, 866, 799, 700; HRMS (EI(+), 70 eV) : C_13_H_24_OSi [M-H]^+^: calcd. 223.1596, found 223.1514; [α]_D_^20^ = -1.2 (c = 0.40, CH_2_Cl_2_). Enantiomeric excess was determined by HPLC analysis of the 3,5-nitrobenzoate derivative of the product (Chiralcel OD-H column, hexanes:i-PrOH = 99.5:0.5, 0.4 mL/min, 210 nm), t_major_ = 26.8 min, 27.8 min, t_minor_ = 29.5 min, 31.3 min, 90% de.

**(3R,4S)-1-((tert-butyldiphenylsilyl)oxy)-4-(trimethylsilyl)hex-5-en-3-ol (2g)**

According to the general procedure with method B, **2g** (75 mg, 0.176mmol) was prepared from 3-((tert-butyldiphenylsilyl)oxy)propanal (62.4 mg, 0.20 mmol) as yellow oil in 88% yield.

^1^H NMR (400 MHz, CDCl_3_): δ 7.71 (dd, *J* = 7.6, 1.4 Hz, 4H), 7.50 – 7.38 (m, 6H), 5.93 (dt, *J* = 17.1, 10.4 Hz, 1H), 5.01 (dd, *J* = 10.3, 2.3 Hz, 1H), 4.89 (ddd, *J* = 17.1, 2.2, 0.6 Hz, 1H), 4.17 (ddd, *J* = 9.3, 4.1, 2.9 Hz, 1H), 3.92 – 3.78 (m, 2H), 2.75 (s, 1H), 1.90 – 1.78 (m, 1H), 1.64 (dd, *J* = 10.6, 4.4 Hz, 1H), 1.62 – 1.55 (m, 1H), 1.08 (s, 9H), 0.09 (s, 9H).^13^C NMR (101 MHz, CDCl_3_): δ 136.2, 135.6, 135.5, 133.2, 133.1, 129.8, 129.7, 127.7, 114.0, 70.9, 63.2, 43.2, 39.0, 26.8, 19.0, -2.1; IR (neat) cm^-1^ ṽ: 3522, 3071, 2955, 1624, 1469, 1426, 1390, 1248, 1081, 899, 837, 738, 702; HRMS (EI(+), 70 eV) : C_25_H_38_O_2_Si_2_[M+H]^+^: calcd. 427.2410, found 427.2484; [α]_D_^20^ = + 0.8 (c = 1.00, CH_2_Cl_2_); HPLC (Chiralcel OD-H column, hexanes:i-PrOH = 99:1, 0.4 mL/min, 210 nm), t_major_ = 9.5 min, t_minor_ = 10.1 min, 97% ee.

**2-((3R,4S)-3-hydroxy-4-(trimethylsilyl)hex-5-en-1-yl)isoindoline-1,3-dione (2h)**

According to the general procedure with method B, **2h** (43.8 mg, 0.138 mmol) was prepared from 3-(1,3-dioxoisoindolin-2-yl)propanal^[10]^ (40.6mg, 0.20mmol) as yellow oil in 69% yield.

^1^H NMR (400 MHz, CDCl_3_): δ 7.85 (dd, *J* = 5.4, 3.1 Hz, 2H), 7.72 (dd, *J* = 5.4, 3.0 Hz, 2H), 5.82 (dt, *J* = 17.1, 10.4 Hz, 1H), 5.01 (dd, *J* = 10.2, 2.0 Hz, 1H), 4.89 (dd, *J* = 17.1, 1.8 Hz, 1H), 3.91 – 3.72 (m, 3H), 2.64 (d, *J* = 4.5 Hz, 1H), 1.84 (ddd, *J* = 15.0, 10.2, 5.2 Hz, 1H), 1.78 – 1.68 (m, 1H), 1.62 (dd, *J* = 10.6, 4.0 Hz, 1H), 0.00 (d, *J* = 6.1 Hz, 9H); ^13^C NMR (101 MHz, CDCl_3_): δ 174.6, 168.9, 135.5, 134.0, 132.0, 123.3, 115.0, 68.3, 42.5, 36.1, 35.1, -2.2; IR (neat) cm^-1^ ṽ: 3503, 2957,1707, 1621,1251, 1049, 838, 794, 719;HRMS (EI(+), 70 eV) : C_17_H_23_NO_3_Si[M-H]^+^: calcd. 316.1447, found 316.1364;[α]_D_^20^ = -2.9 (c = 1.00, CH_2_Cl_2_).

HPLC (Chiralcel OD-H column, hexanes:i-PrOH = 98:2, 0.4 mL/min, 210 nm), t_major_ = 27.5 min, t_minor_ = 29.8 min, 98% ee.

**(2R,3S)-1-(2,2-dimethyl-1,3-dioxolan-4-yl)-3-(trimethylsilyl)pent-4-en-2-ol (2i)**

According to the general procedure with method A, **2i** (41.3 mg, 0.160 mmol) was prepared from 2-(2,2-dimethyl-1,3-dioxolan-4-yl)acetaldehyde^[11]^ (28.8 mg, 0.20 mmol) as yellow oil in 80% yield (**minor**: **major** = 2: 3).

**Minor**: ^1^H NMR (400 MHz, CDCl_3_): δ 5.92 (dt, *J* = 17.2, 10.4 Hz, 1H), 4.98 (dd, *J* = 10.2, 2.1 Hz, 1H), 4.86 (dd, *J* = 17.2, 2.2 Hz, 1H), 4.25 (ddd, *J* = 10.1, 8.4, 3.3 Hz, 1H), 4.07 (dd, *J* = 8.0, 6.0 Hz, 2H), 3.54 (t, *J* = 7.7 Hz, 1H), 3.05 (br, 1H), 1.75 (dt, *J* = 14.2, 9.8 Hz, 1H), 1.61 – 1.51 (m, 2H), 1.41 (s, 3H), 1.36 (s, 3H), 0.05 (s, 9H); ^13^C NMR (101 MHz, CDCl_3_): δ 135.8, 114.0, 109.4, 76.1, 71.1, 69.8, 43.3, 40.6, 26.9, 25.8, -2.2.

**Major**: ^1^H NMR (400 MHz, CDCl_3_): δ 5.80 (dt, *J* = 17.1, 10.4 Hz, 1H), 5.04 (dd, *J* = 10.3, 2.0 Hz, 1H), 4.94 (dd, *J* = 17.1, 2.0 Hz, 1H), 4.39 – 4.28 (m, 1H), 4.08 (dd, *J* = 8.1, 6.1 Hz, 2H), 3.58 (t, *J* = 7.8 Hz, 1H), 2.14 (br, 1H), 1.73 – 1.69 (m, 1H), 1.69 – 1.60 (m, 2H), 1.41 (s, 3H), 1.36 (s, 3H), 0.05 (s, 9H); ^13^C NMR (101 MHz, CDCl_3_): δ 136.0, 115.3, 108.6, 73.8, 69.5, 68.3, 43.8, 40.5, 26.9, 25.6, -1.9. IR (neat) cm^-1^ ṽ: 3500, 3074, 2934,1625, 1457, 1375, 1247, 1061, 838, 693;HRMS (EI(+), 70 eV) : C_13_H_26_O_3_Si[M-H]^+^: calcd. 257.1651, found 257.1569; [α]_D_^20^ = + 9.3 (c = 2.60, CH_2_Cl_2_). Enantiomeric excess was determined by HPLC analysis of the 3,5-nitrobenzoate derivative of the product (Chiralcel OD-H column, hexanes:i-PrOH = 80:20, 1 mL/min, 210 nm), t_minor_ = 4.8 min, 5.9 min, t_major_ = 5.2 min, 10.7 min, >97% de.

**(3S,4R,6S)-6,10-dimethyl-3-(trimethylsilyl)undeca-1,9-dien-4-ol (2j)**

According to the general procedure with method A, **2j** (48.8 mg, 0.182 mmol) was prepared from citronellal(30.9 mg, 0.20 mmol) as yellow oil in 91% yield.

^1^H NMR (400 MHz, CDCl_3_): δ 5.78 (dt, *J* = 17.1, 10.5 Hz, 1H), 5.10 (t, *J* = 7.1 Hz, 1H), 5.04 (dd, *J* = 10.3, 2.0 Hz, 1H), 4.93 (dd, *J* = 17.0, 1.5 Hz, 1H), 3.95 –3.85 (m, 1H), 2.04 – 1.93 (m, 2H), 1.72 – 1.57 (m, 8H), 1.50 – 1.46 (m, 1H), 1.35 – 1.27 (m, 2H), 1.23 – 1.13 (m, 2H), 0.90 (d, *J* = 6.6 Hz, 3H), 0.04 (s, 9H); ^13^C NMR (101 MHz, CDCl_3_):δ 136.2,131.2, 124.8, 115.1, 69.0, 44.8, 43.7, 37.9, 29.0, 25.7, 25.4, 19.0, 17.6, -1.9; IR (neat) cm^-1^ ṽ: 2963,1261, 1093, 1020, 866, 799, 700; HRMS (EI(+), 70 eV) : C_16_H_32_OSi [M-H]^+^: calcd. 267.2222, found 267.2139; [α]_D_^20^ = + 9.4 (c = 0.40, CH_2_Cl_2_); Enantiomeric excess was determined by HPLC analysis of the 3,5-nitrobenzoate derivative of the product (Chiralcel OD-H column, hexanes:i-PrOH = 99:1, 0.3 mL/min, 210 nm), t_major_ = 19.2 min, t_minor_ = 22.0 min, 97% de.

**(3S,4R)-6,8,8-trimethyl-3-(trimethylsilyl)non-1-en-4-ol (2k)**

According to the general procedure with method A, **2k** (48.7 mg, 0.19 mmol) was prepared from 3,5,5-trimethylhexanal (28.4 mg, 0.20 mmol) as yellow oil in 95% yield.

^1^H NMR (400 MHz, CDCl_3_): δ 5.87 – 5.72 (m, 1H), 5.04 (d, *J* = 10.2 Hz, 1H), 4.93 (d, *J* = 17.0 Hz, 1H), 3.87 (m, 1H), 1.78 – 1.56 (m, 2H), 1.55 – 1.34 (m, 2H), 1.33 – 1.15 (m, 2H), 1.06 (m, 1H), 0.95 – 0.91 (m, 3H), 0.90 – 0.84 (m, 9H), 0.04(s, 9H); ^13^C NMR (101 MHz, CDCl_3_):δ 136.2, 135.6, 115.1,115.0, 69.6, 69.2, 52.0, 51.2, 47.3, 47.2, 43.7, 42.2, 31.2, 31.0, 30.1, 30.0, 26.0, 25.8, 23.4, 21.9, -1.8, -2.0; IR (neat) cm^-1^ ṽ: 2954, 1626, 1471, 1366, 1249, 1023, 898, 839, 691; HRMS (EI(+), 70 eV) : C_15_H_32_OSi[M-OH]^+^: calcd. 239.2222, found 239.2190; [α]_D_^20^ = + 10.6 (c = 3.50, CH_2_Cl_2_); Enantiomeric excess was determined by HPLC analysis of the 3,5-nitrobenzoate derivative of the product (Chiralcel OD-H column, hexanes:i-PrOH = 99.5:0.5, 0.4 mL/min, 230 nm), t_major_ = 14.9 min, t_minor_ = 18.0 min, 95% de.

**(1R,2S)-1-phenyl-2-(trimethylsilyl)but-3-en-1-ol (2l)**

According to the general procedure with method B, **2l** (24.7 mg, 0.112 mmol) was prepared from benzaldehyde(21.2 mg, 0.20 mmol) as yellow oil in 56% yield.

^1^H NMR (400 MHz, CDCl_3_) δ 7.36 – 7.24 (m, 5H), 5.86 (dt, *J* = 17.1, 10.3 Hz, 1H), 5.11 (dd, *J* = 10.3, 1.9 Hz, 1H), 5.03 (ddd, *J* = 17.1, 1.8, 0.7 Hz, 1H), 4.80 (d, *J* = 8.4 Hz, 1H), 2.22 (d, *J* = 1.6 Hz 1H), 2.08 (dd, *J* = 10.2, 8.6 Hz, 1H), -0.20 (s, 9H); ^13^C NMR (101 MHz, CDCl_3_):δ 143.6, 136.6, 128.4, 127.8, 126.9, 116.0,74.5, 45.6, -2.4; IR (neat) cm^-1^ ṽ: 3458, 2952, 1626, 1248, 907,764, 699;HRMS (EI(+), 70 eV) : C_13_H_20_OSi[M-OH]^+^: calcd. 203.1283., found 203.1249; [α]_D_^20^ = -16.3 (c = 1.00, CH_2_Cl_2_); HPLC (Chiralcel OD-H column,hexanes:i-PrOH = 95:5, 1 mL/min, 210 nm), t_major_ = 7.0 min, t_minor_ = 12.1 min, 93% ee. The reported value^[7]^ for the (1S,2R)-enantiomer (95% ee) is [α]_D_^25^ = + 47.0 (c= 1.0; CHCl_3_).

**(1R,2S)-1-(4-bromophenyl)-2-(trimethylsilyl)but-3-en-1-ol (2m)**

According to the general procedure with method B, **2m** (33.4 mg, 0.112 mmol) was prepared from 4-bromobenzaldehyde (37.0 mg, 0.20 mmol) as yellow oil in 56% yield.

^1^H NMR (400 MHz, CDCl_3_): δ 7.45 (d, *J* = 8.2 Hz, 2H), 7.21 (d, *J* = 8.4 Hz, 2H), 5.82 (dt, *J* = 17.1, 10.3 Hz, 1H), 5.09 (dd, *J* = 10.3, 1.7 Hz, 1H), 4.98 (ddd, *J* = 17.1, 1.7 Hz, 0.8Hz,1H), 4.77 (d, *J* = 8.0 Hz, 1H), 2.23 (br, 1H), 1.99 (dd, *J* = 10.3, 8.1 Hz, 1H), -0.16 (s, 9H); ^13^C NMR (101 MHz, CDCl_3_):δ 142.8, 135.9, 131.4, 128.5, 121.4, 116.3,73.8, 45.5, -2.4; IR (neat) cm^-1^ ṽ: 3435, 2957,1626, 1486, 1409, 1250, 1088,1009, 909, 837, 693.08; HRMS (EI(+), 70 eV) : C_13_H_19_BrOSi [M-OH]^+^: calcd. 281.0389, found 281.0356; [α]_D_^20^ = -19.6 (c = 1.10, CH_2_Cl_2_); HPLC (Chiralcel OD-H column, hexanes:i-PrOH = 95;5, 1 mL/min, 210 nm), t_minor_ = 6.2 min, t_major_ = 6.9 min, 92% ee. The reported value^[7]^ for the (1S,2R)-enantiomer (94% ee) is [α]_D_^25^ = + 10.3 (c= 1.0; CHCl_3_).

**(1R,2S)-1-(2-fluorophenyl)-2-(trimethylsilyl)but-3-en-1-ol (2n)**

According to the general procedure with method B, **2n** (29.5 mg, 0.124 mmol) was prepared from 2-fluorobenzaldehyde(24.8 mg, 0.20 mmol) as yellow oil in 62% yield.

^1^H NMR (400 MHz, CDCl_3_): δ 7.45 – 7.39 (m, 1H), 7.25 – 7.20 (m, 1H), 7.16 – 7.10 (m, 1H), 7.04 – 6.97 (m, 1H), 5.85 (dt, *J* = 17.2, 10.4 Hz, 1H), 5.17 (dd, *J* = 7.9, 2.9 Hz, 1H), 5.08 (dd, *J* = 10.3, 1.9 Hz, 1H), 4.98 (dd, *J* = 17.0, 1.8 Hz, 1H), 2.18 – 2.10 (m, 2H), -0.12 (s, 9H).

^13^C NMR (101 MHz, CDCl_3_): δ159.8 (d, *J_C-F_*= 246.4 Hz), 136.0, 130.9 (d, *J_C-F_* = 13.0 Hz), 128.9 (d, *J_C-F_* = 8.4 Hz), 128.4 (d, *J_C-F_* = 4.5 Hz), 124.0 (d, *J_C-F_* = 3.4 Hz), 116.0, 115.3 (d, *J_C-F_* = 22.2 Hz), 68.0, 44.2, -2.5. IR (neat) cm^-1^ ṽ: 3359, 2925,1626, 1487, 1456,1249, 1055, 911, 841, 758, 693;HRMS (EI(+), 70 eV) : C_13_H_19_FOSi [M-OH]^+^: calcd:221.1189, found:221.1157; [α]_D_^20^ = -17.5 (c = 1.00, CH_2_Cl_2_). HPLC (Chiralcel OD-H column, hexanes:i-PrOH = 95:5, 0.4 mL/min, 210 nm), t_major_ = 13.4 min, t_minor_ = 14.2 min, 90% ee.

**(1R,2S)-1-(3-fluorophenyl)-2-(trimethylsilyl)but-3-en-1-ol (2o)**

According to the general procedure with method B, **2o** (27.6 mg, 0.116 mmol) was prepared from 3-fluorobenzaldehyde (24.8 mg, 0.20 mmol) as yellow oil in 58% yield.

^1^H NMR (400 MHz, CDCl_3_): δ 7.33 – 7.24 (m, 1H), 7.13 – 7.03 (m, 2H), 6.96 (td, *J* = 8.4, 1.8 Hz, 1H), 5.82 (dt, *J* = 17.1, 10.3 Hz, 1H), 5.10 (dd, *J* = 10.3, 1.7 Hz, 1H), 4.99 (dd, *J* = 17.1, 1.1 Hz, 1H), 4.81 (d, *J* = 7.9 Hz, 1H), 2.26 (br, 1H), 2.01 (dd, *J* = 10.2, 8.0 Hz, 1H), -0.15 (s, 9H). ^13^C NMR (101 MHz, CDCl_3_):δ 162.9 (d, *J_C-F_*= 247.4 Hz), 146.4 (d, *J* = 6.7 Hz), 135.9, 129.8 (d, *J_C-F_*= 8.2 Hz), 122.4 (d, *J_C-F_* = 2.8 Hz), 116.3, 114.6 (d, *J_C-F_* = 21.2 Hz), 113.6 (d,*J_C-F_* = 22.2 Hz), 73.9, 45.5, -2.4; IR (neat) cm^-1^ ṽ: 2956, 2927, 1669, 1592, 1451,1257, 1090, 1029, 841, 801, 696; HRMS (EI(+), 70 eV) : C_13_H_19_FOSi[M-H]^+^: calcd. 237.1189, found 237.1105; [α]_D_^20^ = -15.2 (c = 0.70, CH_2_Cl_2_); HPLC (Chiralcel OD-H column, hexanes:i-PrOH = 95:5, 1.0 mL/min, 210 nm), t_major_ = 6.4 min, t_minor_ = 12.4 min, 92% ee.

**(1R,2S)-1-(4-chlorophenyl)-2-(trimethylsilyl)but-3-en-1-ol (2p)**

According to the general procedure with method B, **2p** (32.5 mg, 0.128 mmol) was prepared from 4-chlorobenzaldehyde (28.1 mg, 0.20 mmol) as yellow oil in 64% yield.

^1^H NMR (400 MHz, CDCl_3_): δ 7.34 – 7.22 (m, 4H), 5.83 (dt, *J* = 17.1, 10.2 Hz, 1H), 5.10 (d, *J* = 10.2 Hz, 1H), 4.99 (d, *J* = 17.2 Hz, 1H), 4.79 (d, *J* = 8.0 Hz, 1H), 2.22 (br, 1H), 2.00 (dd, *J* = 10.1, 8.3 Hz, 1H), -0.16 (s, 9H).^13^C NMR (101 MHz, CDCl_3_):δ 142.2, 136.0, 133.3, 128.5, 128.2, 116.3,73.8, 45.6, -2.4; IR (neat) cm^-1^ ṽ: 3714, 3075, 2953,1625, 1491, 1410, 1248, 1010, 911, 836, 694; HRMS (EI(+), 70 eV) : C_13_H_19_ClOSi [M-OH]^+^calcd:237.0894, found:237.0861;[α]_D_^20^ = -12.3 (c = 2.00, CH_2_Cl_2_); HPLC (Chiralcel OD-H column, hexanes:i-PrOH = 95:5, 0.4 mL/min, 210 nm), t_minor_ = 14.7 min, t_major_ = 15.5 min, 91% ee.

The reported value^[7]^ for the (1S,2R)-enantiomer (59% ee) is [α]_D_^25^ = + 4.8 (c= 1.0; CHCl_3_).

**(1R,2S)-1-(3-(trifluoromethyl)phenyl)-2-(trimethylsilyl)but-3-en-1-ol (2q)**

According to the general procedure with method B, **2q** (39.2 mg, 0.136 mmol) was prepared from 3-(trifluoromethyl)benzaldehyde (34.8 mg, 0.20 mmol) as yellow oil in 68% yield.

^1^H NMR (400 MHz, CDCl_3_): δ 7.59 (s, 1H), 7.55–7.48 (m, 2H), 7.47– 7.41 (m, 1H), 5.83 (dt, *J* = 17.1, 10.3 Hz, 1H), 5.10 (dd, *J* = 10.2, 1.7 Hz, 1H), 4.98 (dd, *J* = 17.1, 1.2 Hz, 1H), 4.86 (d, *J*= 7.8 Hz, 1H), 2.40 (br, 1H), 2.02 (dd, *J* = 10.3, 7.8 Hz, 1H), -0.15 (s, 9H).

^13^C NMR (101 MHz, CDCl_3_): δ 144.8, 135.6, 130.6 (q, *J_C-F_*=32.3 Hz), 130.1, 128.7, 124.4 (q, *J_C-F_* = 4.0 Hz), 124.1 (q, *J_C-F_*=273.7 Hz), 123.6 (q, *J_C-F_* = 4.0 Hz), 116.6, 73.9, 45.6, -2.5.

; IR (neat) cm^-1^ ṽ: 3442, 3077, 2955,1672, 1626,1328, 1250, 1165, 1127, 1072, 901, 839, 701;HRMS (EI(+), 70 eV) : C_14_H_19_F_3_OSi [M-OH]^+^: calcd. 271.1157, found 271.1124; [α]_D_^20^ = -15.8 (c = 0.80, CH_2_Cl_2_); HPLC (Chiralcel OD-H column, hexanes:i-PrOH = 90:10, 1.0 mL/min, 210 nm), t_major_ = 3.8 min, t_minor_ = 4.3 min, 91% ee.

**(1R,2S)-1-(m-tolyl)-2-(trimethylsilyl)but-3-en-1-ol (2r)**

According to the general procedure with method B, **2r** (28.6 mg, 0.122 mmol) was prepared from 4-methyl benzaldehyde (24.0 mg, 0.20 mmol) as yellow oil in 61% yield.

^1^H NMR (400 MHz, CDCl_3_): δ 7.21 – 6.95 (m, 4H), 5.77 (dt, *J* = 17.1, 10.3 Hz, 1H), 5.03 (d, *J* = 10.3 Hz, 1H), 4.94 (d, *J* = 17.1 Hz, 1H), 4.67 (d, *J*= 8.6 Hz, 1H), 2.27 (s, 3H), 2.16 (br, 1H), 2.00 (dd,*J* = 10.3, 7.9 Hz, 1H), -0.27 (s, 9H).^13^C NMR (101 MHz, CDCl_3_): δ 143.5, 137.9, 136.7, 128.5, 128.2, 127.5, 124.0, 115.9, 74.5, 45.5, 21.4, -2.4;IR (neat) cm^-1^ ṽ: 3447, 2961, 626, 1411, 1256,1087, 1011, 838, 790, 701; HRMS (EI(+), 70 eV) : C_14_H_22_OSi [M-H]^+^: calcd. 233.1440, found 233.1357; [α]_D_^20^ = -16.5 (c = 1.50, CH_2_Cl_2_); HPLC (Chiralcel OD-H column, hexanes:i-PrOH = 90:10, 1 mL/min, 210 nm), t_major_ = 4.3 min, t_minor_ = 4.9 min, 92% ee. The reported value^[7]^ for the (1S,2R)-enantiomer (58% ee) is [α]_D_^25^ = + 16.7 (c= 1.0; CHCl_3_).

**(1R,2S)-1-(p-tolyl)-2-(trimethylsilyl)but-3-en-1-ol (2s)**

According to the general procedure with method B, **2s** (24.8 mg, 0.106 mmol) was prepared from 4-methyl benzaldehyde (24.0 mg, 0.20mmol) as yellow oil in 53% yield.

^1^H NMR (400 MHz, CDCl_3_): δ 7.22 (d, *J* = 8.0 Hz, 2H), 7.13 (d, *J* = 8.0 Hz, 2H), 5.86 (dt, *J* = 17.2, 10.3 Hz, 1H), 5.10 (dd, *J* = 10.3, 1.7 Hz, 1H), 5.02 (ddd, *J* = 17.1, 1.7, 0.8 Hz,1H), 4.76 (d, *J* = 8.5 Hz, 1H), 2.34 (br, 3H), 2.18 (s, 1H), 2.07 (dd, *J* = 10.3, 7.9 Hz,1H), -0.19 (s, 9H). ^13^C NMR (101 MHz, CDCl_3_):δ 140.6, 137.4, 136.8, 129.0, 126.7, 115.8, 74.3, 45.5, 21.15, -2.4; IR (neat) cm^-1^ ṽ: 3432, 2953, 1625, 1513, 1247, 998, 909, 838, 692, 636; HRMS (EI(+), 70 eV) : C_14_H_22_OSi [M]^+^: calcd. 217.1440, found 217.1407; [α]_D_^20^ = -25.1 (c = 0.70, CH_2_Cl_2_); HPLC (Chiralcel OD-H column, hexanes:i-PrOH = 99:1, 0.4 mL/min, 210 nm), t_major_ = 28.0 min, t_minor_ = 29.3 min, 91% ee.

**(1S,2S)-1-(thiophen-2-yl)-2-(trimethylsilyl)but-3-en-1-ol (2t)**

According to the general procedure with method B, **2t** (29.8 mg, 0.132 mmol) was prepared from 2-thenaldehyde(22.4 mg, 0.20 mmol) as yellow oil in 66% yield.

^1^H NMR (400 MHz, CDCl_3_): δ 7.25 – 7.19 (m, 1H), 6.98 – 6.94 (m, 1H), 6.91 (dd, *J* = 5.0, 3.5 Hz, 1H), 5.83 (dt, *J* = 17.1, 10.3 Hz, 1H), 5.15 – 4.99 (m, 3H), 2.35 (br, 1H), 2.09 (dd, *J* = 10.1, 8.4 Hz, 1H), -0.14 (s, 9H).^13^C NMR (101 MHz, CDCl_3_):δ 147.9, 136.1, 126.2, 124.8, 124.4, 116.2, 70.0, 46.2, -2.6; IR (neat) cm^-1^ ṽ: 3430, 2925, 1712, 1662, 1412, 1242, 1043, 855, 704; HRMS (EI(+), 70 eV) : C_11_H_18_OSSi[M-H]^+^: calcd. 225.0848, found 225.0764; [α]_D_^20^ = -7.1 (c = 0.60, CH_2_Cl_2_); HPLC (Chiralcel OD-H column, hexanes:i-PrOH = 95:5, 1 mL/min, 210 nm), t_major_ = 7.0 min, t_minor_ = 9.8 min, 92% ee.

**(3R,4S,E)-1-phenyl-4-(trimethylsilyl)hexa-1,5-dien-3-ol (2u)**

According to the general procedure with method B, **2u** (41.4 mg, 0.168 mmol) was prepared from cinnamaldehyde(26.4 mg, 0.20 mmol) as yellow oil in 84% yield.

^1^H NMR (400 MHz, CDCl_3_): δ 7.40 – 7.36 (m, 2H), 7.35 – 7.29 (m, 2H), 7.27 – 7.22 (m, 1H), 6.58 (d, *J* = 15.8 Hz, 1H), 6.21 (dd, *J* = 15.8, 7.8 Hz, 1H), 5.83 (dt, *J* = 17.1, 10.3 Hz, 1H), 5.11 (dd, *J* = 10.2, 1.9 Hz, 1H), 5.02 (dd, *J* = 17.1, 1.8 Hz, 1H), 4.43 (dd, *J* = 8.0, 7.8 Hz, 1H), 1.92 – 1.85 (m, 2H), 0.05 (s, 9H). ^13^C NMR (101 MHz, CDCl_3_): δ 136.6, 136.1, 131.9, 130.8, 128.6, 127.7, 126.5, 115.7, 73.2, 44.1, -1.9; IR (neat) cm^-1^ ṽ: 3359, 2923, 2853, 1660, 1462, 1258, 1091, 1031,840, 802, 696; HRMS (EI(+), 70 eV) : C_15_H_22_OSi[M-OH]^+^: calcd. 229.1440, found: 229.1407; [α]_D_^20^ = + 26.8 (c = 1.50, CH_2_Cl_2_); HPLC (Chiralcel OD-H column, hexanes:i-PrOH = 95:5, 1 mL/min, 210 nm), t_minor_ = 7.2 min, t_major_ = 12.4 min, 98% ee.

**(3R,4S,E)-1-(4-fluorophenyl)-4-(trimethylsilyl)hexa-1,5-dien-3-ol (2v)**

According to the general procedure with method B, **2v** (38.0 mg, 0.144 mmol) was prepared from 4-fluorocinnamaldehyde (30.0 mg, 0.20 mmol) as yellow oil in 72% yield.

^1^H NMR (400 MHz, CDCl_3_): δ 7.34 (dd, *J* = 8.4, 5.5 Hz, 2H), 7.00 (t, *J* = 8.6 Hz, 2H), 6.54 (d, *J* = 15.9 Hz, 1H), 6.12 (dd, *J* = 15.8, 7.7 Hz, 1H), 5.83 (dt, *J* = 17.1, 10.3 Hz, 1H), 5.11 (d, *J* = 10.3 Hz, 1H), 5.02 (d, *J* = 17.1 Hz, 1H), 4.41 (t, *J* = 7.6 Hz, 1H), 1.91 (br, 1H), 1.86 (dd, *J* = 10.4, 7.7 Hz, 1H), 0.04 (s, 9H); ^13^C NMR (101 MHz, cdcl_3_): δ 162.3 (d, *J*_C-F_= 247.4Hz), 136.0, 132.8 (d, *J*_C-F_= 3.4 Hz), 131.6 (d, *J*_C-F_ = 2.3 Hz), 129.6, 128.0 (d,2C, *J*_C-F_ = 8.0 Hz), 115.8, 115.5 (d, 2C, *J*_C-F_ = 22.2 Hz), 77.3, 77.0, 76.7, 73.1, 44.1, -1.9; IR (neat) cm^-1^ ṽ: 3367, 2925, 2855,1724, 1664, 1601, 1510, 1229, 1158, 1091, 837, 698; HRMS (EI(+), 70 eV) : C_15_H_21_FOSi [M-H]^+^: calcd. 263.1346, found 263.1263; [α]_D_^20^ = + 13.9 (c = 0.30, CH_2_Cl_2_); HPLC (Chiralcel OD-H column, hexanes:i-PrOH = 99:1, 1.0 mL/min, 210 nm), t_minor_ = 9.4 min, t_major_ = 10.1 min, 97% ee.

**(3R,4S)-1-phenyl-4-(trifluoromethyl)hex-5-en-3-ol (3a)**

According to the general procedure with method C, **3a** (46.4 mg, 0.190 mmol) was prepared from Phenylpropyl aldehyde (26.8 mg, 0.20 mmol) as yellow oil in 95% yield.

^1^H NMR (400 MHz, CDCl_3_) δ 7.31 –7.27 (m, 2H), 7.23 – 7.18 (m, 3H), 5.87 (dt, *J* = 17.2, 10.0 Hz, 1H), 5.45 (d, *J* = 10.0 Hz, 1H), 5.32 (d, *J* = 17.2 Hz, 1H), 4.10 – 4.02 (m, 1H), 2.86 – 2.75 (m, 1H), 2.75 – 2.55 (m, 2H), 1.90 – 1.81 (m, 1H), 1.78 – 1.65 (m, 1H); ^19^F NMR (376 MHz, CDCl_3_) δ -67.60 (d, *J* = 9.4 Hz); ^13^C NMR (100 MHz, CDCl_3_) δ 141.2, 128.5,128.4, 127.4 (q, *J* = 2.5 Hz), 126.3 (q, *J* = 279.2 Hz), 126.1, 123.5, 68.1 (q, *J* = 2.2 Hz), 53.7 (q, *J* = 24.4 Hz), 36.5, 31.9; HRMS (EI(+), 70 eV) : C_13_H_15_F_3_O [M]^+^: calcd. 244.1075, found 244.1086; IR (neat) cm^-1^ ṽ: 3461, 3079, 3030, 2956, 2863, 1603, 1498, 1335, 1259, 1153, 1100, 1022, 938, 801,748,706; [α]_D_^20^ = + 34.5 (c = 3.11, CH_2_Cl_2_); HPLC (Chiralcel OD-H column,hexanes:i-PrOH = 90:10, 1.0 mL/min, 210 nm), t_minor_ = 5.5 min, t_major_ = 5.9 min, 91% ee. The reported value^[8]^ for the (3S,4R)-enantiomer (94% ee) is [α]_D_^25^ = - 15.01 (c= 0.4; CH_2_Cl_2_).

**(3S,4R)-3-(trifluoromethyl)non-1-en-4-ol (3b)**

According to the general procedure with method C, **3b** (28.2 mg, 0.134 mmol) was prepared from Hexaldehyde (20.0 mg, 0.20 mmol) as yellow oil in 67% yield.

^1^H NMR (400 MHz, CDCl_3_) δ 5.86 (dt, *J* = 17.2, 10.0 Hz, 1H), 5.45 (d, *J* = 10.0 Hz, 1H), 5.33 (d, *J* = 17.2 Hz, 1H), 4.13 – 3.94 (m, 1H), 2.76 – 2.66 (m , 1H), 1.82 (br, 1H), 1.63 – 1.17 (m, 8H), 0.89 (t, *J* = 6.8 Hz, 3H); ^19^F NMR (376 MHz, CDCl_3_) δ -67.72 (d, *J* = 9.4 Hz); ^13^C NMR (100 MHz, CDCl_3_) δ 127.4 (q, *J* = 2.5 Hz), 126.5 (q, *J* = 279.3 Hz), 123.3, 68.7 (q, *J* = 2.3 Hz), 53.4 (q, *J* = 24.3 Hz), 34.8, 31.5, 25.2, 22.5, 14.0; HRMS (EI(+), 70 eV) : C_10_H_17_F_3_O [M-H2O]^+^: calcd. 192.1126, found 192.1125; IR (neat) cm^-1^ ṽ:2956, 2925, 2859, 1726, 1645, 1460, 1262, 1097, 1024, 803, 694; [α]_D_^20^ = + 16.1 (c = 1.50, CH_2_Cl_2_); Enantiomeric excess was determined by HPLC analysis of the 3,5-nitrobenzoatederivative of the product (Chiralcel OD-H column, hexanes:i-PrOH = 90:10, 1.0 mL/min, 210 nm), t_major_ = 6.1min, t_minor_ = 7.6 min, 93% ee.

**(3S,4R)-8-chloro-3-(trifluoromethyl)oct-1-en-4-ol (3c)**

According to the general procedure with method C, **3c** (37.8 mg, 0.164 mmol) was prepared from 5-chloropentanal (24.0 mg, 0.20 mmol) as yellow oil in 82% yield.

^1^H NMR (400 MHz, CDCl_3_) δ 5.86 (dt, *J* = 17.2, 10.0 Hz, 1H), 5.46 (d, *J* = 10.0 Hz, 1H), 5.35 (d, *J* = 17.2 Hz, 1H), 4.12 – 3.93 (m, 1H), 3.54 (t, *J* = 6.8 Hz, 2H), 2.78 – 2.64 (m, 1H), 1.85 – 1.75 (m, 2H), 1.68 – 1.39 (m, 4H); ^19^F NMR (376 MHz, CDCl_3_) δ -67.68 (d, *J* = 9.4 Hz); ^13^C NMR (100 MHz, CDCl_3_) δ 127.2 (q, *J* = 2.5 Hz), 126.3(q, *J* = 279.3 Hz), 123.6, 68.4(q, *J* = 2.2 Hz), 53.5(q, *J* = 24.4 Hz), 44.8, 33.9, 32.2, 22.9; HRMS (EI(+), 70 eV) : C_9_H_14_ClF_3_O [M-H2O]^+^: calcd. 212.0580, found 212.0589; IR (neat) cm^-1^ ṽ: 3447, 2956, 2866, 1642, 1337, 1259, 1147, 1010, 1023, 939, 802, 719, 653; [α]_D_^20^ = + 32.0 (c = 1.34, CH_2_Cl_2_); Enantiomeric excess was determined by HPLC analysis of the 3,5-nitrobenzoatederivative of the product (Chiralcel OD-H column, hexanes:i-PrOH = 90:10, 1.0 mL/min, 210 nm), t_major_ = 11.3min, t_minor_ = 17.4 min, 95% ee.

**(3S,4R)-6,8,8-trimethyl-3-(trifluoromethyl)non-1-en-4-ol (3d)**

According to the general procedure with method C, **3d** (39.0 mg, 0.155 mmol) was prepared from 3,5,5-trimethylhexanal (28.4 mg, 0.20 mmol) as yellow oil in 77% yield.

^1^H NMR (400 MHz, CDCl_3_) δ 5.96 – 5.75 (m, 1H), 5.46 (d, *J* = 10.4 Hz, 1H), 5.38 – 5.29 (m, 1H),4.20 -4.05 (m, 1H), 2.81 – 2.54 (m, 1H), 1.75 – 1.40 (m, 2H), 1.43 – 1.28 (m, 1H), 1.23 – 1.06 (m, 2H), 0.98 – 0.93 (m, 3H), 0.90 (s, 9H); ^19^F NMR (376 MHz, CDCl_3_) δ -67.64 (d, *J* =9.4 Hz); -67.73 (d, *J* =9.4 Hz); ^13^C NMR (100 MHz, CDCl_3_) δ127.5 (q, *J* = 2.1 Hz), 126.4 (q, *J* = 279.2 Hz), 123.4, 66.5 (q, *J* = 2.3 Hz), 54.1 (q, *J* = 24.2 Hz), 51.6, 44.5, 31.2, 30.0, 25.3, 21.8;127.2 (q, *J* = 2.1 Hz),126.5 (q, *J* = 279.2 Hz), 123.5, 66.8 (q, *J* = 2.0 Hz), 53.2 (q, *J* = 24.2 Hz), 50.9, 44.4, 31.0, 30.0, 25.6, 23.2; HRMS (EI(+), 70 eV) : C_13_H_23_F_3_O [M-CH_3_]^+^: calcd. 237.1466, found 237.1471; IR (neat) cm^-1^ ṽ: 3394, 2958, 2923, 2858, 1644, 1372, 1261, 1092, 1023, 866, 802, 695; [α]_D_^20^ = + 62.9 (c = 0.12, CH_2_Cl_2_); Enantiomeric excess was determined by HPLC analysis of the 3,5-nitrobenzoatederivative of the product (Chiralcel OD-H column, hexanes:i-PrOH = 95:5, 1.3mL/min, 230 nm), t_minor_ = 8.2min, t_major_ = 9.0 min, 98% de.

**(2S,3R)-1-(2,2-dimethyl-1,3-dioxolan-4-yl)-3-(trifluoromethyl)pent-4-en-2-ol (3e)**

According to the general procedure with method C, **3e** (35.6 mg, 0.140 mmol) was prepared from 2-(2,2-dimethyl-1,3-dioxolan-4-yl)acetaldehyde^[11]^ (28.8 mg, 0.20 mmol) as yellow oil in 70% yield.

^1^H NMR (400 MHz, CDCl_3_) δ 6.00 – 5.80 (m, 1H), 5.49 – 5.41 (m, 1H), 5.38 – 5.28 (m, 1H),4.40 – 4.25 (m, 2H), 4.13 – 4.04 (m, 1H), 3.61 – 3.53 (m, 1H), 2.84 – 2.61 (m, 1H), 1.84 – 1.67 (m, 1H), 1.65 – 1.55 (m, 1H), 1.43 – 1.39 (m, 3H), 1.35 (s, 3H); ^19^F NMR (376 MHz, CDCl_3_) δ -67.64 (d, *J* = 9.4 Hz); -67.69 (d, *J* = 9.4 Hz); ^13^C NMR (100 MHz, CDCl_3_) δ 127.7 (q, *J* = 2.4 Hz), 126.0 (q, *J* = 279.2 Hz), 123.1, 109.7, 75.3, 69.5, 68.0 (q, *J* = 2.4 Hz), 54.0 (q,*J* = 24.8 Hz), 38.1, 26.9, 25.6; 127.3 (q, *J* = 2.4 Hz), 126.2 (q, *J* = 279.2 Hz), 123.6, 109.0, 73.0, 69.3, 65.9 (q, *J* = 2.4 Hz), 54.0 (q, *J* = 24.8 Hz), 38.3, 26.8, 25.6. HRMS (EI(+), 70 eV) :C_11_H_17_F_3_O_3_ [M-CH_3_]^+^: calcd. 239.0895, found 239.0894; IR (neat) cm^-1^ ṽ: 2988, 2944, 2883, 1735, 1423, 1377, 1333, 1258, 1160, 1104, 1065, 934, 838, 800, 752, 716. [α]_D_^20^ = + 19.5 (c = 0.29, CH_2_Cl_2_); Enantiomeric excess was determined by HPLC analysis of the 3,5-nitrobenzoatederivative of the product (Chiralcel IC column, hexanes:i-PrOH = 95:5, 1.3mL/min, 230 nm), t_minor_ = 10.6 min, t_major_ = 11.1 min, 98% de.

**(3S,4R,6S)-6,10-dimethyl-3-(trifluoromethyl)undeca-1,9-dien-4-ol (3f)**

According to the general procedure with method C, **3f** (37.0 mg, 0.140 mmol) was prepared from citronellal(30.9 mg, 0.20 mmol) as yellow oil in 70% yield.

^1^H NMR (400 MHz, CDCl_3_) δ 5.87 (dt, *J* = 17.2, 10.0 Hz, 1H), 5.46 (d, *J* = 10.0 Hz, 1H), 5.33 (d, *J* = 17.2 Hz, 1H), 5.17 – 5.00 (m, 1H), 4.26 – 4.08 (m, 1H), 2.76 – 2.60 (m, 1H), 2.08 – 1.86 (m, 2H), 1.68 (s, 3H), 1.65 – 1.52 (m, 5H), 1.45 – 1.05 (m, 3H),0.95 – 0.90 (m, 3H); ^19^F NMR (376 MHz, CDCl_3_) δ -67.68 (d, *J* =9.4 Hz), -67.75 (d, *J* =9.4 Hz); ^13^C NMR (100 MHz, CDCl_3_) δ 131.5, 127.4 (q, *J* = 2.6 Hz), 126.4 (q, *J* = 279.3 Hz), 124.5, 123.5, , 66.7 (q, *J* = 2.2 Hz), 54.1 (q, *J* = 24.2 Hz), 42.1, 37.5, 28.8, 25.7(2C, overlap), 25.4, 19.9, 17.64,131.4, 127.2(q, *J* = 2.6 Hz), 126.5 (q, *J* = 279.3 Hz), 124.4, 123.4, 66.4 (q, *J* = 2.2 Hz), 53.2(q, *J* = 24.2 Hz), 42.0, 36.5, 28.4,25.7(2C, overlap), 25.2, 18.9; HRMS (EI(+), 70 eV) : C_14_H_23_F_3_O [M]^+^: calcd. 264.1701, found 264.1699; IR (neat) cm^-1^ ṽ:2962, 2925, 2862,1454, 1381, 1333, 1260,1153, 1095, 1024, 932, 861, 804, 709; [α]_D_^20^ = + 44.9 (c = 0.1, CH_2_Cl_2_); Enantiomeric excess was determined by HPLC analysis of the 3,5-nitrobenzoatederivative of the product (Chiralcel OD-H column, hexanes:i-PrOH = 90:10, 1.0 mL/min, 210 nm), t_major_ = 5.5min, t_minor_ = 6.2 min, 96% ee.

**(2S,3S)-1-(benzyloxy)-3-(trifluoromethyl)pent-4-en-2-ol (3g)**

According to the general procedure with method C, **3g** (36.4 mg, 0.14 mmol) was prepared from 2-(benzyloxy)acetaldehyde (38.0 mg, 0.20 mmol) as yellow oil in 70% yield.

^1^H NMR (400 MHz, CDCl_3_) δ 7.53 – 7.07 (m, 5H), 5.87 (dt, *J* = 17.2, 10.4 Hz, 1H), 5.41 (d, *J* = 10.4 Hz, 1H), 5.29 (d, *J* = 17.2 Hz, 1H), 4.65 – 4.45 (m, 2H), 4.29 – 4.19 (m, 1H), 3.44 (d, *J* = 6.4 Hz, 2H), 2.99 – 2.77 (m, 1H), 2.43 (br, 1H); ^19^F NMR (376 MHz, CDCl_3_) δ -67.83 (d, *J* = 9.4 Hz); ^13^C NMR (100 MHz, CDCl_3_) δ 137.5, 128.5, 127.9, 127.8, 127.2 (q, *J* = 2.4 Hz ), 126.1 (q, *J* = 278.9 Hz ), 123.3, 73.4, 71.4, 67.2 (q, *J* = 2.2 Hz ), 50.4 (q, *J* = 25.6 Hz ); HRMS (EI(+), 70 eV) : C_13_H_15_F_3_O_2_ [M]^+^: calcd. 260.1024, found 260.1028; IR (neat) cm^-1^ ṽ: 2963, 2920, 2868, 1261, 1170, 1101, 1023, 938, 802, 746, 701; [α]_D_^20^ = + 12.4 (c = 2.7, CH_2_Cl_2_); HPLC (Chiralcel IE column, hexanes:i-PrOH = 98:2, 1.0 mL/min, 210 nm), t_minor_ = 6.7 min, t_major_ = 7.0 min, 90% ee. The reported value^[8]^ for the (2R,3R)-enantiomer (99% ee) is [α]_D_^25^ = - 10.67 (c= 1.5; CH_2_Cl_2_).

**(3R,4S,E)-1-phenyl-4-(trifluoromethyl)hexa-1,5-dien-3-ol (3h)**

According to the general procedure with method C, **3h** (21.0 mg, 0.086 mmol) was prepared from cinnamaldehyde (26.4 mg, 0.20mmol) as yellow oil in 43% yield.

^1^H NMR (400 MHz, CDCl_3_) δ 7.38 - 7.25 (m,5H), 6.66 (d, *J* = 16.0 Hz, 1H), 6.18 (dd, *J* = 16.0, 7.2 Hz, 1H), 6.04 – 5.78 (m, 1H), 5.48 (d, *J* = 10.4 Hz, 1H), 5.37 (d, *J* = 17.2 Hz, 1H), 4.76 – 4.68 (m, 1H), 3.00 – 2.84 (m, 1H), 1.91 (br, 1H );^19^F NMR (376 MHz, CDCl_3_) δ -67.29 (d, *J* = 9.0 Hz); ^13^C NMR (100 MHz, CDCl_3_) δ 136.1, 132.3, 128.6, 128.1,127.3 (q, *J* = 2.4 Hz), 126.6, 126.0 (q, *J* = 279.2 Hz),123.6, 70.2 (q, *J* = 2.4 Hz), 54.3 (q, *J* = 24.8 Hz); HRMS (EI(+), 70 eV) : C_13_H_13_F_3_O [M]^+^: calcd. 242.0918, found 242.0919; IR (neat) cm^-1^ ṽ: 3441, 3031, 2926, 2859, 1669, 1594, 1496, 1339, 1258, 1171, 1101, 1023, 972, 936, 865, 802, 756, 694; [α]_D_^20^ = + 18.7 (c = 0.85, CH_2_Cl_2_); HPLC (Chiralcel OD-H column, hexanes:i-PrOH = 90:10, 1.0 mL/min, 210 nm), t_minor_ = 7.8 min, t_major_ = 9.7 min, 93% ee. The reported value^[8]^ for the (3S,4R,E)-enantiomer (87% ee) is [α]_D_^25^ = - 23.64 (c= 1.1; CH_2_Cl_2_).

**(3S,4R,E)-1-(4-methoxyphenyl)-4-(trifluoromethyl)hexa-1,5-dien-3-ol (3i)**

According to the general procedure with method C, **3i** (34.3mg, 0.126mmol) was prepared from 4-methoxycinnamaldehyde(38.0 mg, 0.20mmol) as yellow oil in 63% yield.

^1^H NMR (400 MHz, CDCl_3_) δ 7.32 (d, *J* = 7.6 Hz, 2H), 6.86 (d, *J* = 7.6 Hz, 2H), 6.60 (d, *J* = 15.6 Hz, 1H), 6.05 (dd, *J* = 15.6, 7.2 Hz, 1H), 5.99 – 5.84 (m, 1H), 5.48 (d, *J* = 10.4Hz, 1H), 5.38 (d, *J* = 17.2Hz, 1H), 4.76 – 4.64 (m, 1H), 3.81 (s, 3H), 3.03 – 2.81 (m, 1H), 1.92 (br, 1H);^19^F NMR (376 MHz, CDCl_3_) δ -67.29 (d, *J* = 9.0 Hz);^13^C NMR (100 MHz, CDCl_3_) δ 159.6, 131.9, 128.8, 127.9, 127.4 (q, *J* = 2.7 Hz), 126.0 (q, *J* = 279.3 Hz), 125.8, 123.5, 114.0, 70.4 (q, *J* = 2.5 Hz), 55.3, 54.3 (q, *J* =24.7 Hz); HRMS (EI(+), 70 eV) : C_14_H_15_F_3_O_2_ [M]^+^: calcd. 272.1024, found 272.1029; IR (neat) cm^-1^ ṽ: 3459, 2926, 2844, 1652, 1511, 1458, 1431, 1339, 1302, 1248, 1168, 1099, 1030, 967, 937, 811, 772, 719; [α]_D_^20^ = + 14.7 (c = 1.39, CH_2_Cl_2_); HPLC (Chiralcel OD-H column,hexanes:i-PrOH = 98:2, 1.0 mL/min, 210 nm), t_minor_ = 26.5 min, t_major_ = 28.2 min, 95% ee.

**(3S,4R,E)-1-(4-fluorophenyl)-4-(trifluoromethyl)hexa-1,5-dien-3-ol (3j)**

According to the general procedure with method C, **3j** (20.82 mg, 0.080 mmol) was prepared from 4-fluorocinnamaldehyde (38.0 mg, 0.20 mmol) as yellow oil in 40% yield.

^1^H NMR (400 MHz, CDCl_3_) δ 7.38 – 7.30 (m, 2H), 7.01 (m, 2H), 6.63 (d, *J* = 16.0 Hz, 1H), 6.10 (dd, *J* = 16.0, 6.8 Hz, 1H), 5.91 (dt, *J* = 17.2, 10.4 Hz, 1H), 5.48 (d, *J* = 10.4 Hz, 1H), 5.38 (d, *J* = 17.2 Hz, 1H), 4.73 – 4.66 (m, 1H), 2.99 – 2.84 (m, 1H);^19^F NMR (376 MHz, CDCl_3_) δ -67.25 (d, *J* = 9.4 Hz), -113.56 – -113.70 (m);^13^C NMR (100 MHz, CDCl_3_) δ 162.6 (d, *J* = 246.3 Hz), 132.3 (d, *J* =3.3 Hz), 131.2, 128.2 (d, *J* =8.0 Hz), 127.9, 127.3(q, *J* =2.4 Hz), 126.0 (q, *J* =279.3 Hz), 123.7, 115.6 (d, *J* =21.5 Hz), 70.1 (q, *J* =2.3 Hz),

54.3 (q, *J* =24.8 Hz); HRMS (EI(+), 70 eV) : C_13_H_12_F_4_O [M]^+^: calcd. 260.0824, found 260.0831; IR (neat) cm^-1^ ṽ: 3431, 2963, 1604, 1510, 1413, 1338, 1260, 1092, 1022, 862, 801, 693; [α]_D_^20^ = + 21.4 (c = 1.08, CH_2_Cl_2_); HPLC (Chiralcel OD-H column, hexanes:i-PrOH = 97:3, 1.0 mL/min, 210 nm), t_major_ = 11.8min, t_minor_ = 12.4 min, 93% ee.

**(3R,4S,E)-1-(2-methoxyphenyl)-4-(trifluoromethyl)hexa-1,5-dien-3-ol (3k)**

According to the general procedure with method C, **3k** (25.6 mg, 0.094 mmol) wasprepared from 2-methoxycinnamaldehyde (38.0 mg, 0.20 mmol) as yellow oil in 47% yield.

^1^H NMR (400 MHz, CDCl_3_) δ 7.41 (dd, *J* = 7.6, 1.2 Hz, 1H), 7.29 – 7.22 (m, 1H), 7.02 – 6.84 (m, 3H), 6.21 (dd, *J* = 16.0, 7.2 Hz, 1H), 5.93 (dt, *J* = 17.2, 10.4 Hz,1H), 5.48 (d, *J* = 10.4 Hz, 1H), 5.38 (d, *J* = 17.2 Hz, 1H), 4.77 – 4.66 (m, 1H), 3.85 (s, 3H), 2.99 – 2.87 (m, 1H), 1.95 (br, 1H);^19^F NMR (376 MHz, CDCl_3_) δ -67.29 (d, *J* = 9.4 Hz); ^13^C NMR (100 MHz, CDCl_3_) δ 156.8, 129.2, 128.7, 127.4 (q, *J* = 2.6 Hz), 127.3, 127.0, 126.0 (q, *J* = 279.5 Hz ), 125.0, 123.4, 120.6, 110.8, 70.7 (q, *J* = 2.5 Hz), 55.4, 54.2 (q, *J* = 24.6 Hz); HRMS (EI(+), 70 eV) : C_14_H_15_F_3_O_2_ [M]^+^: calcd. 272.1024, found 272.1032; IR (neat) cm^-1^ ṽ: 3452, 2933, 2845, 1650, 1595, 1480, 1459, 1338, 1249, 1168, 1103, 1025, 975, 937, 866, 751; [α]_D_^20^ = + 24.5 (c = 1.76, CH_2_Cl_2_); HPLC (Chiralcel OD-H column, hexanes:i-PrOH = 90:10, 1.0 mL/min, 210 nm), t_minor_ = 4.8 min, t_major_ = 5.3 min, 93% ee.

**(3S,4R)-3-(difluoromethyl)non-1-en-4-ol (3l)**

According to the general procedure with method D, **3l** (28.4 mg, 0.148 mmol) was prepared from Hexaldehyde (20.0 mg, 0.20 mmol) as yellow oil in 74% yield.

^1^H NMR (400 MHz, CDCl_3_) 6.10 – 5.74 (m, 2H), 5.39 (d, *J* = 10.4 Hz, 1H), 5.28 (d, *J* = 17.2 Hz, 1H), 4.00 – 3.88 (m, 1H), 2.60 – 2.35 (m, 1H), 1.62 – 1.55 (m, 1H), 1.48 – 1.40 (m, 2H), 1.36 – 1.24 (m, 5H), 0.89 (t, *J* = 6.0 Hz, 3H);^19^F NMR (376 MHz, CDCl_3_) δ -121.74 - -122.08 (m, 2F);^13^C NMR (100 MHz, CDCl_3_) δ 129.2 (t, *J* = 5.1 Hz), 122.1, 117.0 (t, *J* = 241.2 Hz), 69.3 (t, *J* = 4.8 Hz), 53.3 (t, *J* = 19.0 Hz), 34.9, 31.6, 25.2, 22.6, 14.0; HRMS (EI(+), 70 eV) : C_10_H_18_F_2_O [M-H2O]^+^: calcd. 174.1220, found 174.1225; IR (neat) cm^-1^ ṽ: 2963, 2921, 2860,1456, 1411, 1297, 1093, 1024, 864, 803, 697; [α]_D_^20^ = + 9.1 (c = 0.21, CH_2_Cl_2_); Enantiomeric excess was determined by HPLC analysis of the 3,5-nitrobenzoatederivative of the product (Chiralcel IC column, CO_2_:i-PrOH = 95:5, 1.3mL/min, 214 nm), t_major_ = 15.1 min, t_minor_ = 17.9 min, 96% ee.

**(3S,4R)-8-chloro-3-(difluoromethyl)oct-1-en-4-ol (3m)**

According to the general procedure with method D, **3m** (32.3 mg, 0.152 mmol) was prepared from 5-chloropentanal (24.0 mg, 0.20 mmol) as yellow oil in 76% yield.

^1^H NMR (400 MHz, CDCl_3_) δ 6.07 – 5.72 (m, 2H), 5.40 (d, *J* = 10.4 Hz, 1H), 5.29 (d, *J* = 17.2 Hz, 1H), 4.02 – 3.88 (m, 1H), 3.54 (t, *J* = 6.4 Hz, 2H), 2.55 – 2.41 (m, 1H), 1.87 – 1.73 (m, 2H), 1.65 – 1.48 (m, 4H); ^19^F NMR (376 MHz, CDCl_3_) δ -121.79 (d, *J* = 13.9 Hz), -121.94 (d, *J* = 13.9 Hz); ^13^C NMR (100 MHz, CDCl_3_) δ 129.0 (t, *J* = 5.0 Hz), 122.3, 116.9 (t, *J* = 241.2 Hz), 69.0 (t, *J* = 4.7 Hz), 53.4 (t, *J* = 19.0 Hz), 44.8, 34.1, 32.2, 22.9; HRMS (EI(+), 70 eV) : C_9_H_15_ClF_2_O [M-H_2_O]^+^: calcd. 194.0674, found 194.0680; IR (neat) cm^-1^ ṽ: 3085, 2952, 2865, 1645, 1453, 1394, 1303, 1261, 1096, 1031, 933, 862, 802, 744, 650; [α]_D_^20^ = + 13.3 (c = 0.22, CH_2_Cl_2_); Enantiomeric excess was determined by HPLC analysis of the 3,5-nitrobenzoatederivative of the product (Chiralcel AD-H column, CO_2_:i-PrOH = 95:5, 1.3mL/min, 214 nm), t_minor_ = 8.1min, t_major_ = 10.3 min, 96% ee.

**(3R,4S)-4-(difluoromethyl)-1-phenylhex-5-en-3-ol (3n)**

According to the general procedure with method D, **3n** (30.0 mg, 0.133 mmol) was prepared from Phenylpropyl aldehyde (26.8 mg, 0.20 mmol)as yellow oil in 66% yield.

^1^H NMR (400 MHz, CDCl_3_) δ 7.32 – 7.24 (m, 2H), 7.24 – 7.17 (m, 3H), 6.06 – 5.73 (m, 2H), 5.40 (d, *J* = 10.4 Hz, 1H), 5.28 (d, *J* = 17.6 Hz, 1H), 4.05 – 3.93 (m, 1H), 2.86 – 2.72 (m, 1H), 2.72 – 2.59 (m, 1H), 2.57 – 2.42 (m, 1H), 1.89 – 1.72 (m, 2H);^19^F NMR (376 MHz, CDCl_3_) δ -121.65 - -121.91 (m, 2F); ^13^C NMR (100 MHz, CDCl_3_) δ 141.3, 129.0 (t, *J* = 4.9 Hz), 128.5, 128.4, 126.0, 122.3, 116.9 (t, *J* = 241.3 Hz), 68.8 (t, *J* = 4.6 Hz), 53.6 (t, *J* = 19.0 Hz), 36.7, 31.9; HRMS (EI(+), 70 eV) : C_13_H_16_F_2_O [M]^+^: calcd. 226.1169, found 226.1173; IR (neat) cm^-1^ ṽ: 3076, 3027, 2954, 2860, 1642, 1603, 1496, 1395, 1298, 1259, 1090, 1021, 932, 865, 796, 749, 699; [α]_D_^20^ = + 27.5 (c = 1.37, CH_2_Cl_2_); HPLC (Chiralcel IE column, hexanes:i-PrOH = 90:10, 1.0 mL/min, 210 nm), t_major_ = 6.3 min, t_minor_ = 6.9 min, 92% ee.

**(3R,4S,6S)-3-(difluoromethyl)-6,10-dimethylundeca-1,9-dien-4-ol (3o)**

According to the general procedure with method D, **3o** (25.1 mg, 0.102 mmol) was prepared from citronellal (30.9 mg, 0.20 mmol) as yellow oil in 51% yield.

^1^H NMR (400 MHz, CDCl_3_) δ6.11 – 5.75 (m, 2H), 5.39 (d, *J* = 10.4 Hz, 1H), 5.27 (d, *J* = 16.8 Hz, 1H), 5.08 (t, *J* = 6.8 Hz, 1H), 4.15 – 3.98 (m, 1H), 2.53 – 2.35 (m, 1H), 2.05 – 1.80 (m, 2H), 1.68 (s, 3H), 1.59 (s, 3H), 1.44 – 1.08 (m, 5H), 0.97 – 0.86 (m, 3H); ^19^F NMR (376 MHz, CDCl_3_) δ -121.70 – -122.03 (m); ^13^C NMR (100 MHz, CDCl_3_) δ 131.5, 129.3 (t, *J* = 5.2 Hz), 124.5, 122.1, 117.0 (t, *J* = 240.9 Hz), 67.3 (t, *J* = 4.6 Hz), 53.2 (t, *J* = 19.1 Hz), 42.2, 36.6, 28.8, 25.4, 25.2, 19.0; 131.4, 129.1(t, *J*= 5.2 Hz), 124.5, 122.1, 116.9 (t, *J* = 240.9 Hz), 67.0 (t, *J* = 4.6 Hz), 54.1 (t, *J* = 19.1 Hz), 42.2, 37.6, 28.5, 25.7, 20.0, 17.7; HRMS (EI(+), 70 eV) : C_14_H_24_F_2_O [M]^+^: calcd. 246.1795, found 246.1805; IR (neat) cm^-1^ ṽ: 2963, 2918, 2853, 1454, 1409, 1296, 1261, 1093, 1024, 865, 802, 691; [α]_D_^20^ = + 42.9 (c = 0.16, CH_2_Cl_2_);Enantiomeric excess was determined by HPLC analysis of the 3,5-nitrobenzoatederivative of the product (Chiralcel IE column, hexanes:i-PrOH = 90:10, 1.0 mL/min, 210 nm), t_major_ = 4.9min, t_minor_ = 5.2 min, 98% ee.

**(3R,4S,E)-4-(fluoromethyl)-1-phenylhexa-1,5-dien-3-ol (3p)**

According to the general procedure with method D, **3p** (14.0 mg, 0.068 mmol) was prepared from cinnamaldehyde (26.4 mg, 0.20 mmol) as yellow oil in 34% yield (dr> 10:1).

^1^H NMR (400 MHz, CDCl_3_) δ 7.39 (d, *J* = 7.6 Hz, 2H), 7.32 (t, *J* = 7.2 Hz, 2H), 7.29 – 7.22 (m, 1H), 6.64 (d, *J* = 16.0 Hz, 1H), 6.24 (dd, *J* = 16.0, 6.8 Hz, 1H), 5.95 – 5.76 (m, 1H), 5.32 (d, *J* = 10.0 Hz, 1H), 5.26(d, *J* = 17.6 Hz, 1H), 4.75 – 4.51 (m, 2H), 4.50 – 4.43 (m, 1H), 2.72 – 2.56 (m, 1H),1.89 (br, 1H); ^19^F NMR (376 MHz, CDCl_3_) δ -225.01 - -225.40 (m, 1F); ^13^C NMR (100 MHz, CDCl_3_) δ 136.4, 133.1 (d, *J* = 6.6 Hz), 131.9, 129.5, 128.6, 127.9, 126.5, 120.0, 83.7(d, *J* = 169.1 Hz), 71.6 (d, *J* = 5.2 Hz), 50.5 (d, *J* = 17.9 Hz); HRMS (EI(+), 70 eV) : C_13_H_15_lFO [M]^+^: calcd. 206.1107, found 206.1109; IR (neat) cm^-1^ ṽ: 3074, 3028, 2967, 2908, 2863, 1814, 1715, 1646, 1605, 1542, 1494, 1389, 1296, 1256, 1067, 974, 925, 799, 753, 697; [α]_D_^20^ = + 5.3 (c = 0.21, CH_2_Cl_2_); HPLC (Chiralcel OD-H column,hexanes:i-PrOH = 97:3, 1.0 mL/min, 210 nm), t_minor_ =18.3 min, t_major_ = 25.4 min, 94% ee.

**(3R,4S,E)-4-(fluoromethyl)-1-(4-fluorophenyl)hexa-1,5-dien-3-ol (3q)**

According to the general procedure with method D, **3q** (21.1 mg, 0.094 mmol) was prepared from 4-fluorocinnamaldehyde (30.0 mg, 0.20 mmol) as yellow oil in 47% yield (dr = 6:1).

^1^H NMR (400 MHz, CDCl_3_) δ 7.40 – 7.28 (m, 2H), 7.01 (t, *J* = 8.8 Hz, 2H), 6.60 (d, *J* = 16.0 Hz, 1H), 6.15 (dd, *J* = 16.0, 6.8 Hz, 1H), 5.95 – 5.73 (m, 1H), 5.32 (d, *J* = 10.4 Hz, 1H), 5.26 (d, *J* = 17.2 Hz, 1H), 4.74 – 4.35 (m, 3H), 2.63 (m, 1H),1.92 (br, 1H); ^19^F NMR (376 MHz, CDCl_3_) δ -113.84 – -114.25 (m, 1.13F), -224.98 - -225.34 (m, 1F); ^13^C NMR (100 MHz, CDCl_3_) δ 162.4 (d, *J* = 245.9 Hz), 133.0 (d, *J* = 6.8 Hz), 132.5 (d, *J* = 3.4 Hz), 130.7, 129.2 (d, *J* = 2.0 Hz), 128.1 (d, *J* = 8.1 Hz), 120.1, 115.6, 115.4, 83.6 (d, *J* = 169.2 Hz), 71.5 (d, *J* = 4.9 Hz), 50.5 (d, *J* = 17.9 Hz); HRMS (EI(+), 70 eV) : C_13_H_12_F_4_O [M]^+^: calcd. 224.1013, found 224.1019; IR (neat) cm^-1^ ṽ: 3078, 2966, 2912, 2861, 1891, 1718, 1602, 1510, 1463, 1417, 1297, 1229, 1157, 1089, 1001, 975, 929, 822, 709; [α]_D_^20^ = + 6.5 (c = 0.17, CH_2_Cl_2_); HPLC (Chiralcel OD-H column, hexanes:i-PrOH = 99:1, 1.0 mL/min, 210 nm), t_major_ =22.4 min, t_minor_ = 23.9 min, 96% ee.

**(3R,4S)-1-phenyl-4-(phenylthio)hex-5-en-3-ol (4a)**

According to the general procedure with method E, **4a** (27.0 mg, 0.095 mmol) was prepared from Phenylpropyl aldehyde (13.4 mg, 0.10 mmol) as colorless oil in 95% yield. d.r.= 12:1

^1^H NMR (400 MHz, CDCl_3_): δ 7.41 – 7.36 (m, 2H), 7.30 – 7.23 (m, 5H), 7.18 (dd, *J* = 7.1, 5.2 Hz, 3H), 5.84 (ddd, *J* = 17.0, 10.2, 9.1 Hz, 1H), 5.20 – 5.07 (m, 2H), 3.79 – 3.66 (m, 2H), 2.87 – 2.78 (m, 1H), 2.64 (dt, *J* = 13.8, 8.1 Hz, 1H), 2.31 (d, *J* = 2.7 Hz, 1H), 1.90 – 1.80 (m, 2H).^13^C NMR (101 MHz, CDCl_3_): δ 141.7, 133.7, 133.5, 132.8, 128.9, 128.41, 128.36, 127.5, 125.8, 119.1, 71.1, 59.2, 35.9, 32.1; IR (neat) cm^-1^ ṽ: 3455, 3068, 3025, 2927, 2858, 1588, 1484, 1445, 1405, 1260, 1080, 1023, 922, 799, 743, 696; HRMS (EI(+), 70 eV) : C_18_H_20_OS [M]^+^: calcd. 284.1235, found. 284.1230; [α]_D_^20^ = + 24.8 (c = 3.00, CH_2_Cl_2_); HPLC (Chiralcel OD-H column, hexanes:i-PrOH = 90:10, 1.0 mL/min, 210 nm), t_minor_ = 6.7 min, t_major_ = 10.1 min, 91% ee.

**(3S,4R)-3-(phenylthio)non-1-en-4-ol (4b)**

According to the general procedure with method A, **4b** (24.0 mg, 0.095 mmol) was prepared from hexanal (10 mg, 0.10 mmol) as colorless oil in 95% yield. d.r.= 12:1

^1^H NMR (400 MHz, CDCl_3_): δ 7.45 – 7.40 (m, 2H), 7.32 – 7.24 (m, 3H), 5.85 (ddd, *J* = 17.0, 10.2, 9.0 Hz, 1H), 5.14 (ddd, *J* = 18.0, 13.7, 1.2 Hz, 2H), 3.75 – 3.67 (m, 2H), 2.25 (d, *J* = 3.1 Hz, 1H), 1.55 – 1.42 (m, 3H), 1.34 – 1.22 (m, 5H), 0.87 (t, *J* = 6.9 Hz, 3H). ^13^C NMR (101 MHz, CDCl_3_): δ 133.8, 132.7, 128.9, 127.4, 118.9, 71.9, 59.2, 34.1, 31.7, 25.4, 22.5, 14.0.IR (neat) cm^-1^ ṽ: 3442, 3073, 2959, 2928, 2861, 1582, 1470, 1408, 1260, 1088, 1022, 919, 865, 800, 742, 694. HRMS (EI(+), 70 eV) : C_15_H_22_OS [M]^+^: calcd. 250.1391, found. 250.1396; [α]_D_^20^ = +16.6 (c = 3.00, CH_2_Cl_2_); HPLC (Chiralcel OD-H column, hexanes:i-PrOH = 98:2, 0.4 mL/min, 210 nm), t_minor_ = 15.5 min, t_major_ = 18.5 min, 93% ee.

**(3S,4R)-8-chloro-3-(phenylthio)oct-1-en-4-ol (4c)**

According to the general procedure with method E, **4c** (26.0 mg, 0.096 mmol) was prepared from 5-chloropentanal (12.0 mg, 0.10 mmol) as colorless oil in 96% yield. d.r.= 12:1

^1^H NMR (400 MHz, CDCl_3_): δ 7.45 – 7.40 (m, 2H), 7.33 – 7.23 (m, 3H), 5.84 (ddd, *J* = 17.0, 10.2, 9.0 Hz, 1H), 5.16 (ddd, *J* = 18.2, 13.7, 1.2 Hz, 2H), 3.70 (d, *J* = 7.0 Hz, 2H), 3.52 (t, *J* = 6.6 Hz, 2H), 2.32 (s, 1H), 1.82 – 1.73 (m, 2H), 1.66 – 1.45 (m, 4H). ^13^C NMR (101 MHz, CDCl_3_): δ 133.7, 133.4, 132.8, 129.0 127.6, 119.1, 71.6, 59.2, 44.8, 33.3, 32.4, 23.2.IR (neat) cm^-1^ ṽ: 3466, 3405, 3070, 2957, 2926, 2860, 1638, 1582, 1471, 1444, 1408, 1298, 1260, 1085, 1021, 921, 864, 799, 743, 693, 659. HRMS (EI(+), 70 eV) : C_14_H_19_ClOS [M]^+^: calcd. 270.0845, found: 270.0847; [α]_D_^20^ = +15.9 (c = 3.00, CH_2_Cl_2_); HPLC (Chiralcel OD-H column, hexanes:i-PrOH = 98:2, 1.0 mL/min, 210 nm), t_minor_ = 10.9 min, t_major_ = 13.8 min, 93% ee.

**(3S,4R)-3-(phenylthio)octa-1,7-dien-4-ol (4d)**

According to the general procedure with method E, **4d** (20.0 mg, 0.085 mmol) was prepared from 4-pentenal (8.4 mg, 0.10 mmol) as colorless oil in 85% yield. d.r.= 12:1

^1^H NMR (400 MHz, CDCl_3_): δ 7.45 – 7.40 (m, 2H), 7.33 – 7.24 (m, 3H), 5.91 – 5.74 (m, 2H), 5.15 (dd, *J* = 17.3, 13.6 Hz, 2H), 5.07 – 4.92 (m, 2H), 3.79 – 3.67 (m, 2H), 2.30 – 2.20 (m, 2H), 2.11 (td, *J* = 14.7, 7.4 Hz, 1H), 1.66 – 1.61 (m, 2H). ^13^C NMR (101 MHz, CDCl_3_): δ 138.0, 133.8, 133.6, 132.8, 128.9, 127.5, 119.0, 115.0, 71.3, 59.2, 33.3, 30.1. IR (neat) cm^-1^ ṽ: 2963, 2918, 2855, 1639, 1583, 1409, 1260, 1085, 1019, 915, 865, 798, 745, 694.HRMS (EI(+), 70 eV) : C_14_H_18_OS [M]^+^: calcd. 234.1078, found. 234.1080; [α]_D_^20^ = +15.0 (c = 1.40, CH_2_Cl_2_); HPLC (Chiralcel OD-H column, hexanes:i-PrOH = 90:10, 1.0 mL/min, 210 nm), t_minor_ = 6.8 min, t_major_ = 7.9 min, 92% ee.

**(2R,3S)-1-(benzyloxy)-3-(phenylthio)pent-4-en-2-ol (4e)**

According to the general procedure with method E, **4e** (18.0 mg, 0.060 mmol) was prepared from benzyloxyacetaldehyde (15.0 mg, 0.10 mmol) as colorless oil in 60% yield. d.r.= 10:1

^1^H NMR (400 MHz, CDCl_3_): δ 7.44 – 7.39 (m, 2H), 7.37 – 7.21 (m, 8H), 5.86 (dt, *J* = 17.1, 9.7 Hz, 1H), 5.07 (dd, *J* = 27.2, 13.5 Hz, 2H), 4.57 – 4.48 (m, 2H), 3.96 (dd, *J* = 9.8, 5.0 Hz, 1H), 3.79 (dd, *J* = 9.3, 5.1 Hz, 1H), 3.63 – 3.57 (m, 2H), 2.63 (d, *J* = 4.0 Hz, 1H). ^13^C NMR (101 MHz, CDCl_3_): δ 137.7, 134.0, 133.6, 133.3, 132.9, 128.8 128.4, 127.7, 127.5, 118.4, 73.4, 71.7, 71.1, 55.5.IR (neat) cm^-1^ ṽ: 3447, 3068, 3030, 2921, 2860, 2328, 1956, 1875, 1666, 1584, 1467, 1369, 1313, 1258, 1210, 1103, 1018, 919, 856, 789, 742, 696. HRMS (DART) : C_18_H_20_O_2_S [M+NH_4_]^+^: calcd. 318.1184 found. 318.1521; [α]_D_^20^ = + 6.0 (c = 2.70, CH_2_Cl_2_); HPLC (Chiralcel OD-H column, hexanes:i-PrOH = 95:5, 0.5 mL/min, 210 nm), t_minor_ = 18.3 min, t_major_ = 27.7 min, 90% ee. The reported value^[9]^ for the (2S,3R)-enantiomer is [α]_D_^25^ = - 4.71 (c= 1.50, CHCl_3_).

**2-((3R,4S)-3-hydroxy-4-(phenylthio)hex-5-en-1-yl)isoindoline-1,3-dione (4f)**

According to the general procedure with method E, **4f** (31.0 mg, 0.088 mmol) was prepared from 3-(1,3-dioxoisoindolin-2-yl)propanal^10^ (20.3 mg, 0.10 mmol) as colorless oil in 88% yield. d.r.= 11:1

^1^H NMR (400 MHz, CDCl_3_): δ 7.88 – 7.81 (m, 2H), 7.76 – 7.69 (m, 2H), 7.43 – 7.36 (m, 2H), 7.27 – 7.19 (m, 3H), 5.85 (dt, *J* = 17.1, 9.8 Hz, 1H), 5.08 (dd, *J* = 34.4, 13.3 Hz, 2H), 3.94 – 3.80 (m, 2H), 3.78 – 3.71 (m, 1H), 3.63 (dd, *J* = 9.4, 4.0 Hz, 1H), 2.93 (d, *J* = 4.2 Hz, 1H), 1.96 – 1.79 (m, 2H). ^13^C NMR (101 MHz, CDCl_3_): δ 168.6, 134.0, 133.8, 133.5, 133.0, 132.0, 128.8, 127.5, 123.3, 118.8, 69.5, 58.9, 34.8, 33.4. IR (neat) cm^-1^ ṽ: 3467, 3067, 2925, 2858, 1769, 1705, 1623, 1439, 1397, 1128, 1079, 954, 924, 792, 720.HRMS (DART): C_20_H_19_NO_3_S [M+H]^+^: calcd. 354.1086, found. 354.1159; [α]_D_^20^ = - 2.2 (c = 0.80, CH_2_Cl_2_); HPLC (Chiralcel OD-H column, hexanes:i-PrOH = 95:5, 1.0 mL/min, 210 nm), t_minor_ = 22.5 min, t_major_ = 28.5 min, 91% ee.

**(3R,4S)-1-(methylthio)-4-(phenylthio)hex-5-en-3-ol (4g)**

According to the general procedure with method E, **4g** (21.0 mg, 0.082 mmol) was prepared from 3-(methylthio)propionaldehyde (10.4 mg, 0.10 mmol) as colorless oil in 82% yield. d.r.= 12:1

^1^H NMR (400 MHz, CDCl_3_): δ 7.43 (dd, *J* = 8.0, 1.4 Hz, 2H), 7.33 – 7.24 (m, 3H), 5.91 – 5.79 (m, 1H), 5.15 (dd, *J* = 19.2, 13.7 Hz, 2H), 3.87 (dt, *J* = 8.4, 4.2 Hz, 1H), 3.70 (dd, *J* = 9.3, 4.1 Hz, 1H), 2.72 – 2.54 (m, 2H), 2.49 (d, *J* = 4.0 Hz, 1H), 2.09 (s, 3H), 1.86 – 1.78 (m, 2H). ^13^C NMR (101 MHz, CDCl_3_): δ 133.8, 133.5, 132.8, 128.9, 127.6, 119.1, 70.9, 59.2, 33.4, 30.7, 15.5.IR (neat) cm^-1^ ṽ: 3400, 3069, 2920, 2855, 1725, 1639, 1582, 1474, 1430, 1301, 1260, 1074, 1020, 920, 865, 796, 743, 693. HRMS (EI(+), 70 eV) : C_13_H_18_OS_2_ [M]^+^: calcd: 254.0799, found: 254.0802; [α]_D_^20^ = +21.5 (c = 2.50, CH_2_Cl_2_). HPLC (Chiralcel OD-H column, hexanes:i-PrOH = 90:10, 1.0 mL/min, 210 nm), t_minor_ = 5.5 min, t_major_ = 7.1 min, 91% ee.

**(3S,4R,E)-3-(phenylthio)nona-1,5-dien-4-ol (4h)**

According to the general procedure with method E, **4h** (23.0 mg, 0.093 mmol) was prepared from hex-2-enal (9.8 mg, 0.10 mmol) as colorless oil in 93% yield. d.r.= 17:1

^1^H NMR (400 MHz, CDCl_3_): δ 7.46 – 7.40 (m, 2H), 7.33 – 7.21 (m, 3H), 5.84 (ddd, *J* = 17.0, 10.1, 9.1 Hz, 1H), 5.77 – 5.69 (m, 1H), 5.50 (ddt, *J* = 15.3, 6.8, 1.3 Hz, 1H), 5.13 (ddd, *J* = 17.8, 13.6, 0.8 Hz, 2H), 4.25 – 4.18 (m, 1H), 3.75 (dd, *J* = 9.0, 4.5 Hz, 1H), 2.34 (d, *J* = 4.1 Hz, 1H), 2.03 (q, *J* = 7.0 Hz, 2H), 1.46 – 1.35 (m, 2H), 0.90 (t, *J* = 7.4 Hz, 3H). ^13^C NMR (101 MHz, CDCl_3_): δ 134.4, 134.3, 133.9, 132.7 128.9, 128.8, 127.4 118.6, 73.3, 59.7, 34.3, 22.1, 13.6. IR (neat) cm-1 ṽ: 3408, 3067, 2958, 2923, 2865, 1722, 1667, 1582, 1471, 1444, 1386, 1302, 1260, 1085, 1024, 971, 921, 800, 742, 693.HRMS (EI(+), 70 eV) : C_15_H_20_OS [M]+: calcd. 248.1235, found. 248.1239; [α]D^20^ = 16.8 (c = 2.50, CH_2_Cl_2_). HPLC (Chiralcel OD-H column, hexanes:i-PrOH = 90:10, 1.0 mL/min, 210 nm), t_minor_ = 4.4 min, t_major_ = 5.0 min, 90% ee.

**(3R,4S,E)-1-phenyl-4-(phenylthio)hexa-1,5-dien-3-ol (4i)**

According to the general procedure with method E, **4i** (26.0 mg, 0.092 mmol) was prepared from cinnamaldehyde (13.2 mg, 0.10 mmol) as colorless oil in 92% yield. d.r.= 16:1

^1^H NMR (400 MHz, CDCl_3_): δ 7.48 – 7.42 (m, 2H), 7.40 – 7.34 (m, 2H), 7.34 – 7.27 (m, 4H), 7.27 – 7.21 (m, 2H), 6.64 (d, *J* = 15.8 Hz, 1H), 6.23 (dd, *J* = 15.9, 6.5 Hz, 1H), 5.90 (ddd, *J* = 17.0, 10.2, 9.0 Hz, 1H), 5.18 (dd, *J* = 13.6, 9.4 Hz, 2H), 4.43 (dd, *J* = 9.7, 4.5 Hz, 1H), 3.86 (dd, *J* = 8.9, 4.4 Hz, 1H), 2.54 (d, *J* = 4.5 Hz, 1H). ^13^C NMR (101 MHz, CDCl_3_): δ 134.0, 133.6, 132.8, 132.3, 129.0, 128.5, 128.2, 127.8, 127.6, 126.6, 119.1, 73.2, 59.6.IR (neat) cm^-1^ ṽ: 3367, 3064, 3027, 2922, 2855, 1717, 1660, 1587, 1482, 1442, 1309, 1261, 1084, 1024, 973, 923, 801, 745, 695. HRMS (EI(+), 70 eV) : C_18_H_18_OS [M]^+^: calcd. 282.1078, found. 282.1085; [α]_D_^20^ = + 59.9 (c = 2.00, CH_2_Cl_2_); HPLC (Chiralcel OD-H column, hexanes:i-PrOH = 90:10, 1.0 mL/min, 210 nm), t_minor_ = 8.8 min, t_major_ = 12.8 min, 95% ee.

**(3R,4S,E)-1-(4-chlorophenyl)-4-(phenylthio)hexa-1,5-dien-3-ol (4j)**

According to the general procedure with method E, **4j** (28.0 mg, 0.088 mmol) was prepared from 4-chlorocinnamaldehyde (16.7 mg, 0.10 mmol) as colorless oil in 88% yield.d.r.= 16:1

^1^H NMR (400 MHz, CDCl_3_): δ 7.44 (d, *J* = 6.9 Hz, 2H), 7.33 – 7.24 (m, 7H), 6.60 (d, *J* = 16.0 Hz, 1H), 6.19 (dd, *J* = 15.9, 6.3 Hz, 1H), 5.95 – 5.83 (m, 1H), 5.19 (dd, *J* = 13.5, 10.0 Hz, 2H), 4.41 (dd, *J* = 9.7, 4.7 Hz, 1H), 3.84 (dd, *J* = 8.9, 4.5 Hz, 1H), 2.57 (d, *J* = 4.4 Hz, 1H). ^13^C NMR (101 MHz, CDCl_3_): δ 134.8, 133.9, 133.5, 133.4, 132.8, 131.0, 129.0, 128.9, 128.7, 127.8, 127.6, 119.2, 73.0, 59.5. IR (neat) cm^-1^ ṽ: 3414, 3068, 2924, 2856, 1664, 1586, 1485, 1407, 1301, 1262, 1089, 1017, 971, 924, 811, 741, 695. HRMS (DART) : C_18_H_17_ClOS [M+H]^+^: calcd. 317.0689, found. 317.0761; [α]_D_^20^ = +45.3 (c = 2.30, CH_2_Cl_2_). HPLC (Chiralcel IE-H column, hexanes:i-PrOH = 90:10, 1.0 mL/min, 210 nm), t_minor_ = 6.7 min, t_major_ = 8.4 min, 93% ee.

**(3R,4S,E)-1-(4-bromophenyl)-4-(phenylthio)hexa-1,5-dien-3-ol (4k)**

According to the general procedure with method E, **4k** (32.0 mg, 0.089 mmol) was prepared from 4-bromocinnamaldehyde (21.1 mg, 0.10 mmol) as colorless oil in 89% yield. d.r.= 16:1

^1^H NMR (400 MHz, CDCl_3_): δ 7.47 – 7.39 (m, 4H), 7.33 – 7.26 (m, 3H), 7.24 – 7.20 (m, 2H), 6.59 (d, *J* = 15.6 Hz, 1H), 6.21 (dd, *J* = 15.9, 6.3 Hz, 1H), 5.89 (ddd, *J* = 17.0, 10.2, 8.9 Hz, 1H), 5.19 (dd, *J* = 13.6, 9.6 Hz, 2H), 4.41 (dd, *J* = 9.6, 4.7 Hz, 1H), 3.84 (dd, *J* = 8.9, 4.5 Hz, 1H), 2.54 (d, *J* = 4.6 Hz, 1H). ^13^C NMR (101 MHz, CDCl_3_): δ 135.3, 133.9, 133.5, 132.8, 131.6, 131.0, 129.0, 128.1, 127.7, 121.6, 119.3, 73.0, 59.5. IR (neat) cm^-1^ ṽ: 3421, 3067, 2955, 2924, 2857, 1732, 1643, 1584, 1482, 1444, 1404, 1304, 1260, 1080, 1019, 972, 925, 801, 742, 694.HRMS (DART) : C18H17BrOS [M+H]^+^: calcd. 361.0183, found. 361.0255;

[α]_D_^20^ = + 36.4 (c = 2.20, CH_2_Cl_2_). HPLC (Chiralcel IE-H column, hexanes:i-PrOH = 85:15, 1.0 mL/min, 210 nm), t_minor_ = 7.3 min, t_major_ = 9.3 min, 93% ee.

**(3R,4S,E)-1-(3-fluorophenyl)-4-(phenylthio)hexa-1,5-dien-3-ol (4l)**

According to the general procedure with method E, **4l** (25.0 mg, 0.083 mmol) was prepared from 3-fluorocinnamaldehyde (15.0 mg, 0.10 mmol) as colorless oil in 83% yield. d.r.= 15:1

^1^H NMR (400 MHz, CDCl_3_): δ 7.45 (dd, *J* = 8.0, 1.3 Hz, 2H), 7.34 – 7.22 (m, 4H), 7.12 (d, *J* = 7.7 Hz, 1H), 7.05 (d, *J* = 10.1 Hz, 1H), 6.93 (td, *J* = 8.5, 2.4 Hz, 1H), 6.62 (d, *J* = 15.9 Hz, 1H), 6.23 (dd, *J* = 15.9, 6.2 Hz, 1H), 5.89 (ddd, *J* = 16.9, 10.2, 9.0 Hz, 1H), 5.20 (dd, *J* = 13.5, 9.2 Hz, 2H), 4.42 (dd, *J* = 9.6, 4.7 Hz, 1H), 3.85 (dd, *J* = 8.9, 4.5 Hz, 1H), 2.58 (d, *J* = 4.5 Hz, 1H). ^13^C NMR (101 MHz, CDCl_3_): δ 163.0 (d, *J_C-F_* = 246.4 Hz) , 138.7 (d, *J_C-F_* = 7.8 Hz), 133.8, 133.5, 132.8, 131.0 (d, *J_C-F_* = 2.6 Hz), 130.0 (d, *J_C-F_* = 8.4 Hz), 129.7, 129.0, 127.7, 122.5 (d, *J_C-F_* = 2.7 Hz), 119.3, 114.6 (d, *J_C-F_* = 22.2 Hz),113.0 (d, *J_C-F_* = 21.2 Hz), 72.9, 59.5.IR (neat) cm^-1^ ṽ: 3400, 3068, 2920, 2856, 1585, 1481, 1260, 1088, 1024, 970, 870, 798, 745, 689.HRMS (EI(+), 70 eV) : C_18_H_17_FOS [M]^+^: calcd. 300.0984, found. 300.0981; [α]_D_^20^ = + 30.1 (c = 3.00, CH_2_Cl_2_); HPLC (Chiralcel OD-H column, hexanes:i-PrOH = 90:10, 1.0 mL/min, 210 nm), t_minor_ = 7.1 min, t_major_ = 9.6 min, 93% ee.

**(3R,4S,E)-4-(phenylthio)-1-(o-tolyl)hexa-1,5-dien-3-ol (4m)**

According to the general procedure with method E, **4m** (27 mg, 0.091 mmol) was prepared from 2-methylcinnamaldehyde (14.6 mg, 0.10 mmol) as colorless oil in 91% yield. d.r.= 20:1

^1^H NMR (400 MHz, CDCl_3_): δ 7.48 – 7.42 (m, 2H), 7.39 (dd, *J* = 8.0, 4.6 Hz, 1H), 7.34 – 7.23 (m, 3H), 7.19 – 7.11 (m, 3H), 6.86 (d, *J* = 15.8 Hz, 1H), 6.10 (dd, *J* = 15.7, 6.5 Hz, 1H), 5.91 (ddd, *J* = 17.0, 10.2, 9.0 Hz, 1H), 5.18 (dd, *J* = 13.6, 10.1 Hz, 2H), 4.45 (dd, *J* = 9.6, 4.7 Hz, 1H), 3.86 (dd, *J* = 8.9, 4.5 Hz, 1H), 2.54 (d, *J* = 4.7 Hz, 1H), 2.34 (s, 3H). ^13^C NMR (101 MHz, CDCl_3_): δ 135.6, 134.1, 133.7, 132.8, 130.21, 130.16, 129.6, 129.0, 127.7, 127.5, 126.0 125.9, 119.0, 73.4, 59.7, 19.8.IR (neat) cm^-1^ ṽ: 3419, 3063, 3019, 2922, 2859, 1665, 1641, 1584, 1476, 1446, 1384, 1299, 1267, 1223, 1080, 1027, 970, 922, 790, 745, 693.HRMS (EI(+), 70 eV) : C_19_H_20_OS [M]^+^: calcd. 296.1235, found. 296.1237; [α]_D_^20^ = + 37.4 (c = 3.40, CH_2_Cl_2_); HPLC (Chiralcel OD-H column, hexanes:i-PrOH = 90:10, 1.0mL/min, 230 nm), t_minor_ = 7.6 min, t_major_ = 10.4 min, 93% ee.

**(3R,4S,E)-1-(2-methoxyphenyl)-4-(phenylthio)hexa-1,5-dien-3-ol (4n)**

According to the general procedure with method E, **4n** (27.0 mg, 0.087 mmol) was prepared from 2'-methoxycinnamaldehyde (16.2 mg, 0.10 mmol) as colorless oil in 87% yield. d.r.= 18:1

^1^H NMR (400 MHz, CDCl_3_): δ 7.45 (t, *J* = 1.8 Hz, 1H), 7.45 – 7.43 (m, 1H), 7.40 (dd, *J* = 7.6, 1.7 Hz, 1H), 7.32 – 7.20 (m, 4H), 7.00 – 6.83 (m, 3H), 6.25 (dd, *J* = 16.0, 6.8 Hz, 1H), 5.92 (ddd, *J* = 17.0, 10.2, 9.0 Hz, 1H), 5.21 – 5.11 (m, 2H), 4.49 – 4.41 (m, 1H), 3.86 (dd, *J* = 9.0, 4.4 Hz, 1H), 3.83 (s, 3H), 2.49 (d, *J* = 4.5 Hz, 1H). ^13^C NMR (101 MHz, CDCl_3_): δ 156.8, 134.2, 133.8, 132.8, 128.9, 128.8, 127.4, 127.2, 127.0, 125.3, 120.5, 118.8, 110.8, 73.8, 59.6, 55.4.IR (neat) cm^-1^ ṽ: 3444, 3061, 2924, 2841, 2741, 1667, 1601, 1511, 1463, 1304, 1251, 1175, 1129, 1029, 971, 924, 819, 745, 693. HRMS (DART) : C_19_H_20_O_2_S [M+Na]^+^: calcd. 335.1184., found. 335.1081;[α]_D_^20^ = +41.8 (c = 3.00, CH_2_Cl_2_); HPLC (Chiralcel OJ-H column, hexanes:i-PrOH = 91:9, 0.7 mL/min, 214 nm), t_major_ = 36.9 min, t_minor_ = 41.1 min, 94% ee.

**(5R,8R,9S,10S,13R,14S,17R)-17-((2R,5R,6S)-5-hydroxy-6-(trimethylsilyl)oct-7-en-2-yl)-10,13-dimethyltetradecahydro-1H-cyclopenta[a]phenanthren-3(2H)-one (5)**

According to the general procedure with method A, **5** (80.3 mg, 0.170 mmol) was prepared from (R)-4-((5R,8R,9S,10S,13R,14S,17R)-10,13-dimethyl-3-oxohexadecahydro

-1H-cyclopenta[a]phenanthren-17-yl)pentanal^[12]^ (71.70 mg, 0.20 mmol) as yellow oil in 85% yield.

^1^H NMR (400 MHz, CDCl_3_): δ 5.78 (dt, *J* = 17.1, 10.4 Hz, 1H), 4.95 (ddd, *J* = 18.8, 13.6, 1.8 Hz, 2H), 3.75 – 3.68 (m, 1H), 2.73 – 2.62 (m, 1H), 2.31 (td, *J* = 14.5, 5.2 Hz, 1H), 2.13 (d, *J* = 14.5 Hz, 1H), 2.01 (dd, *J* = 9.0, 6.4 Hz, 3H), 1.92 – 1.72 (m, 4H), 1.65 (dd, *J* = 10.6, 5.1 Hz, 1H), 1.61 – 1.53 (m, 1H), 1.53 – 1.29 (m, 10H), 1.29 – 1.17 (m, 3H), 1.16 – 1.03 (m, 5H), 1.00 (s, 3H), 0.89 (d, *J* = 6.4 Hz, 3H), 0.66 (s, 3H), 0.03 (s, 9H). ^13^C NMR (101 MHz, CDCl_3_):δ 213.4, 135.8, 114.9, 72.0, 56.4, 56.0, 44.3, 42.7, 42,6, 42.3, 40.6, 40.00, 37.2, 37.0, 35.6, 35.4, 34.8, 33.8, 32.0, 28.2, 26.6, 25.7, 24.1, 22.6, 21.1, 18.7, 12.0, -2.0.

; IR (neat) cm^-1^ ṽ: 3356, 2972, 1706, 1449, 1379, 1247, 1087, 1046, 880, 838, 688; HRMS (EI(+), 70 eV) : C_30_H_52_O_2_Si [M+1]^+^: calcd. 473.3737, found 473.3810; [α]_D_^20^ = +24.2 (c = 1.30, CH_2_Cl_2_); Enantiomeric excess was determined by HPLC analysis of the 3,5-nitrobenzoate derivative of the product (Chiralcel IE-H column, hexanes:i-PrOH = 90:10, 1.0 mL/min, 210 nm), t_minor_ = 22.1 min, t_major_ = 23.5 min, 99% de.

**(5R,8R,9S,10S,13R,14S,17R)-17-((2R,5R,6S)-5-hydroxy-6-(trifluoromethyl)oct-7-en-2-yl)-10,13-dimethyltetradecahydro-1H-cyclopenta[a]phenanthren-3(2H)-one (6)**

According to the general procedure with method C, **6** (79.6 mg, 0.170 mmol) was prepared from (R)-4-((5R,8R,9S,10S,13R,14S,17R)-10,13-dimethyl-3-oxohexadecahydro

-1H-cyclopenta[a]phenanthren-17-yl)pentanal^[12]^ (71.70 mg, 0.20 mmol) as yellow oil in 85% yield.

^1^H NMR (400 MHz, CDCl_3_) δ 5.85 (dt, *J* = 17.2, 10.4 Hz, 1H), 5.43 (d, *J* = 10.4 Hz, 1H), 5.31 (d, *J* = 17.2 Hz, 1H), 4.02 – 3.92 (m, 1H), 2.78 – 2.59 (m, 2H), 2.31 (td, *J* = 14.4, 5.2 Hz, 1H), 2.18 – 2.09 (m, 1H), 2.09 – 1.69 (m, 7H), 1.68 – 1.00 (m, 18H), 1.00 (s, 3H), 0.90 (d, *J* = 6.4 Hz, 3H), 0.66 (s, 3H); ^19^F NMR (376 MHz, CDCl_3_) δ -67.62 (d, *J* = 9.4 Hz); ^13^C NMR (101 MHz, CDCl_3_) δ 213.6, 127.4(q, *J* = 2.4 Hz), 126.4 (q, *J* = 279.3 Hz),123.2, 68.9(q, *J* = 2.0 Hz), 56.4, 55.9, 53.5 (q, *J* = 24.4 Hz), 44.3, 42.7, 42.3, 40.6, 40.0, 37.1, 36.9, 35.4, 34.8, 31.6, 31.3, 28.2, 26.5, 25.7, 24.1, 22.6, 21.1, 18.6, 12.0; C_28_H_43_F_3_O_2_ [M]^+^: calcd. 468.3215, found 468.3207; IR (neat) cm^-1^ ṽ: 2964, 2913, 1409, 1262, 1092,1024, 866, 802, 693; [α]_D_^20^ = + 42.1 (c = 2.0, CH_2_Cl_2_);HPLC (Chiralcel IA column,hexanes:i-PrOH = 98:2, 0.7 mL/min, 214 nm), t_minor_ = 10.4 min, t_major_ = 12.2 min, 90% ee.

**(5R,8R,9S,10S,13R,14S,17R)-17-((2R,5R,6S)-5-hydroxy-6-(phenylthio)oct-7-en-2-yl)-10,13-dimethyltetradecahydro-1H-cyclopenta[a]phenanthren-3(2H)-one (7)**

According to the general procedure with method E, **7** (48.0 mg, 0.094 mmol) was prepared from (R)-4-((5R,8R,9S,10S,13R,14S,17R)-10,13-dimethyl-3-oxohexadecahydro

-1H-cyclopenta[a]phenanthren-17-yl)pentanal^[12]^ (35.8 mg, 0.10 mmol) as colorless oil in 94% yield. d.r.= 12:1

^1^H NMR (400 MHz, CDCl_3_): δ 7.42 (d, *J* = 6.8 Hz, 2H), 7.33 – 7.21 (m, 3H), 5.91 – 5.80 (m, 1H), 5.13 (dd, *J* = 21.8, 13.4 Hz, 2H), 3.74 – 3.63 (m, 2H), 2.75 – 2.63 (m, 1H), 2.39 – 2.26 (m, 2H), 2.15 (d, *J* = 14.5 Hz, 1H), 2.08 – 1.96 (m, 3H), 1.94 – 1.77 (m, 4H), 1.63 – 1.53 (m, 2H), 1.50 – 1.32 (m, 8H), 1.30 – 1.18 (m, 4H), 1.17 – 1.04 (m, 4H), 1.01 (s, 3H), 0.91 (d, *J* = 6.4 Hz, 3H), 0.67 (s, 3H). ^13^C NMR (101 MHz, CDCl_3_): δ 213.5, 134.0, 133.7, 132.7, 128.8, 127.4, 118.8, 72.2, 59.3, 56.3, 55.9, 44.3, 42.6, 42.3, 40.6, 40.0, 37.2, 36.9, 35.5, 35.4, 34.8, 31.7, 30.6, 28.2, 26.6, 25.7, 24.1, 22.6, 21.1, 18.6, 12.0.IR (neat) cm^-1^ ṽ: 3443, 3070, 2931, 2863, 1707, 1582, 1450, 1381, 1264, 1077, 1032, 995, 916, 742, 692.HRMS (DART) : C_33_H_48_O_2_S [M+NH_4_]^+^: calcd. 508.3375, found. 526.3709; [α]_D_^20^ = +20.5 (c = 6.00, CH_2_Cl_2_); HPLC (Chiralcel OD-H column, hexanes:i-PrOH = 90:10, 1.0 mL/min, 210 nm), t_minor_ = 9.4 min, t_major_ = 11.9 min, 93% ee.

**(S,E)-1-(4-bromophenyl)-4-fluorobut-2-en-1-ol (8)**

To a solution of **2m** (33.4 mg, 0.112 mmol) in 1.1 ml acetonitrile (0.1 M), NaHCO_3_ (1 equiv) and Selectfluor™ (1.5 equiv) were added. The reaction mixture was allowed to stir at room temperature for 6 hours. After this time, the reaction mixture was quenched with saturated aqueous NaHCO_3_, followed by extraction with diethyl ether. The combined organic layers were washed with brine, dried over Mg_2_SO_4_ and evaporated under reduced pressure. The crude product was purified by flash chromatography employing mixtures of *n*-hexane:ethyl acetate (5:1) as eluents to give **8** (19.1mg, 0.079mmol) as colorless oil in 70% yield.^[6,7]^

^1^H NMR (400 MHz, CDCl_3_): δ 7.48 (d, *J* = 8.4 Hz, 2H), 7.24 (d, *J* = 8.4 Hz, 2H), 6.04 – 5.87 (m, 2H), 5.25 – 5.18 (m, 1H), 4.94 (d, *J* = 2.9 Hz, 1H), 4.82 (d, *J* = 4.1 Hz, 1H), 2.13 (br, 1H).^13^C NMR (101 MHz, CDCl_3_):δ 141.0 (d, *J_C-F_* = 2.0 Hz), 135.7 (d, *J_C-F_* = 11.3 Hz), 131.7 (2C), 128.0 (d, 2C, *J_C-F_* = 2.4 Hz), 125.8 (d, *J_C-F_* = 17.1 Hz), 121.8, 82.4 (d, *J_C-F_* = 165.6 Hz), 73.4 (d, *J* = 1.1 Hz); [α]_D_^20^ = - 4.3 (c = 0.33, CH_2_Cl_2_); HPLC (Chiralcel IE-H column, hexanes:i-PrOH = 95:5, 1 mL/min, 210 nm), t_minor_ = 7.9 min, t_major_ = 8.5 min, 91% ee. The reported value^5^ for the (R,E)-enantiomer (91% ee) is [α]_D_^25^ = + 8.0 (c= 1.0; CHCl_3_).

**2S,6R)-2,6-diphenethyl-3,6-dihydro-2H-pyran (9)**

To a solution of **2a** (10.0 mg, 0.040 mmol), [phenylpropyl aldehyde](javascript:showMsgDetail('ProductSynonyms.aspx?CBNumber=CB3143311&postData3=CN&SYMBOL_Type=A');) (17 mg, 0.121 mmol) and 4 A molecular sieve (20 mg) in dry DCM (0.3 mL) at -78^o^C was added TMSOTf (4.5 mg, 0.020 mmol). After stirring at -78^o^C for 2 hours, the reaction mixture was quenched with saturated aqueous NaHCO_3_, followed by extraction with DCM. The combined organic layers were washed with brine, dried over Mg_2_SO_4_ and evaporated under reduced pressure. The crude product was purified by flash chromatography employing mixtures of *n*-hexane:ethyl acetate (20:1) as eluents to give **9** (9.6 mg, 0.033 mmol) as colorless oil in 82% yield.^[13]^

^1^H NMR (400 MHz, CDCl_3_): δ 7.35 – 7.12 (m, 10H), 5.82 – 5.75 (m, 1H), 5.61 (d, *J* = 10.2 Hz, 1H), 4.07 (s, 1H), 3.54 – 3.45 (m, 1H), 2.91 – 2.81 (m, 2H), 2.81 – 2.69 (m, 2H), 2.08 – 1.73 (m, 6H).^13^C NMR (101 MHz, CDCl_3_):δ 142.3, 130.4, 128.6, 128.5, 128.29, 128.27, 125.7, 124.9, 73.8, 72.8, 37.6, 37.2, 31.8, 31.5, 31.4. [α]_D_^20^ = -17.7 (c = 0.25, CH_2_Cl_2_).

HPLC (Chiralcel OD-H column, hexanes:i-PrOH = 95:5, 0.4 mL/min, 210 nm), t_major_ = 10.2 min,t_minor_ = 10.9 min, 94% ee.

1. **Reference:**

[1]. Chen, W., Yang, Q., Zhou, T., Tian, Q. & Zhang, G. Enantioselective synthesis of α-exo-methylene γ‑butyrolactones via chromium catalysis. *Org. Lett.* **17**, 5236−5239 (2015).

[2]. McManus, H. A. & Guiry, P. J. Coupling of bulky, electron-deficient partners in aryl amination in the preparation of tridentate bis(oxazoline) ligands for asymmetric catalysis. *J. Org. Chem*. **67**, 8566−8573 (2002).

[3]. Loh, T. & Li, X. A. Highly stereoselective synthesis of β-trifluoromethylated homoallylic alcohols in water. *Eur. J. Org. Chem.* **8**, 1893−1899 (1999).

[4]. Jagodzinska, M., Huguenot, F. & Zanda, M. Studies on a three-step preparation of β-fluoroalkyl acrylates from fluoroacetic esters. *Tetrahedron.* **63**, 2042−2046 (2007).

[5]. Tamura, K., Yamazaki, T., Kitazume, T. & Kubota, T. [The effect of fluoromethyl groups on the diastereoselectivity in the electrophilic alkylation](http://www.sciencedirect.com/science/article/pii/S0022113905001077). *J. Fluorine Chem*. **126**, 918−930 (2005).

[6]. Purser, S., Wilson, C., Moore, P. R & Gouverneur, V. Highly diastereoselective electrophilic fluorination of cyclic *syn*-β-hydroxysilanes. *Synlett.* **7**, 1166−1168 (2007).

[7]. Barrio, P., Rodriguez, E., Saito, K., Fustero, S. & Akiyama, T. γ-Silylboronates in the chiral brønsted acid-catalysed allylboration of aldehydes. *Chem. Commun*. **51**, 5246–5249 (2015).

[8]. Gao, X., Zhang, Y. J. & Krische, M. J. Iridium-catalyzed anti-diastereo- and enantioselective carbonyl(α-trifluoromethyl)allylation from the alcohol or aldehyde oxidation level. *Angew. Chem. Int. Ed.* **50**, 4173–4175 (2011).

[9]. Kang, S.-K., Park, D.-C., Jeon, J.-H., Rho, H.-S. & Yu, C.-M. Highly regioselective palladium-mediated substitution of allylic and dienylic cyclic carbonates. *Tetrahedron* *Lett.,* **35**, 2357−2360 (1994).

[10]. Nugent, B. M., Williams, A. L., Prabhakaran, E. N. & Johnston, J. N. Free radical-mediated vinyl amination: a mild, general pyrrolidinyl enamine synthesis. *Tetrahedron*. **59**, 8877−8888 (2003).

[11].Gamedze, M. P. & Nkambule, C. M. Dibutyltin oxide mediated diastereoselective

cyclodehydration/sulfonylation of 1,2,4-triols. *Tetrahedron Letters*. **56**, 1825−1829 (2015).

[12]. Paradisi, M. P. & Zecchini, G. P. Reduction of steroidal dicarbonyl compounds with poly

(*n*-isopropyliminoalane). *Tetrahedron.* *37*, 971−975 (1981).

[13]. Roush, W. R. & Dilley, G. J. Studies on the synthesis of 2,6-disubstituted dihydropyrans: intervention of oxonia-Cope rearrangements in the lewis acid mediated cyclodehydrative reactions of aldehydes and β-hydroxyallylsilanes. *Synlett*. **SI**, 955−959 (2001).

**3.Copies of NMR specta and HPLC chromatographs**

**^1^H NMR (400 MHz, CDCl_3_) 1,8-bis((S)-4-((R)-sec-butyl)-4,5-dihydrooxazol-2-yl)-9H-carbazole (L-5)**

**
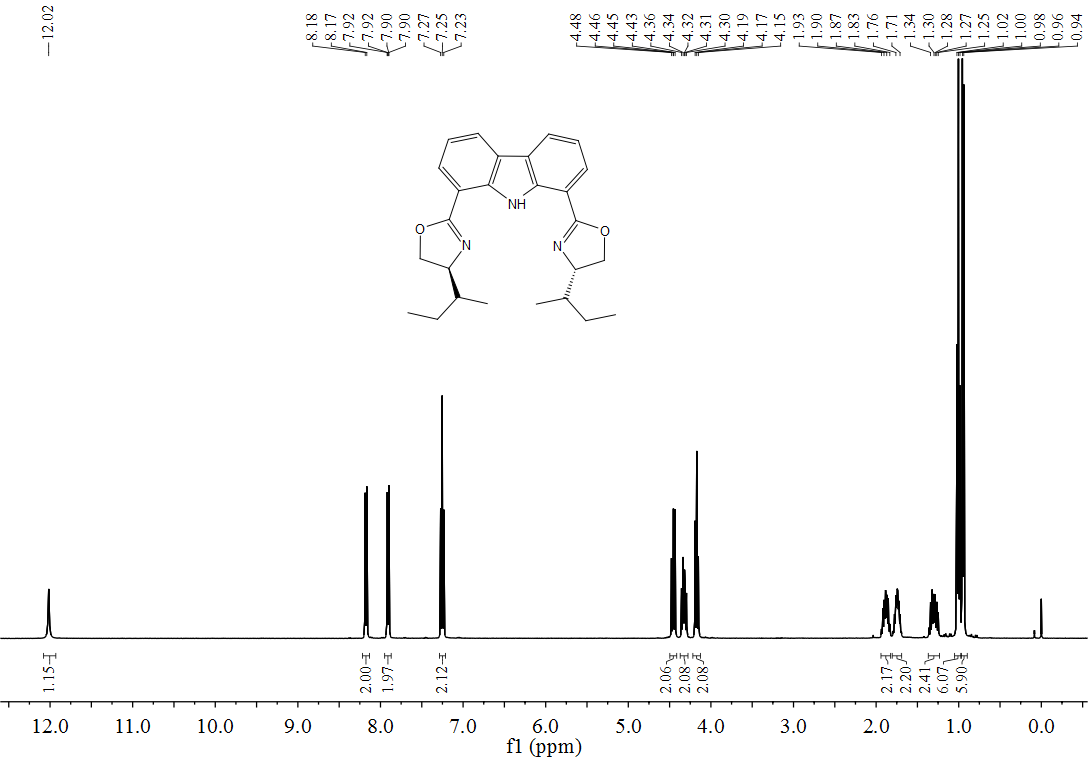
**

**^13^C NMR (400 MHz, CDCl_3_) 1,8-bis((S)-4-((R)-sec-butyl)-4,5-dihydrooxazol-2-yl)-9H-carbazole (L-5)**

**
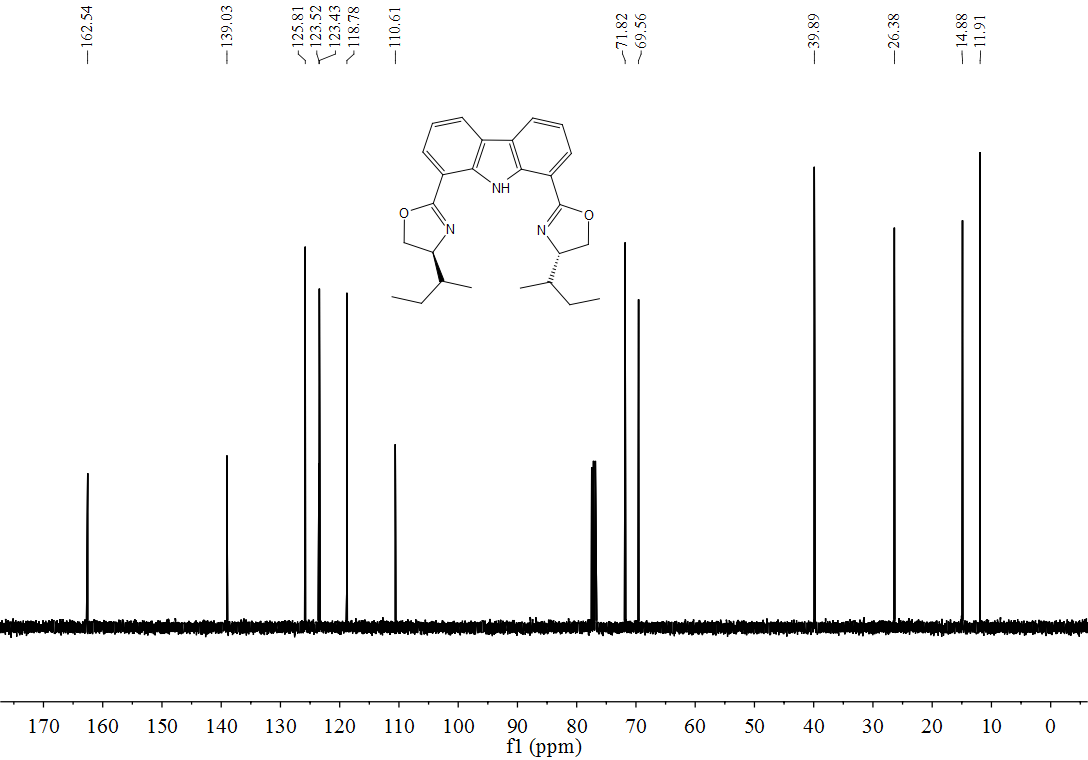
1H NMR (400 MHz, CDCl_3_) (E)-4-bromo-1,1,1-trifluorobut-2-ene**

**
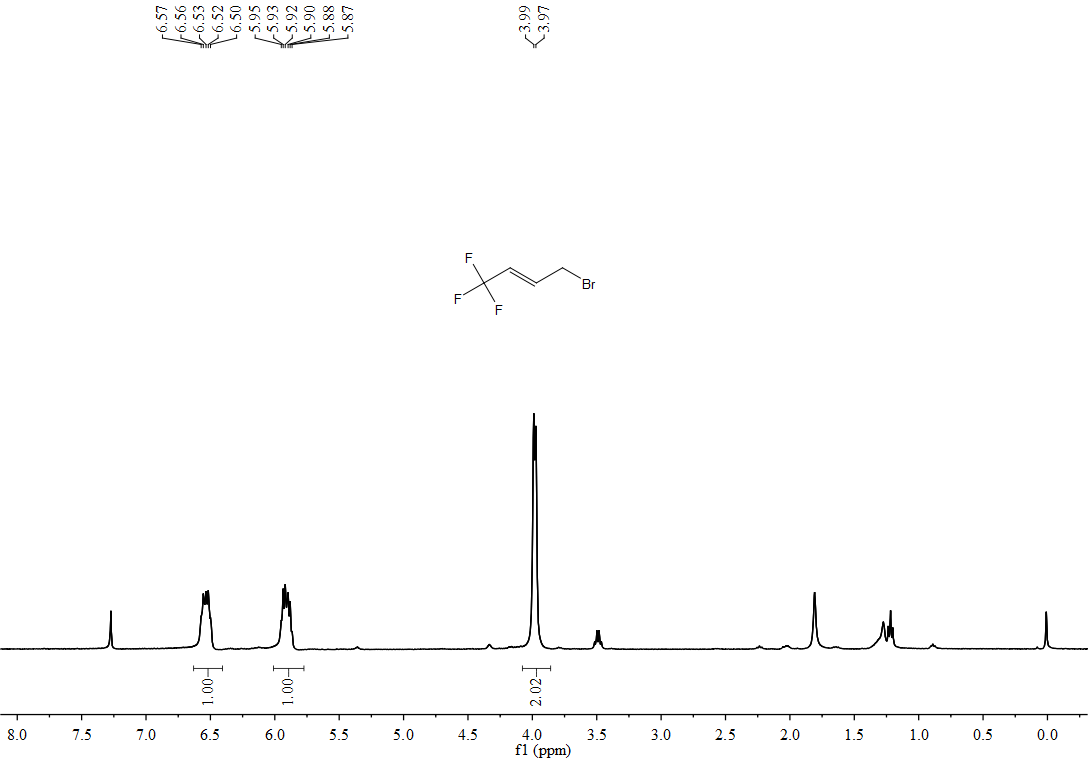
**

Diethyl ether

**
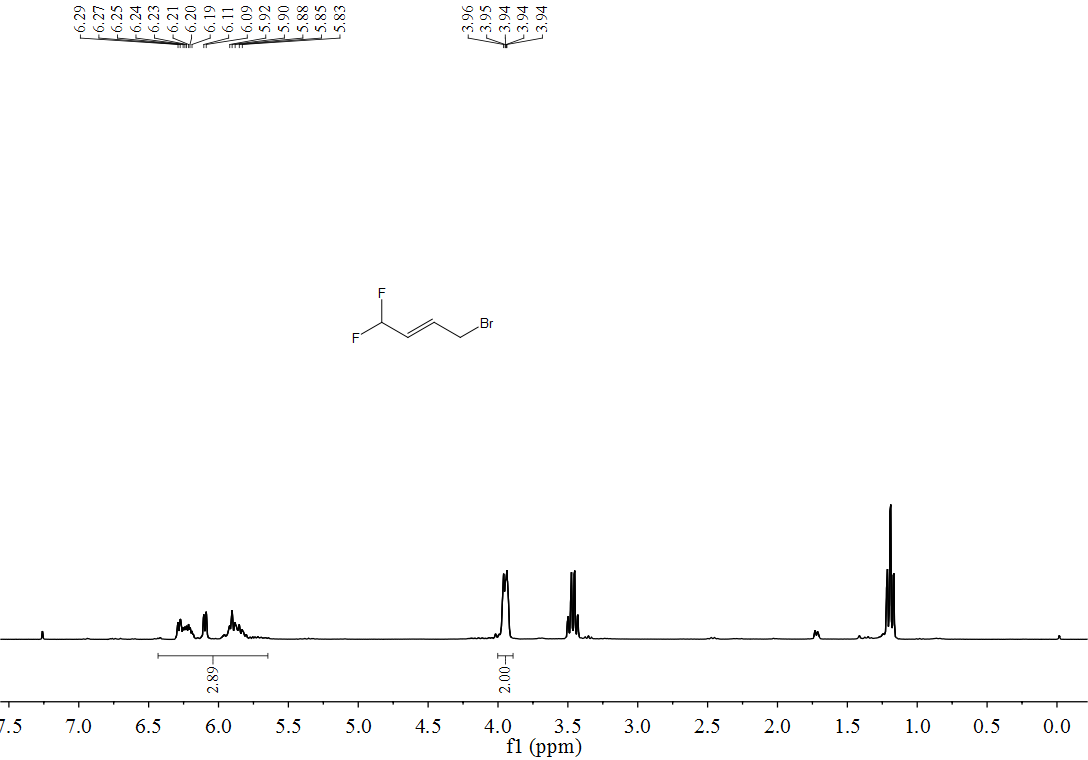
1H NMR (400 MHz, CDCl_3_) (E)-4-bromo-1,1-difluorobut-2-ene**

Diethyl ether

**^19^F NMR (376 MHz, CDCl_3_)(E)-4-bromo-1,1-difluorobut-2-ene**


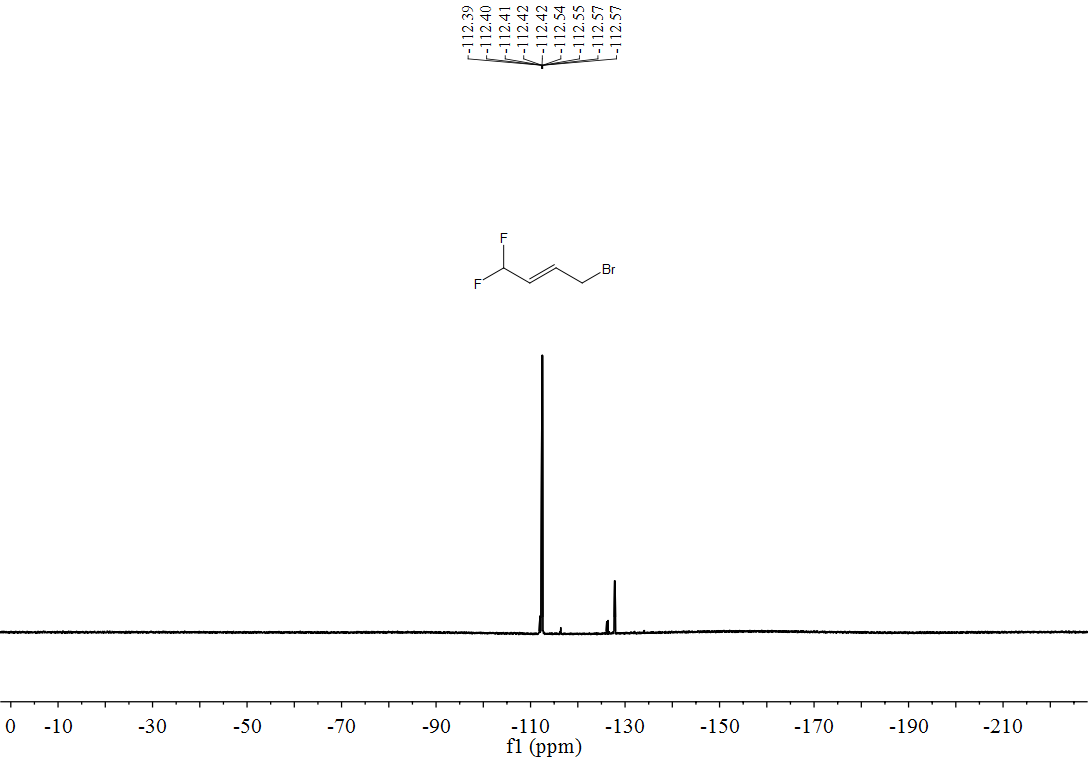


**^13^C NMR (101 MHz, CDCl_3_)(E)-4-bromo-1,1-difluorobut-2-ene**


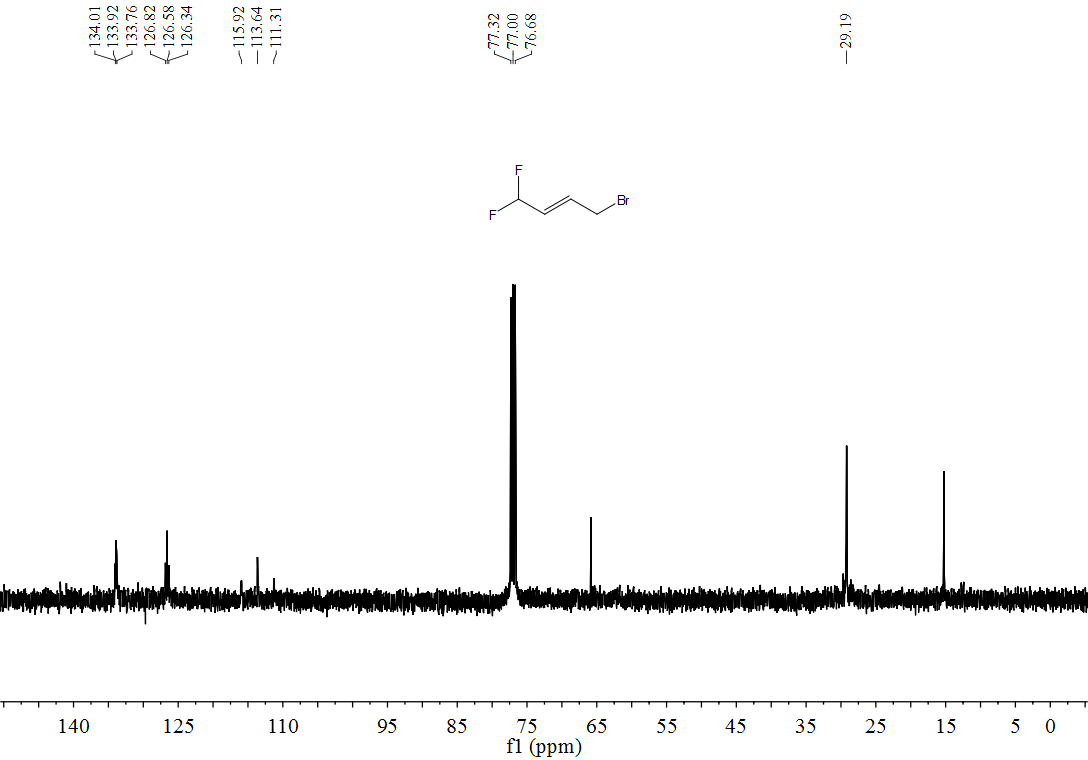


Diethyl ether


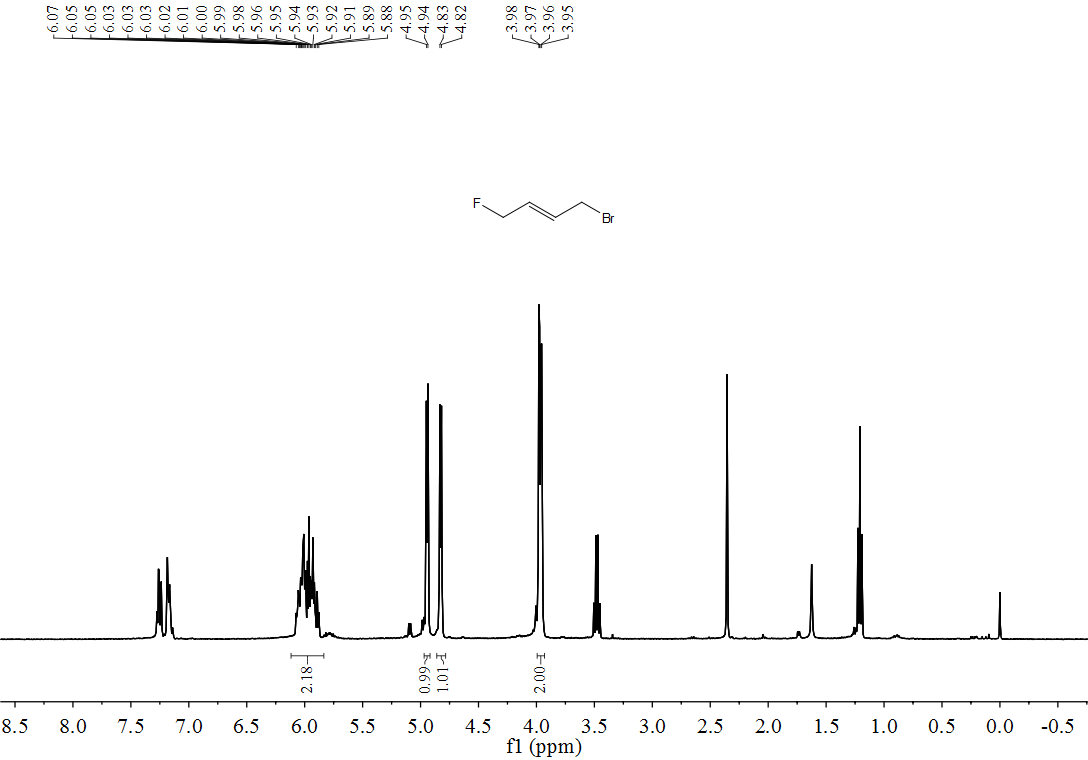
**^1^H NMR (400 MHz, CDCl_3_)(E)-1-bromo-4-fluorobut-2-ene**

Diethyl ether

toluene

**^19^F NMR (376 MHz, CDCl_3_)(E)-1-bromo-4-fluorobut-2-ene**


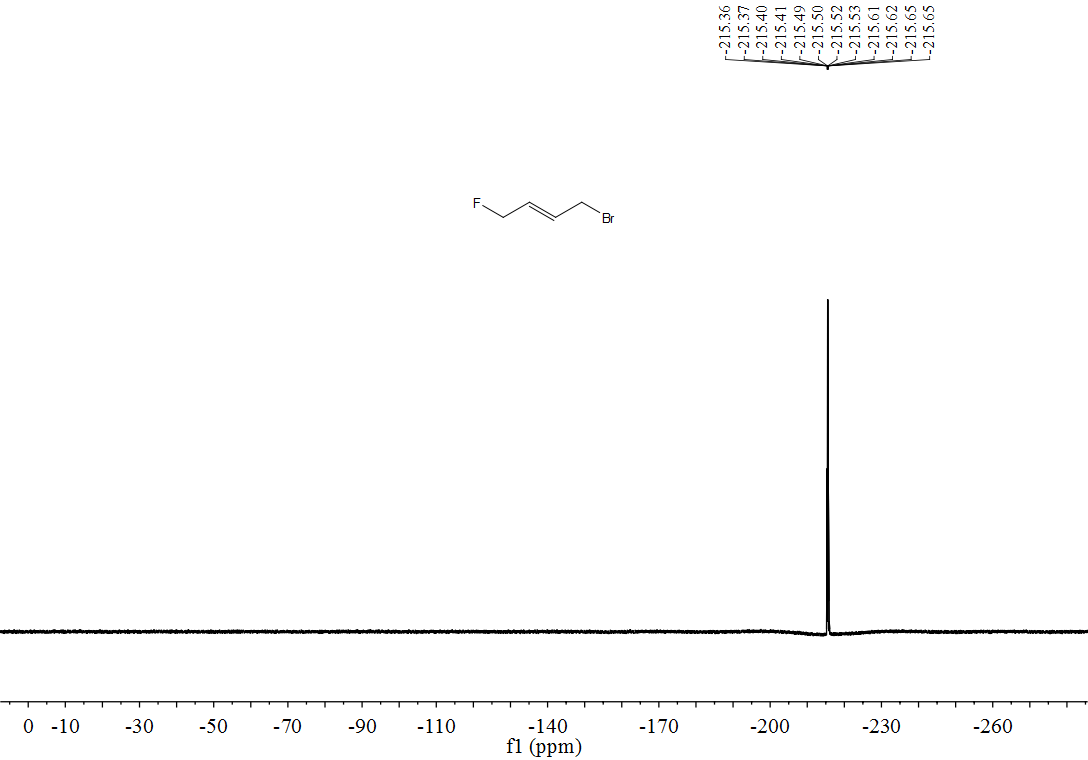


**^13^C NMR (101 MHz, CDCl_3_)(E)-1-bromo-4-fluorobut-2-ene**

Diethyl ether

Diethyl ether

**
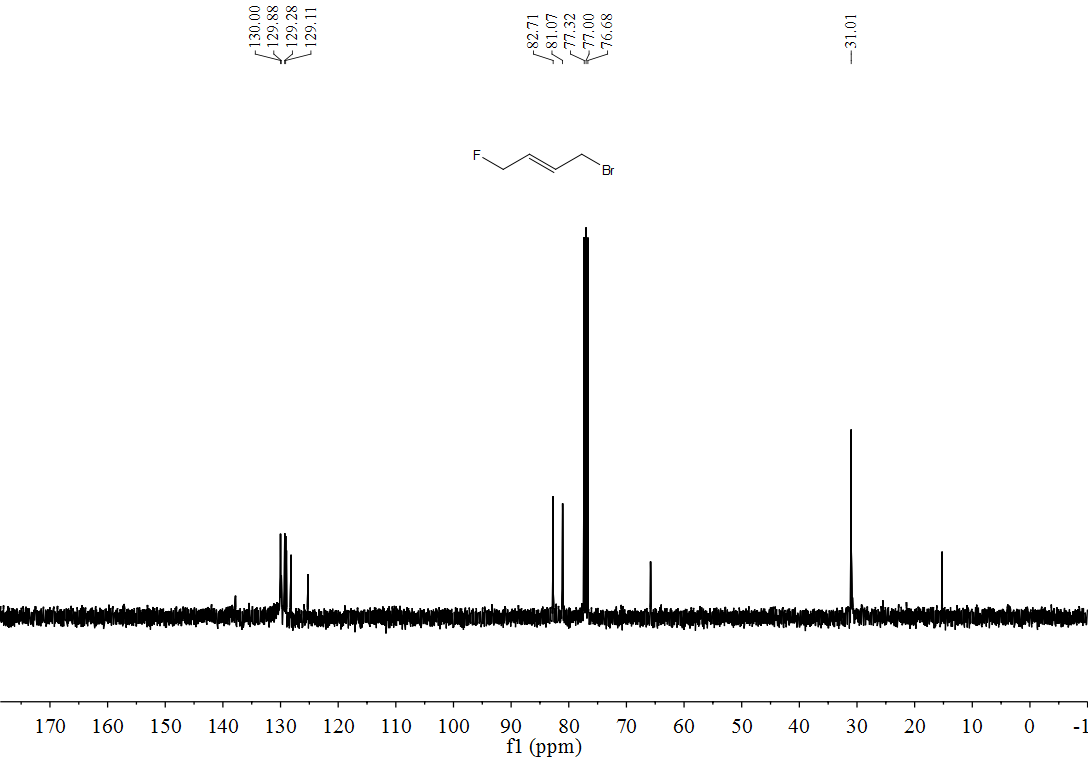
**

**^1^H NMR (400 MHz, CDCl_3_) (3R,4S)-1-phenyl-4-(trimethylsilyl)hex-5-en-3-ol (2a)**


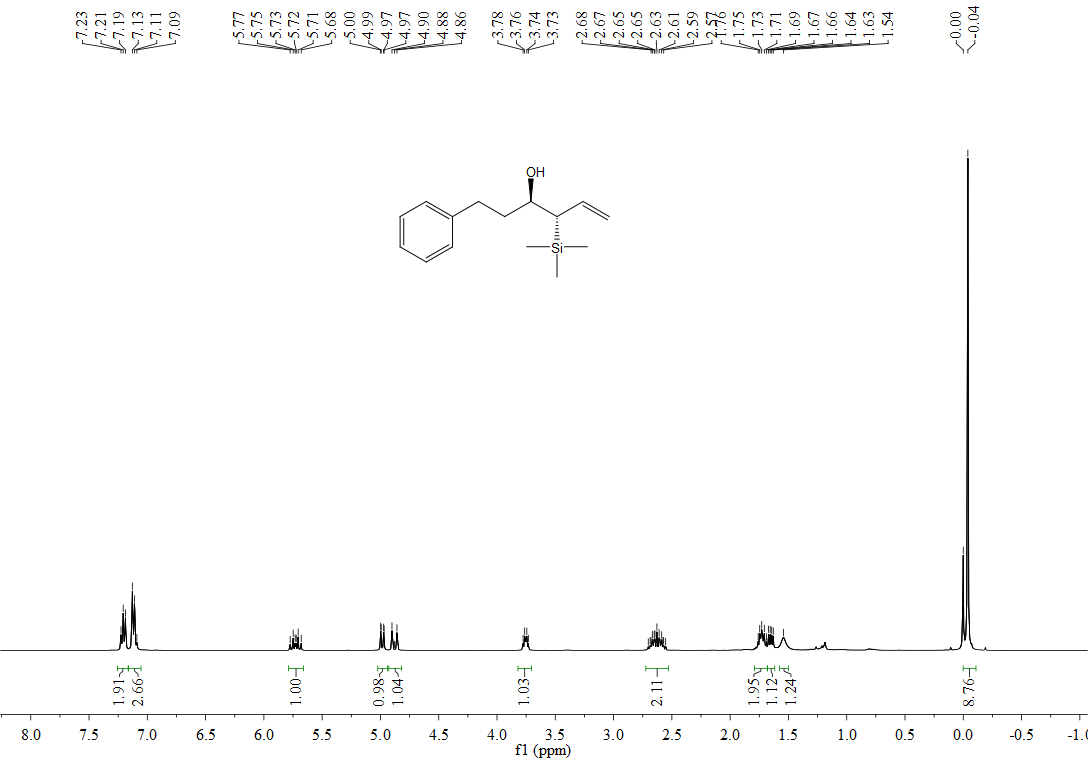


**^13^C NMR (101 MHz, CDCl_3_) (3R,4S)-1-phenyl-4-(trimethylsilyl)hex-5-en-3-ol (2a)**


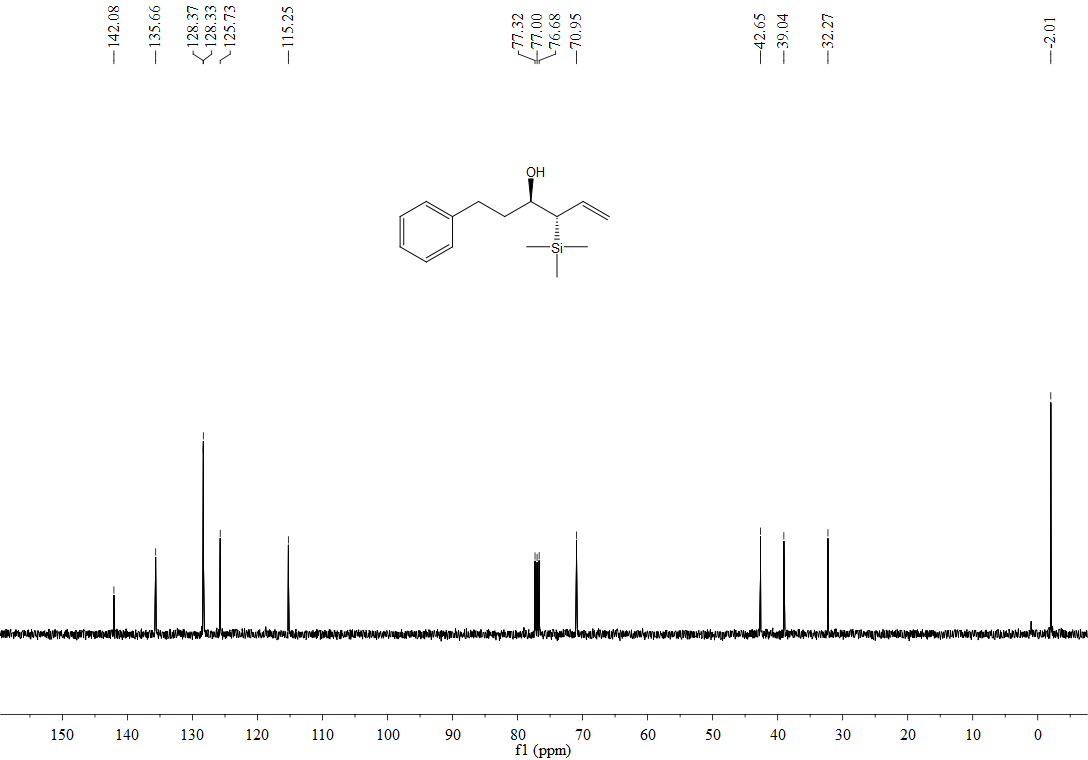


**HPLC (3R,4S)-1-phenyl-4-(trimethylsilyl)hex-5-en-3-ol (2a, Racemic)**


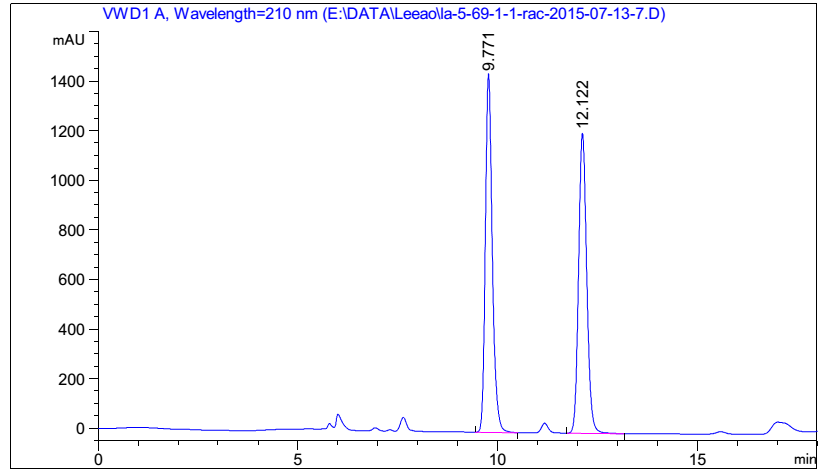


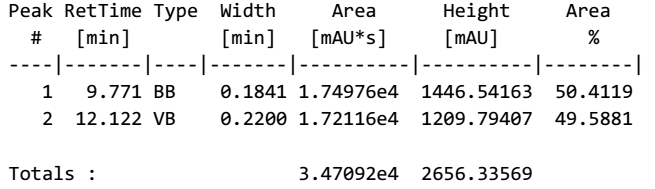


**HPLC (3R,4S)-1-phenyl-4-(trimethylsilyl)hex-5-en-3-ol (2a, 95%ee)**


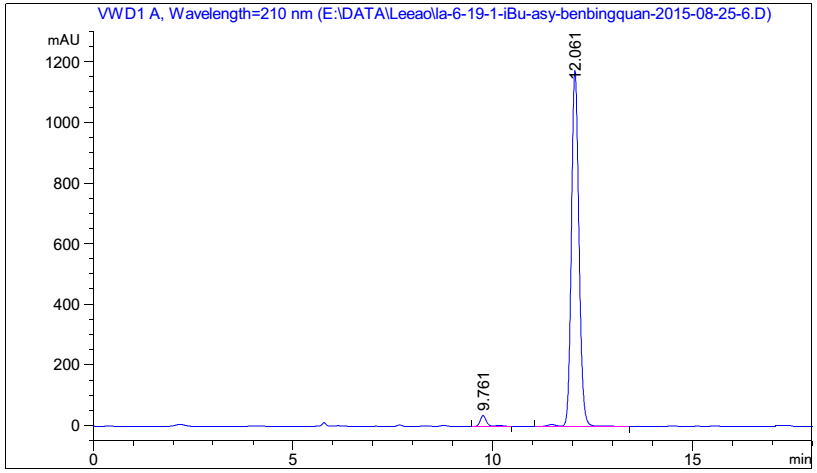


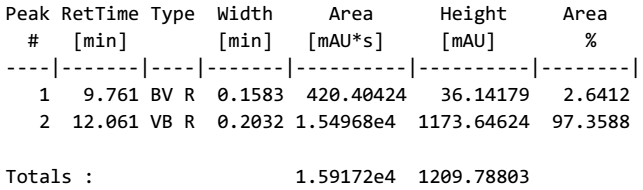


**^1^H NMR (400 MHz, CDCl_3_) (3S,4R)-3-(trimethylsilyl)dec-1-en-4-ol (2b)**


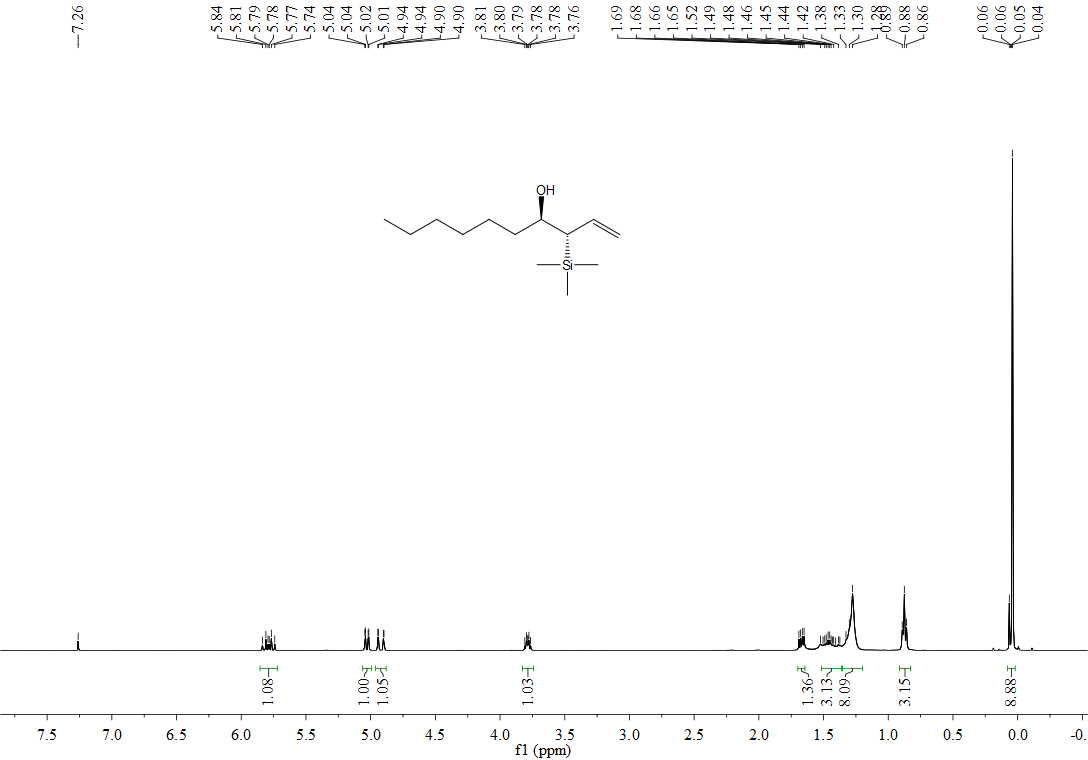


**^13^C NMR (101 MHz, CDCl_3_) (3S,4R)-3-(trimethylsilyl)dec-1-en-4-ol (2b)**


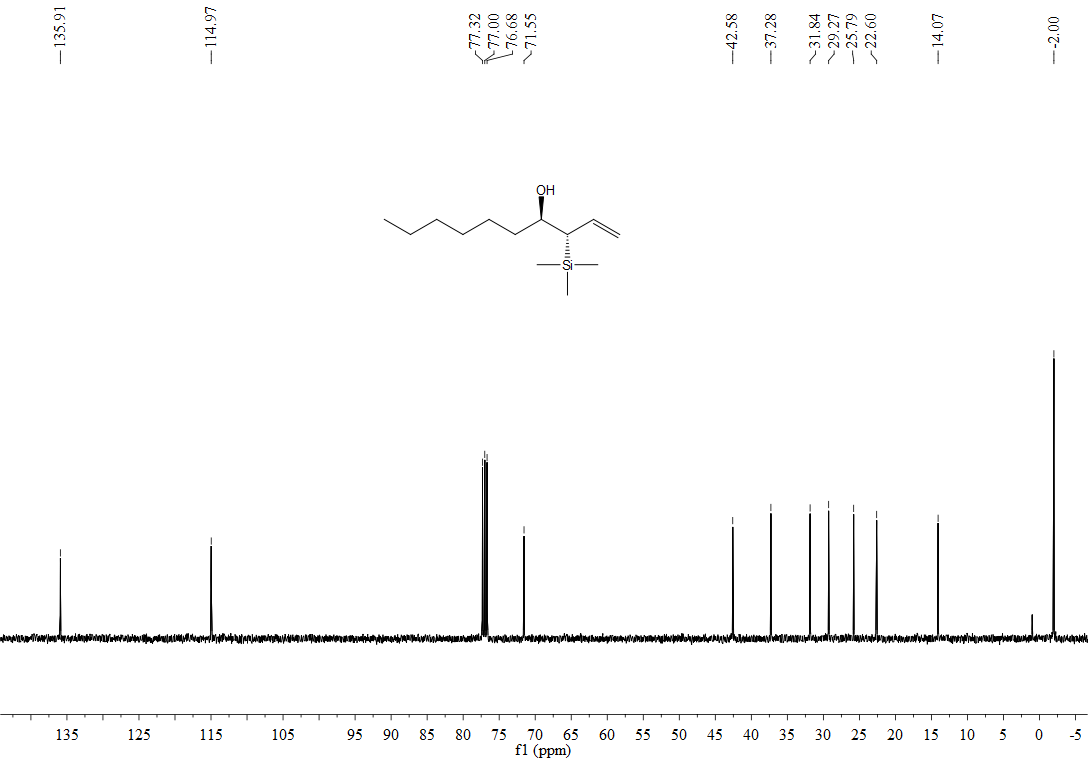


**HPLC (3S,4R)-3-(trimethylsilyl)dec-1-en-4-ol (2b, Racemic)**


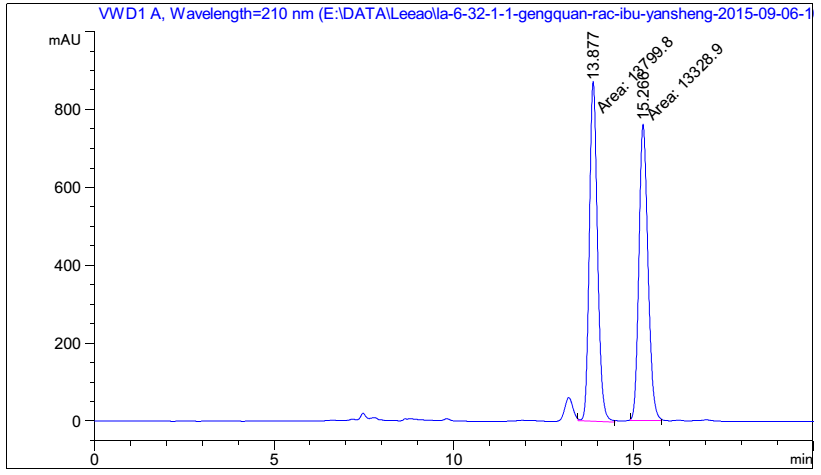


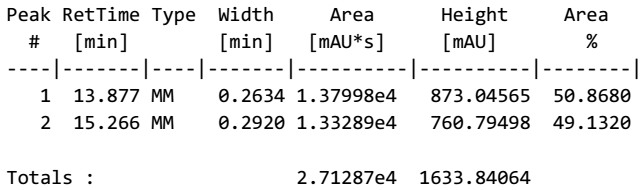


**HPLC (3S,4R)-3-(trimethylsilyl)dec-1-en-4-ol (2b, 96%ee)**


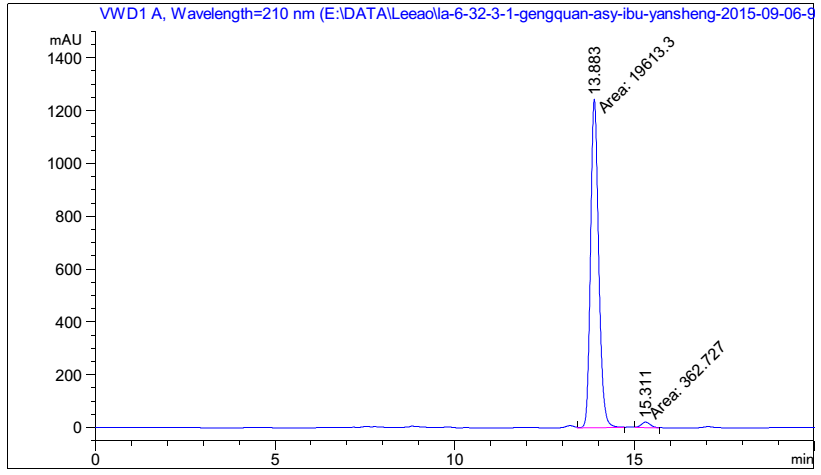


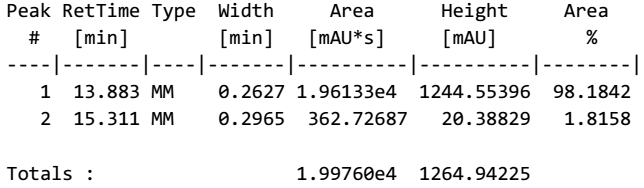


**^1^H NMR (400 MHz, CDCl_3_)** **(1R,2S)-1-cyclohexyl-2-(trimethylsilyl)but-3-en-1-ol (2c)**


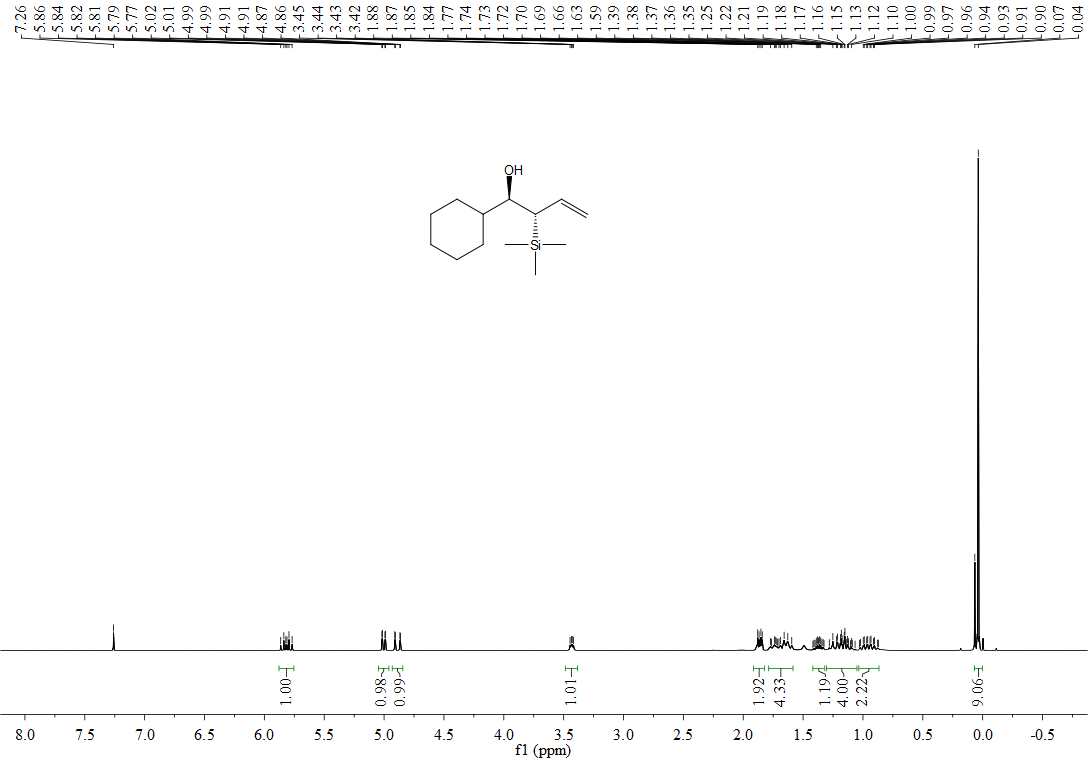


**^13^C NMR (101 MHz, CDCl_3_)** **(1R,2S)-1-cyclohexyl-2-(trimethylsilyl)but-3-en-1-ol (2c)**


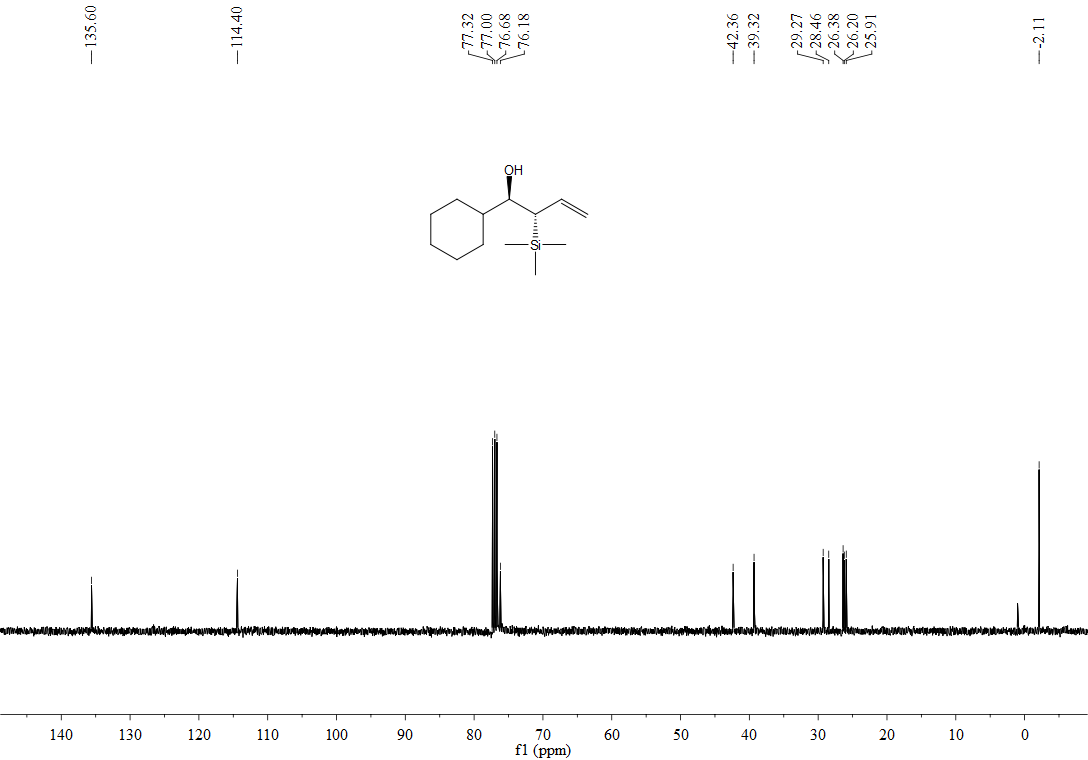


**HPLC (1R,2S)-1-cyclohexyl-2-(trimethylsilyl)but-3-en-1-ol (2c, Racemic)**


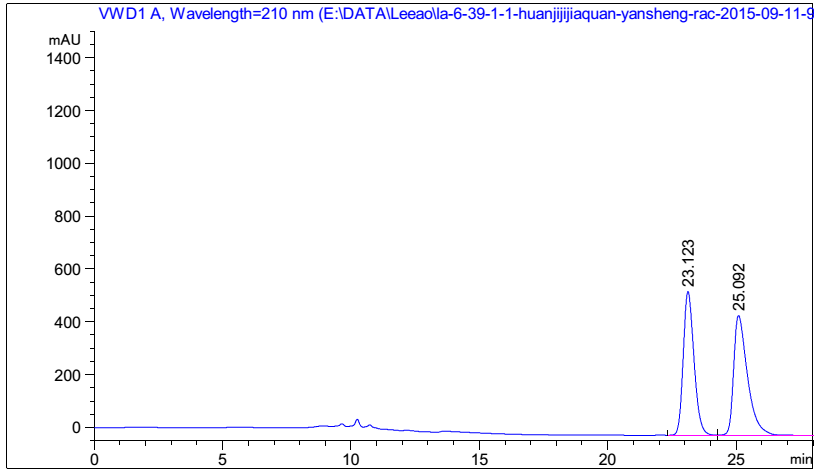


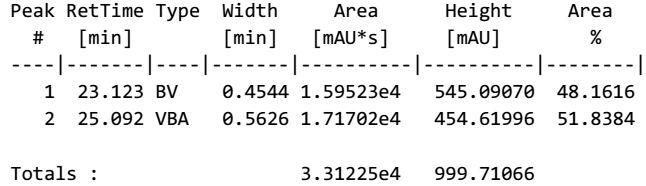


**HPLC (1R,2S)-1-cyclohexyl-2-(trimethylsilyl)but-3-en-1-ol (2c, 92%ee)**


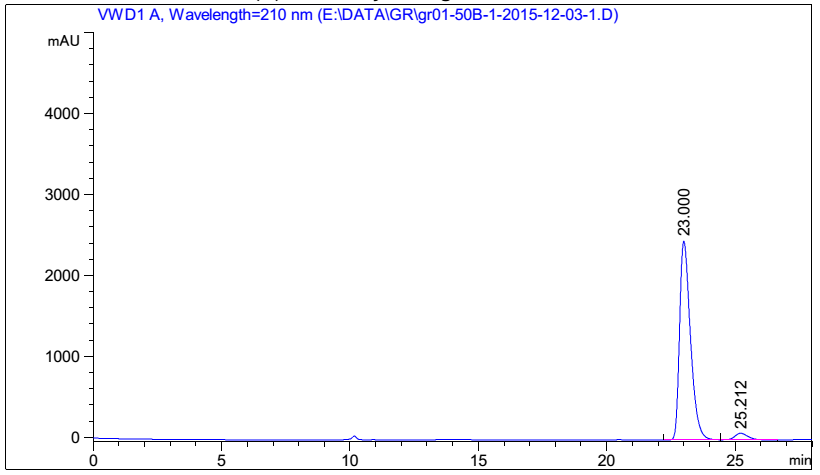


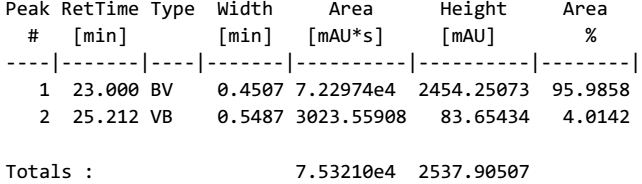


**^1^H NMR (400 MHz, CDCl_3_)** **(3S,4R)-8-chloro-3-(trimethylsilyl)oct-1-en-4-ol (2d)**


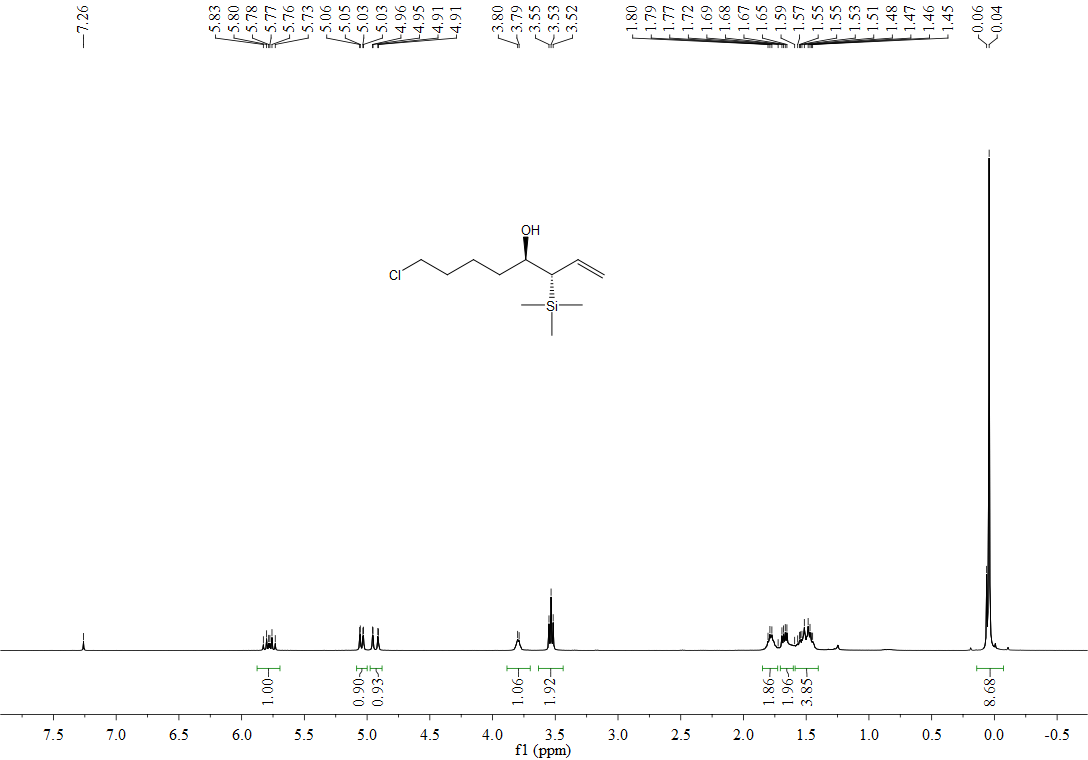


**^13^C NMR (101 MHz, CDCl_3_)** **(3S,4R)-8-chloro-3-(trimethylsilyl)oct-1-en-4-ol (2d)**


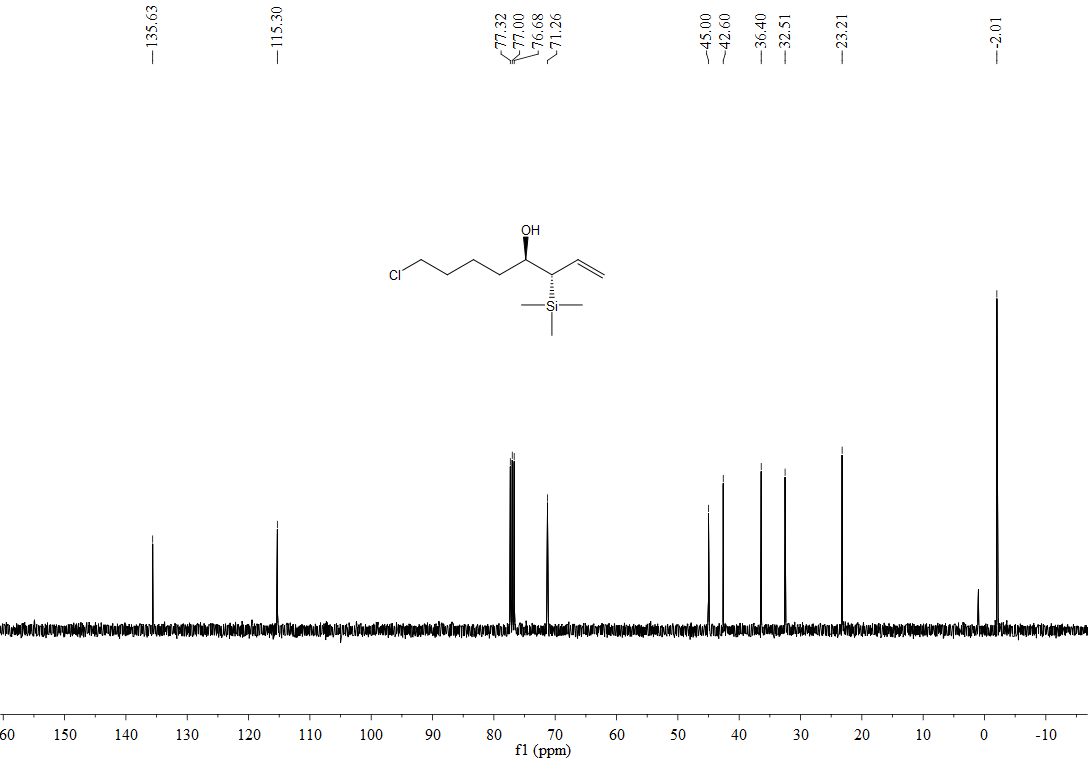


**HPLC (3S,4R)-8-chloro-3-(trimethylsilyl)oct-1-en-4-ol (2d, Racemic)**


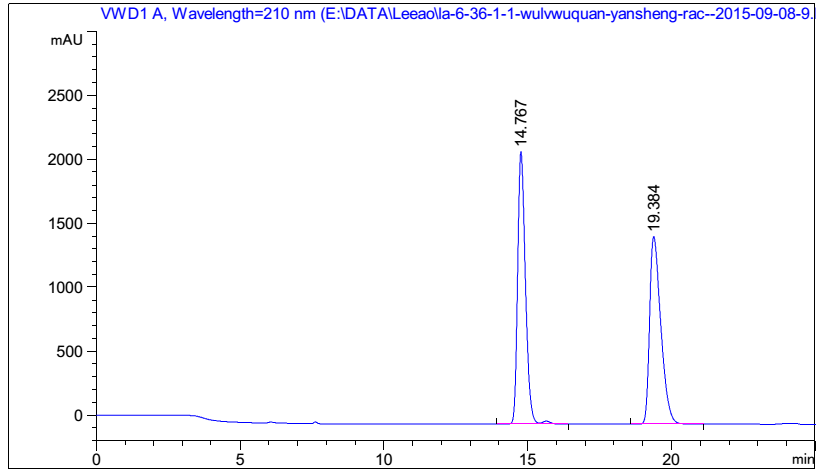


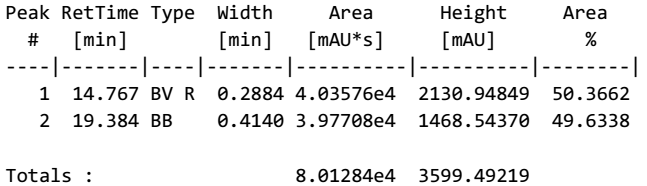


**HPLC (3S,4R)-8-chloro-3-(trimethylsilyl)oct-1-en-4-ol (2d, 97%ee)**


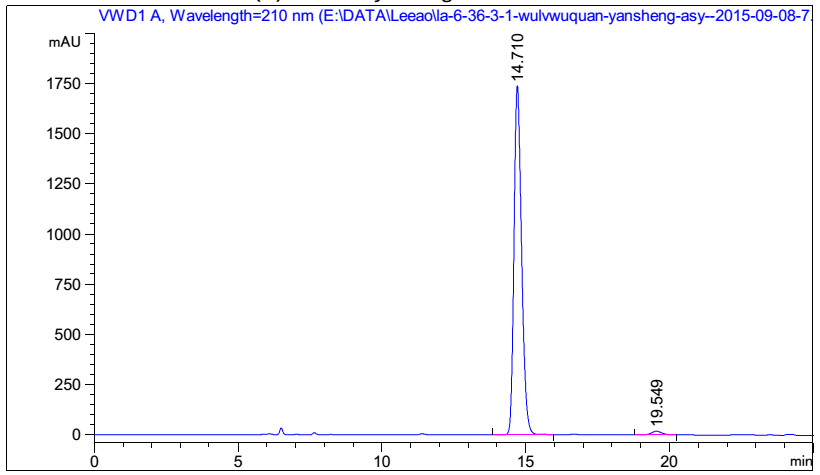


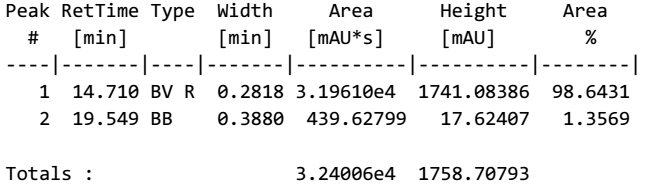


**^1^HNMR (400MHz,CDCl_3_) (3S,4R)-3-(trimethylsilyl)octa-1,7-dien-4-ol (2e)**

**
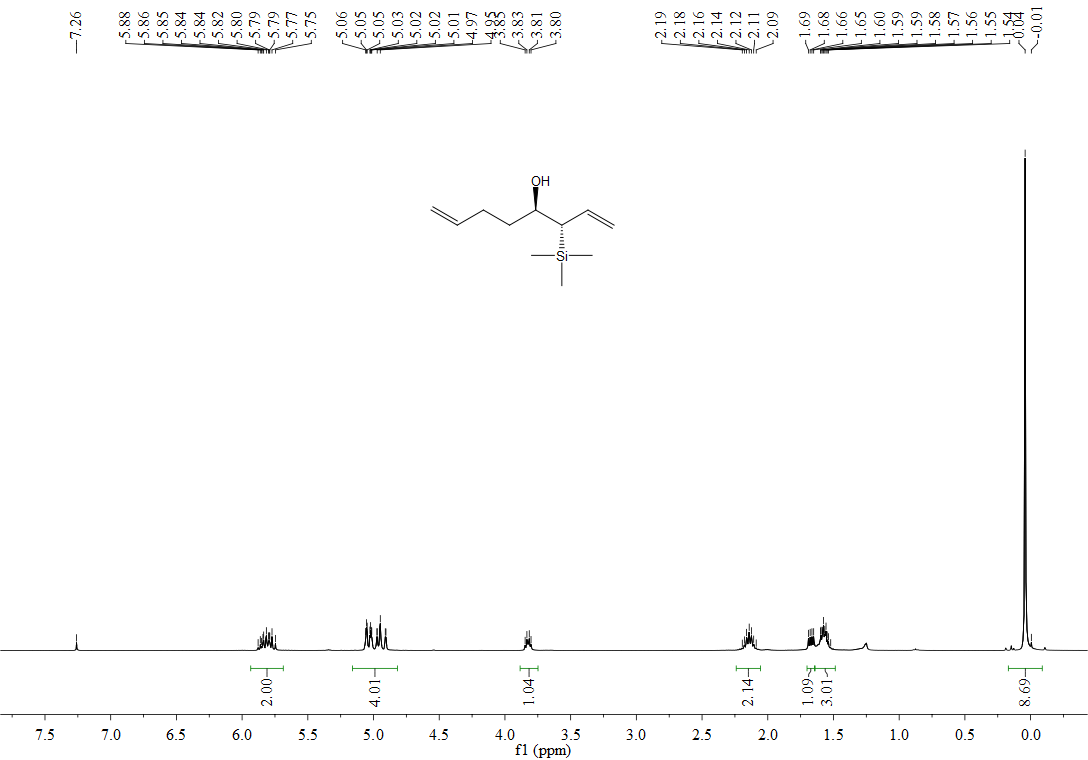
**

**^13^C NMR (101 MHz, CDCl_3_) (3S,4R)-3-(trimethylsilyl)octa-1,7-dien-4-ol (2e)**


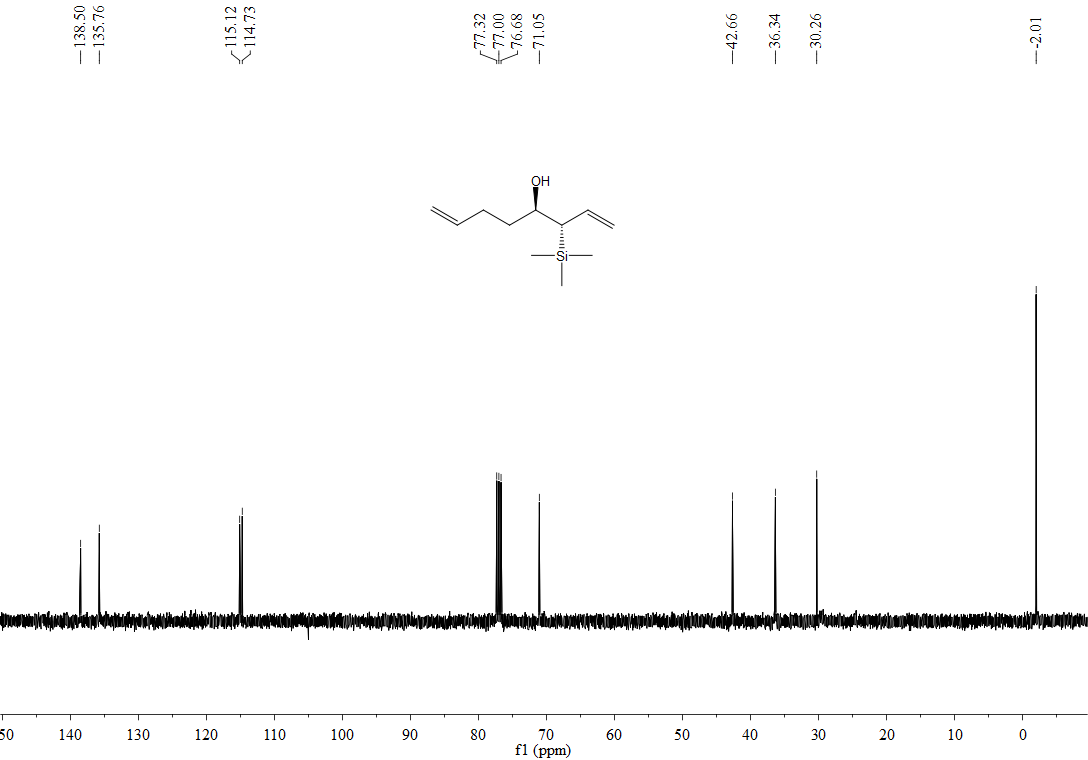


**HPLC (3S,4R)-6,8,8-trimethyl-3-(trimethylsilyl)non-1-en-4-ol (2e, Racemic)**


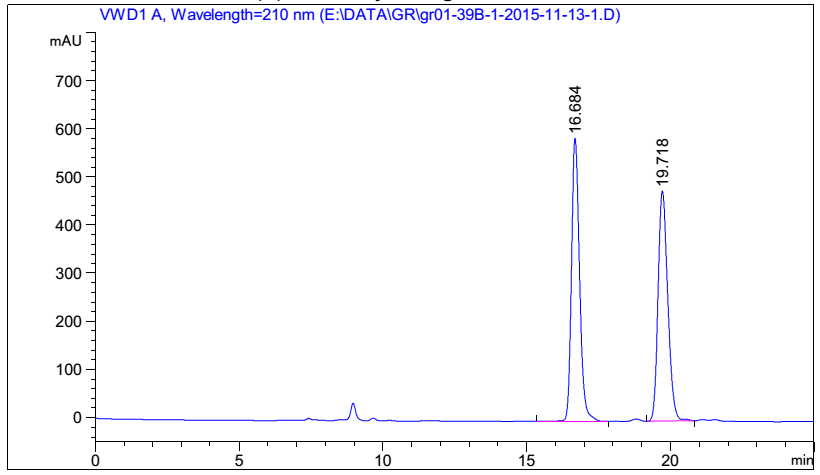


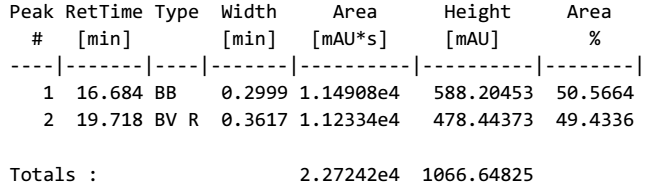


**HPLC (3S,4R)-6,8,8-trimethyl-3-(trimethylsilyl)non-1-en-4-ol (2e, 97%ee)**


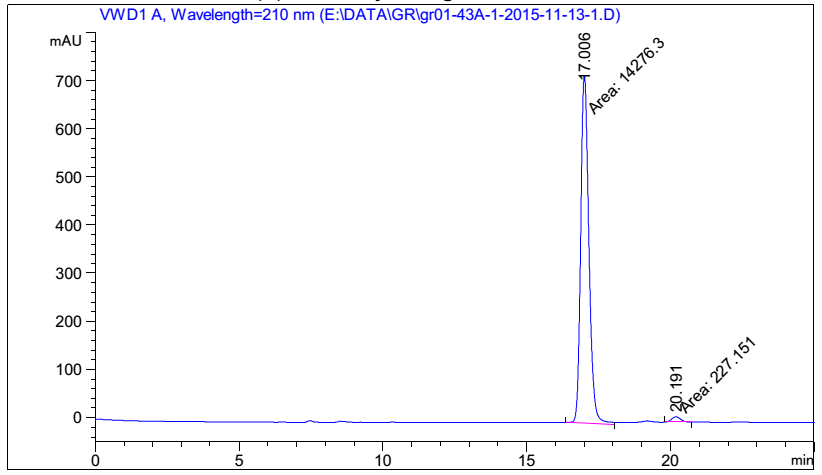


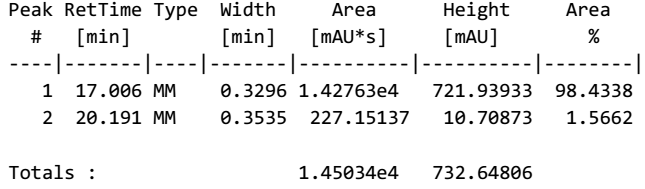


**^1^H NMR (400MHz,CDCl_3_) (1R,2S)-1-(cyclohex-3-en-1-yl)-2-(trimethylsilyl)**

**but-3-en-1-ol (2f)**

**
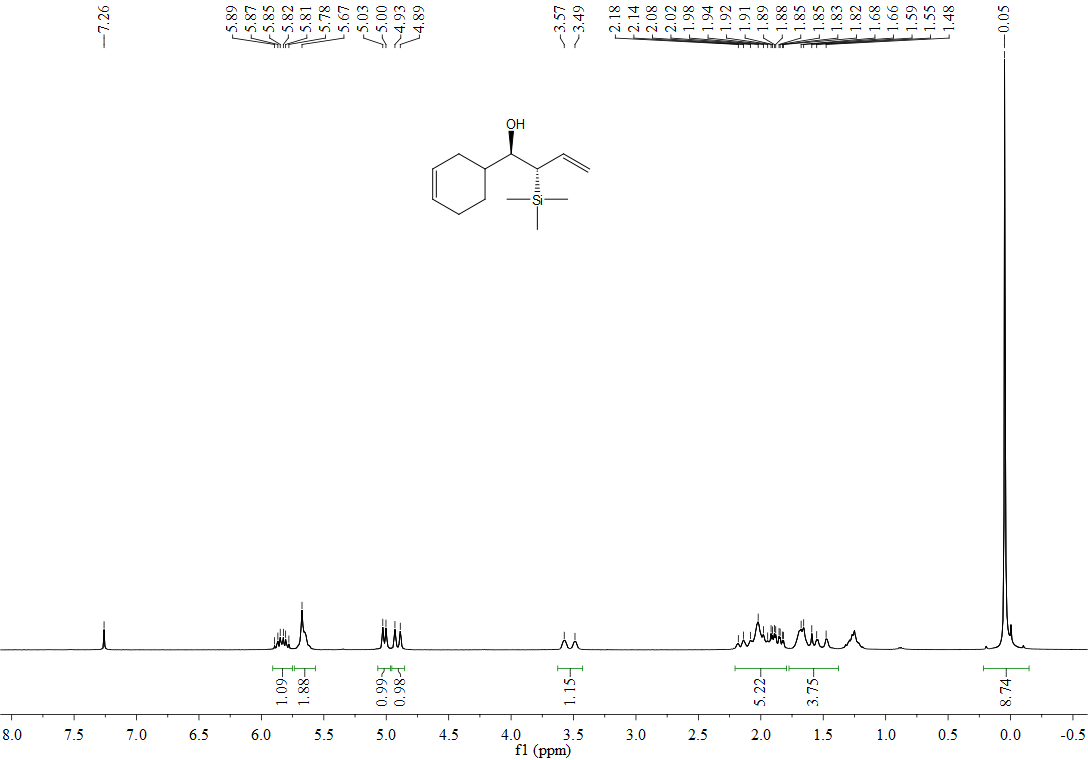
**

**^13^C NMR (101MHz,CDCl_3_) (1R,2S)-1-(cyclohex-3-en-1-yl)-2-(trimethylsilyl)**

**but-3-en-1-ol (2f)**


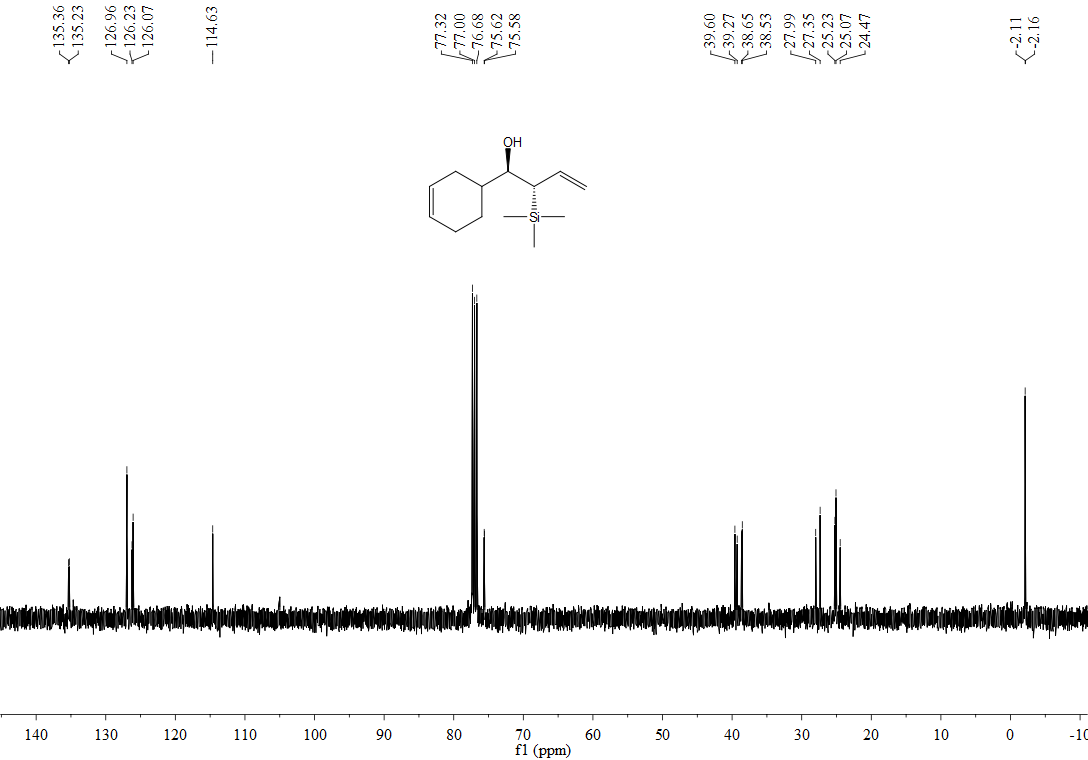


**HPLC (1R,2S)-1-(cyclohex-3-en-1-yl)-2-(trimethylsilyl)but-3-en-1-ol (2f, Racemic)**


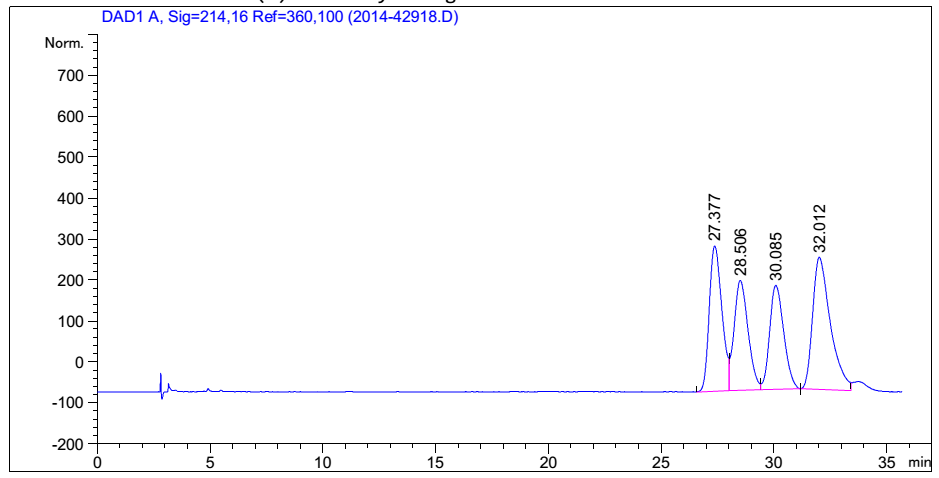


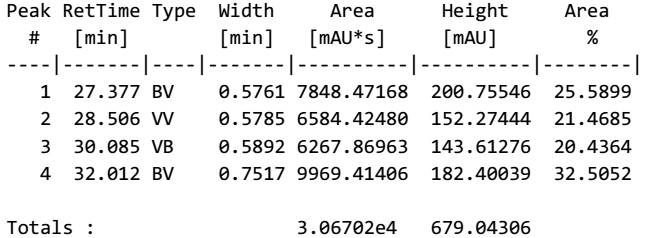


**HPLC (1R,2S)-1-(cyclohex-3-en-1-yl)-2-(trimethylsilyl)but-3-en-1-ol (2f, 90% de)**


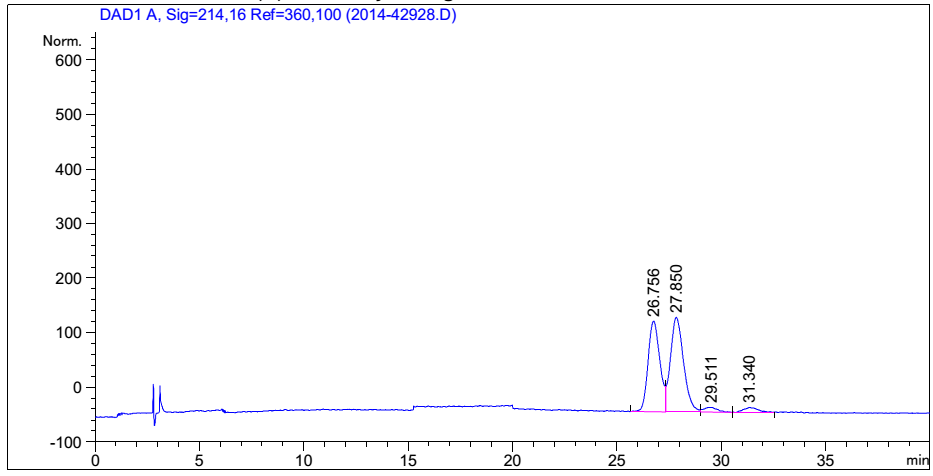


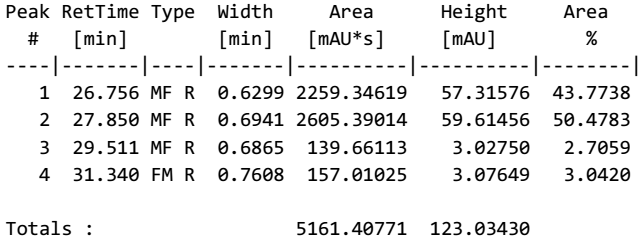


**^1^H NMR (400MHz,CDCl_3_) (3R,4S)-1-((tert-butyldiphenylsilyl)oxy)-4-(trimethylsilyl)**

**hex-5-en-3-ol (2g)**

**
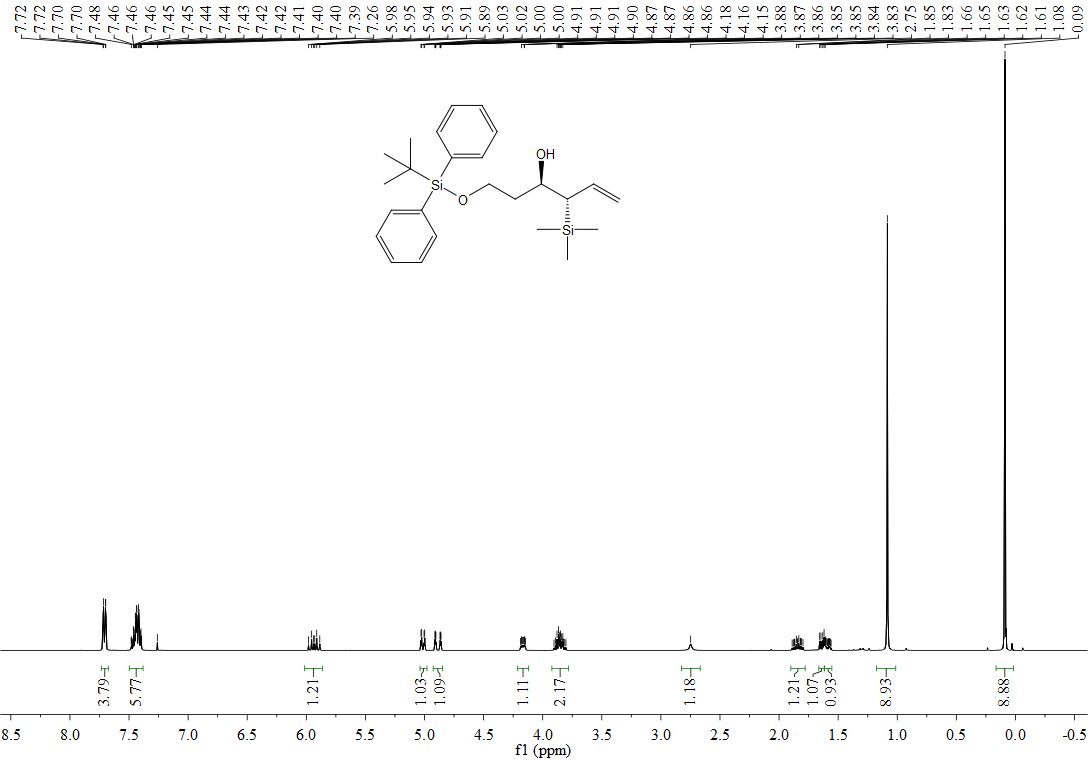
**

**^13^C NMR (101MHz,CDCl_3_) (3R,4S)-1-((tert-butyldiphenylsilyl)oxy)-4-(trimethylsilyl)**

**hex-5-en-3-ol (2g)**


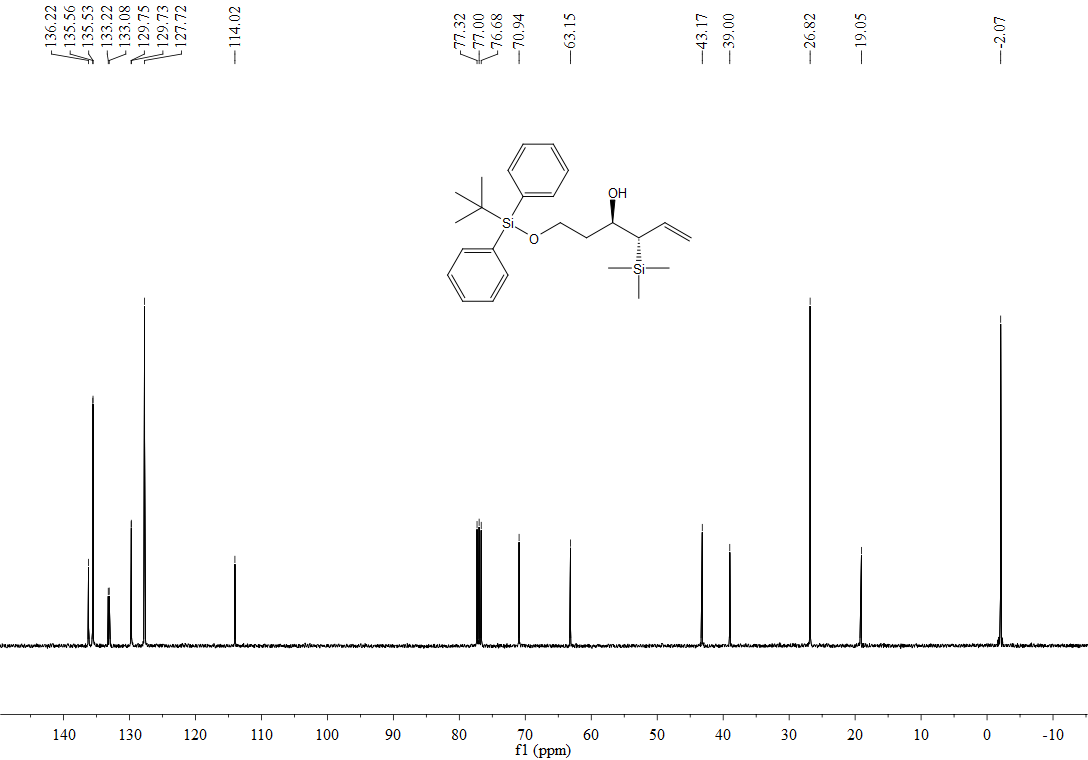


**HPLC (3R,4S)-1-((tert-butyldiphenylsilyl)oxy)-4-(trimethylsilyl) hex-5-en-3-ol**

**(2g, Racemic)**


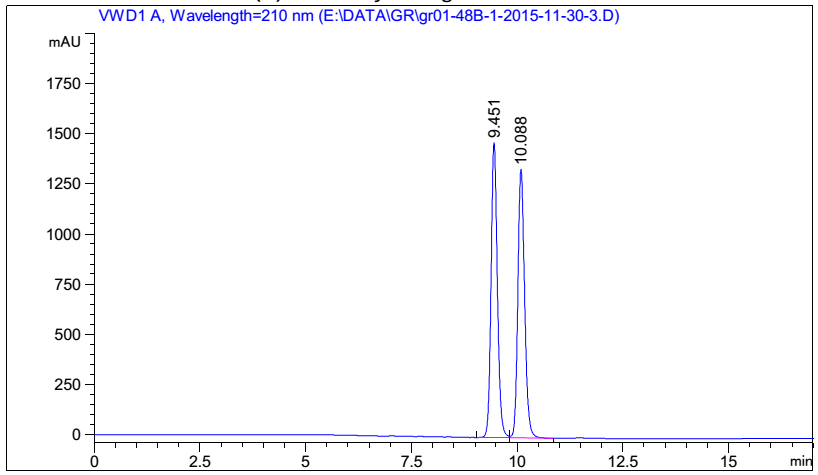


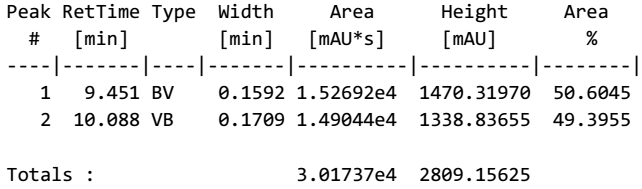


**HPLC (3R,4S)-1-((tert-butyldiphenylsilyl)oxy)-4-(trimethylsilyl) hex-5-en-3-ol**

**(2g, 97% ee)**


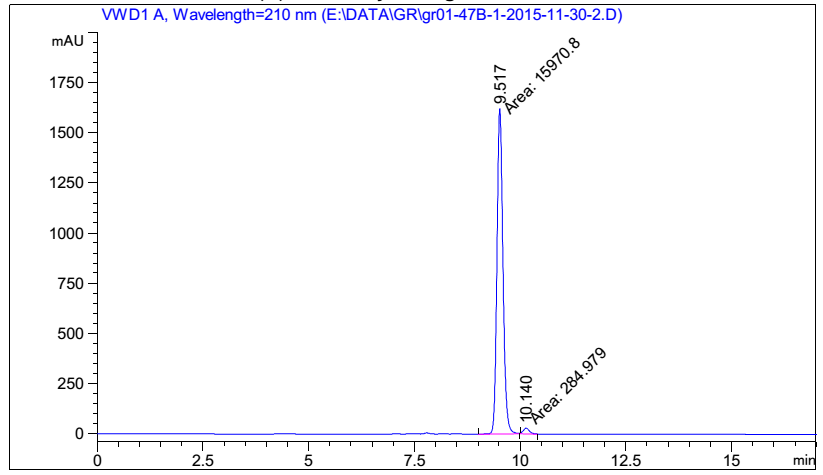


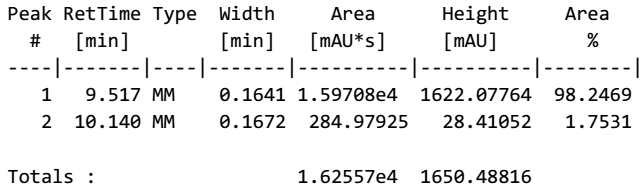


**^1^H NMR (400MHz,CDCl_3_) 2-((3R,4S)-3-hydroxy-4-(trimethylsilyl)hex-5-en-1-yl)**

**isoindoline-1,3-dione (2h)**

**
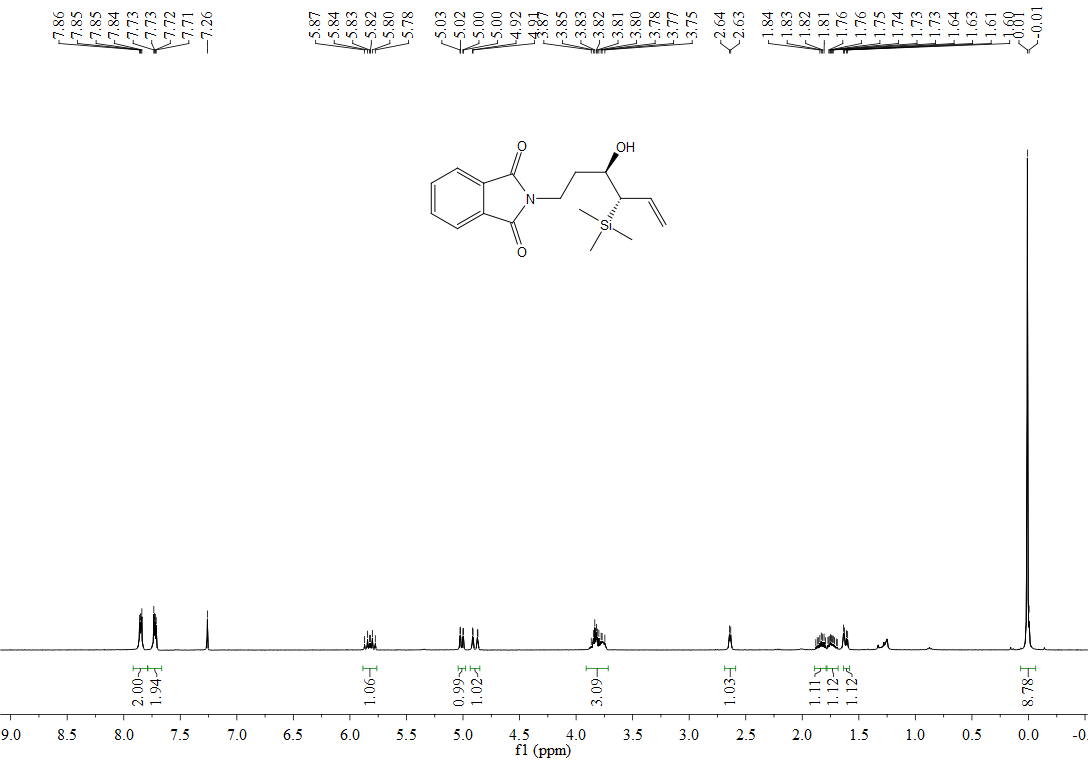
**

**^13^C NMR (101MHz,CDCl_3_) 2-((3R,4S)-3-hydroxy-4-(trimethylsilyl)hex-5-en-1-yl)**

**isoindoline-1,3-dione (2h)**


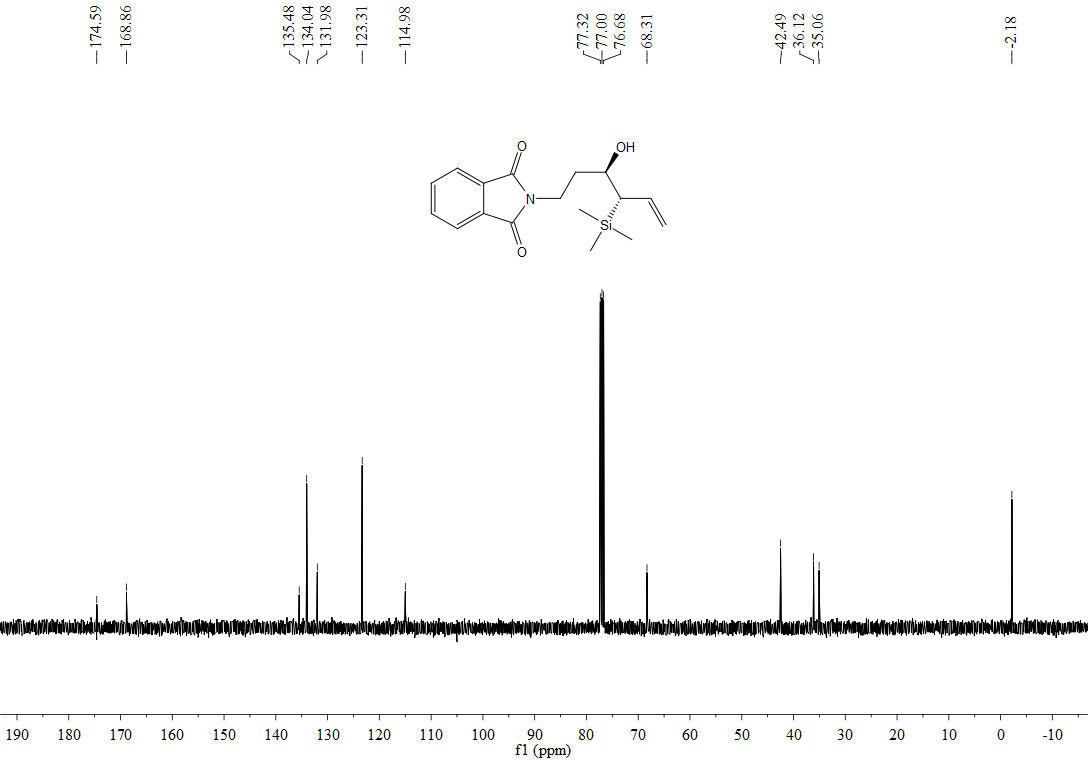


**HPLC 2-((3R,4S)-3-hydroxy-4-(trimethylsilyl)hex-5-en-1-yl)isoindoline-1,3-dione**

**(2h, Racemic)**


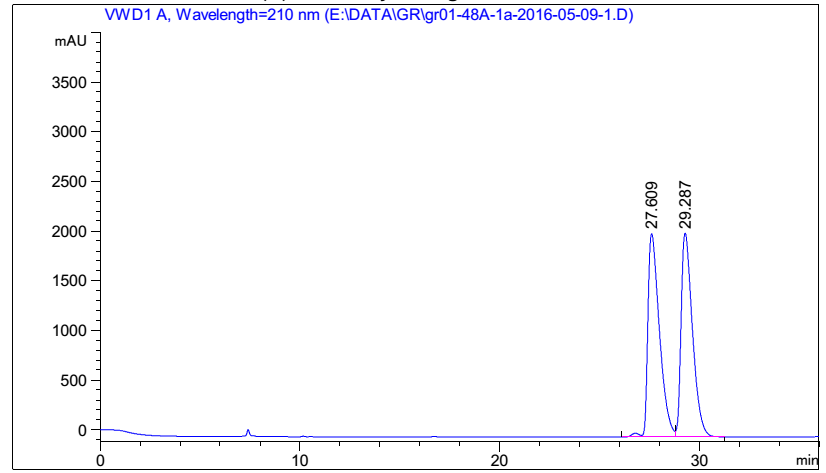


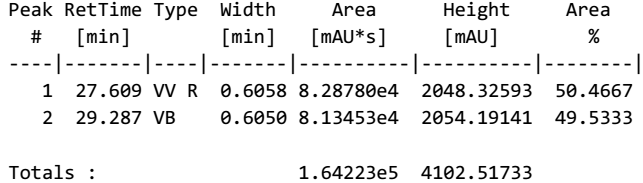


**HPLC2-((3R,4S)-3-hydroxy-4-(trimethylsilyl)hex-5-en-1-yl)isoindoline-1,3-dione**

**(2h, 98% ee)**


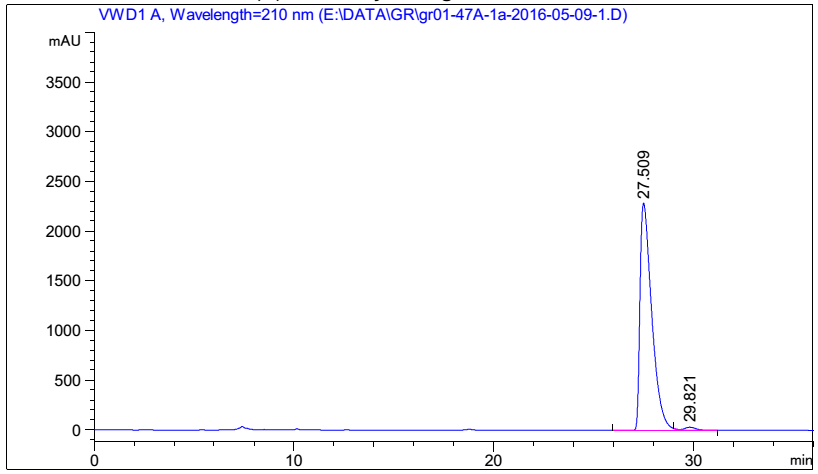


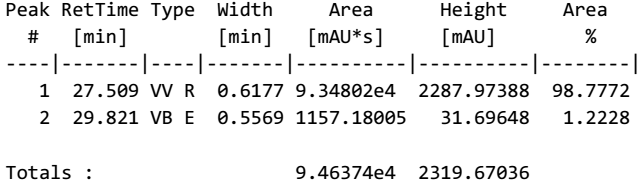


**^1^HNMR (400MHz,CDCl_3_) (2R,3S)-1-(2,2-dimethyl-1,3-dioxolan-4-yl)-3-(trimethylsilyl)**

**pent-4-en-2-ol (2i)- minor**

**
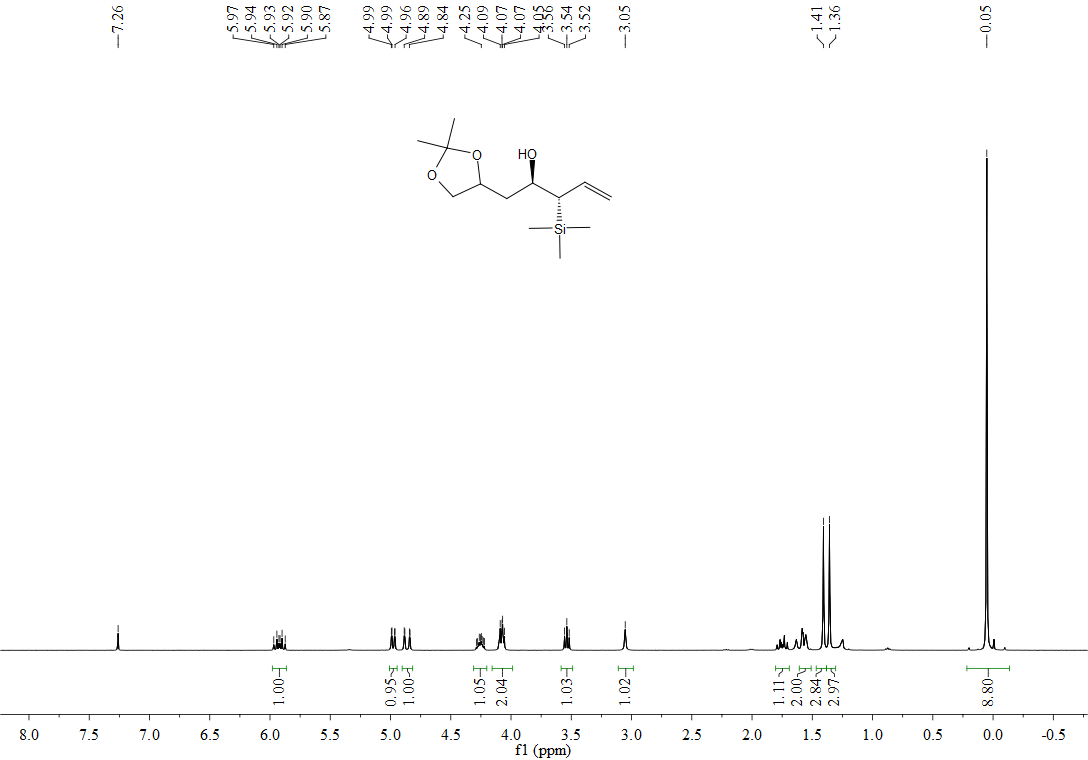
**

**^13^C NMR (101 MHz,CDCl_3_)(2R,3S)-1-(2,2-dimethyl-1,3-dioxolan-4-yl)-3-(trimethylsilyl)**

**pent-4-en-2-ol (2i)- minor**


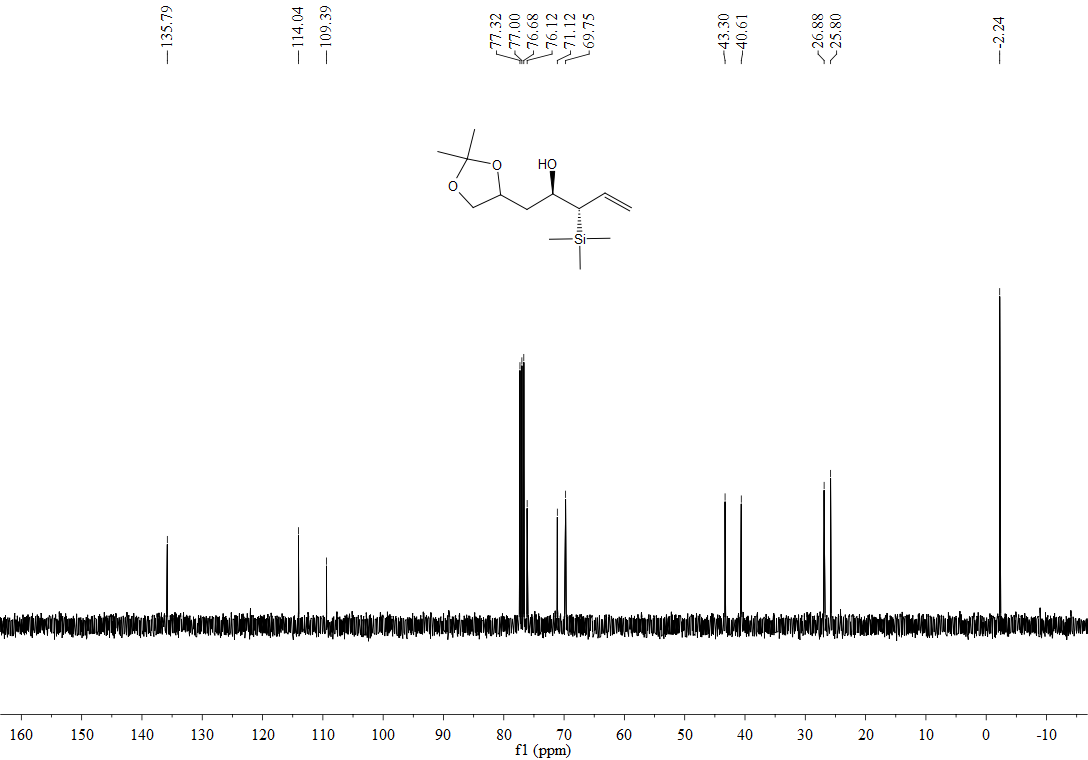


**^1^HNMR (400MHz,CDCl_3_) (2R,3S)-1-(2,2-dimethyl-1,3-dioxolan-4-yl)-3-(trimethylsilyl)**

**pent-4-en-2-ol (2i)- major**

**
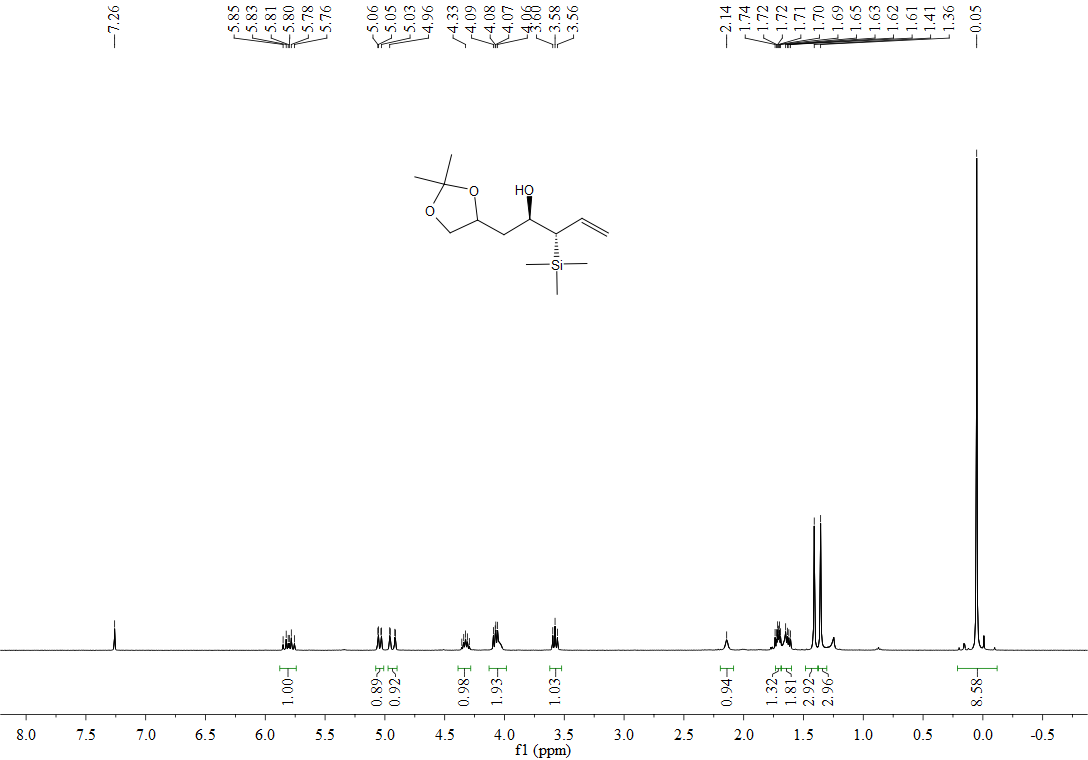
**

**^13^C NMR (101 MHz,CDCl_3_)(2R,3S)-1-(2,2-dimethyl-1,3-dioxolan-4-yl)-3-(trimethylsilyl)**

**pent-4-en-2-ol (2i)-major**


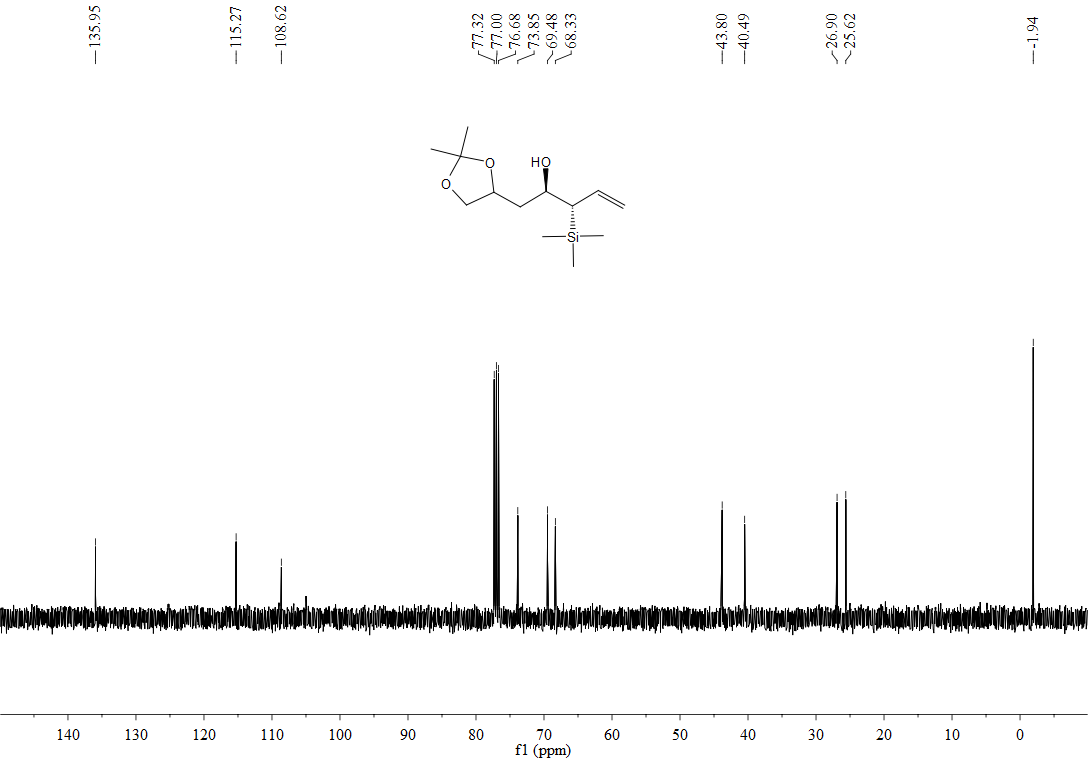


**HPLC (2R,3S)-1-(2,2-dimethyl-1,3-dioxolan-4-yl)-3-(trimethylsilyl)pent-4-en-2-ol**

**(2i, Racemic)**


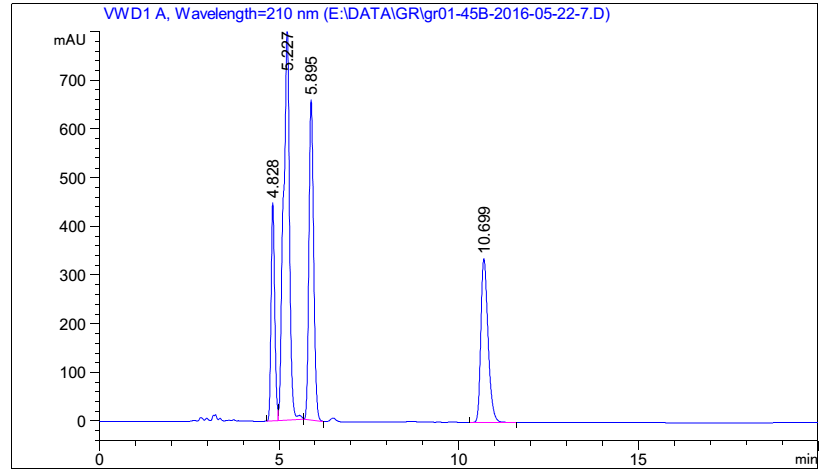


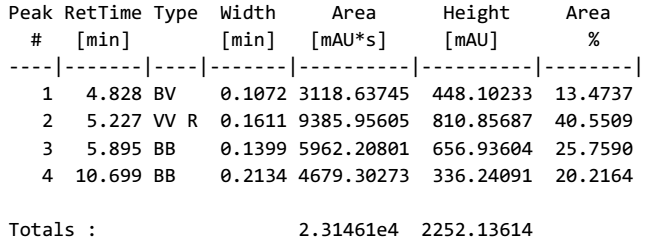


**HPLC (2R,3S)-1-(2,2-dimethyl-1,3-dioxolan-4-yl)-3-(trimethylsilyl)pent-4-en-2-ol**

**(2i, >97% ee)**


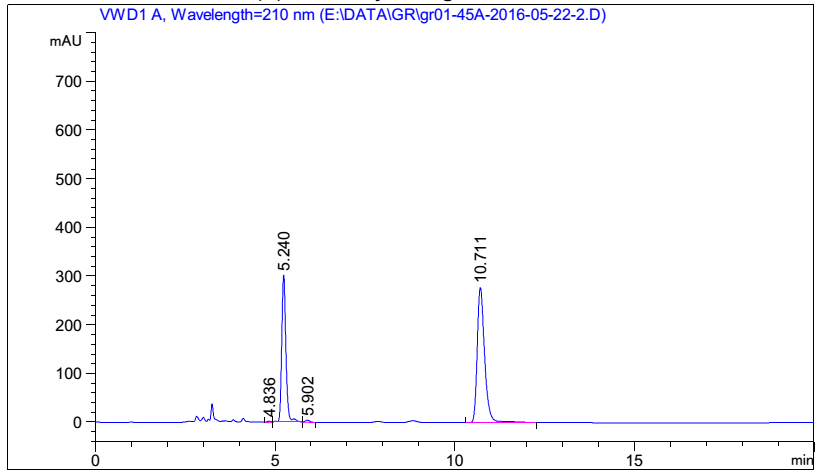


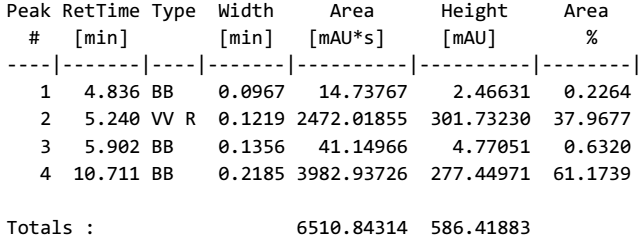


**^1^HNMR (400MHz,CDCl_3_) (3S,4R,6S)-6,10-dimethyl-3-(trimethylsilyl)undeca**

**-1,9-dien-4-ol (2j)**

**
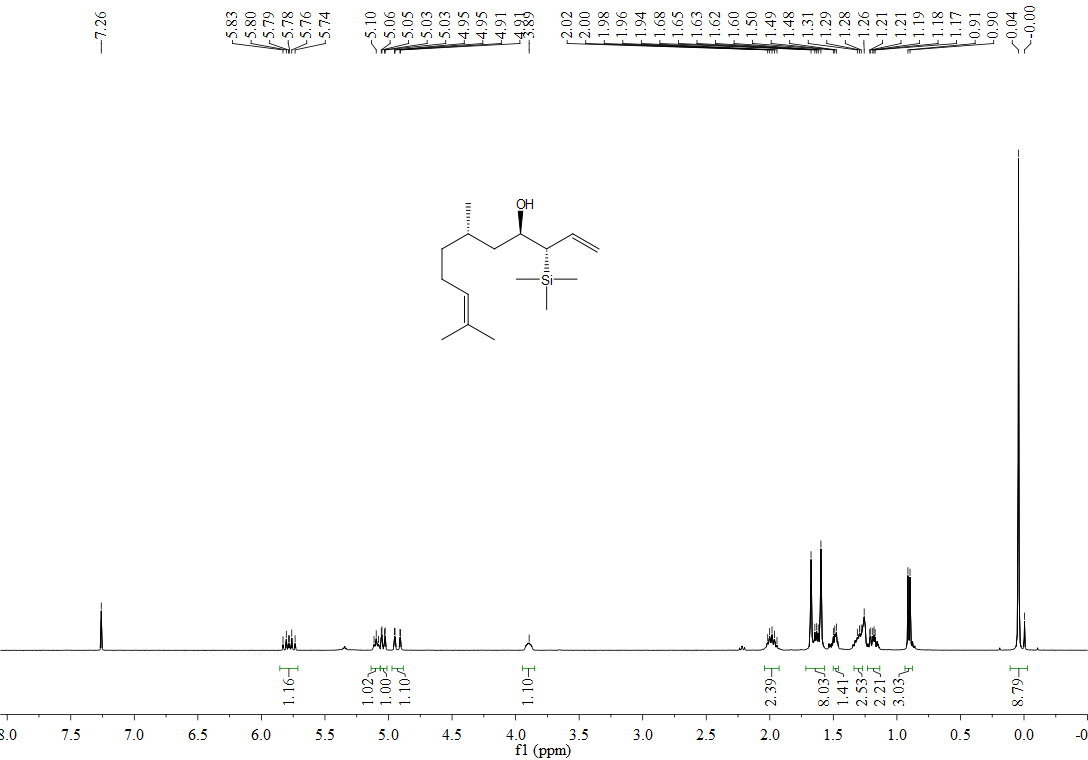
**

**^13^C NMR (101 MHz, CDCl_3_)** **(3S,4R,6S)-6,10-dimethyl-3-(trimethylsilyl)undeca-**

**1,9-dien-4-ol (2j)**


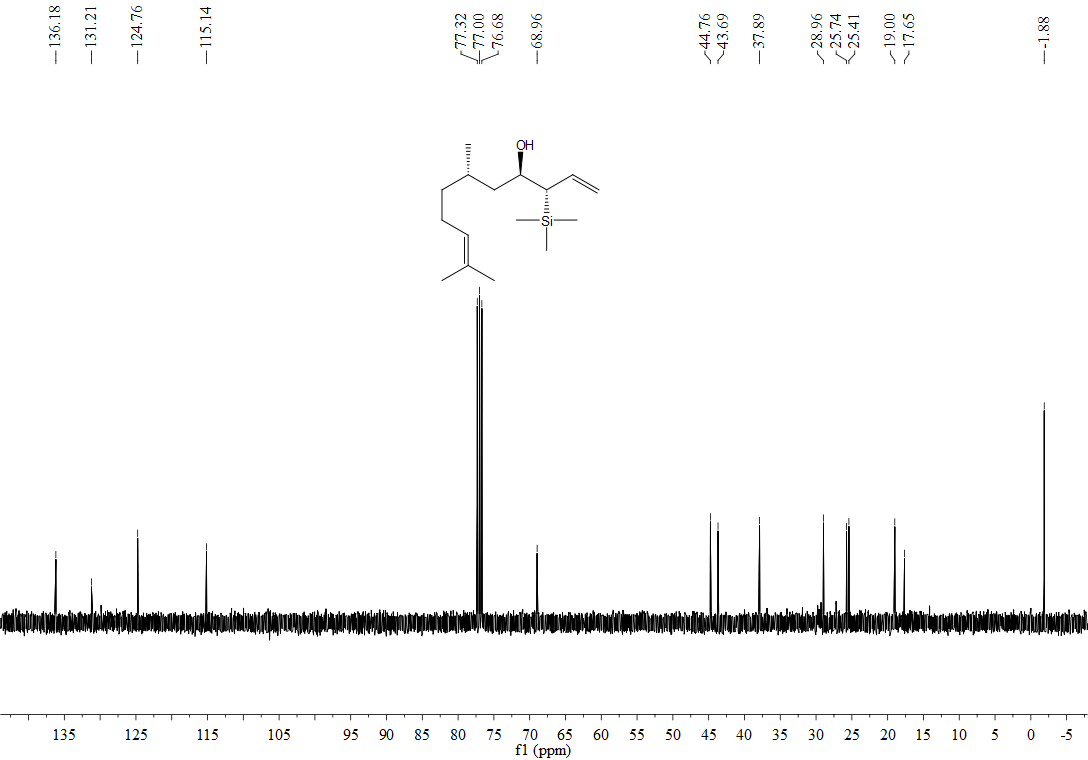


**HPLC (3S,4R,6S)-6,10-dimethyl-3-(trimethylsilyl)undeca-1,9-dien-4-ol (2j, Racemic)**


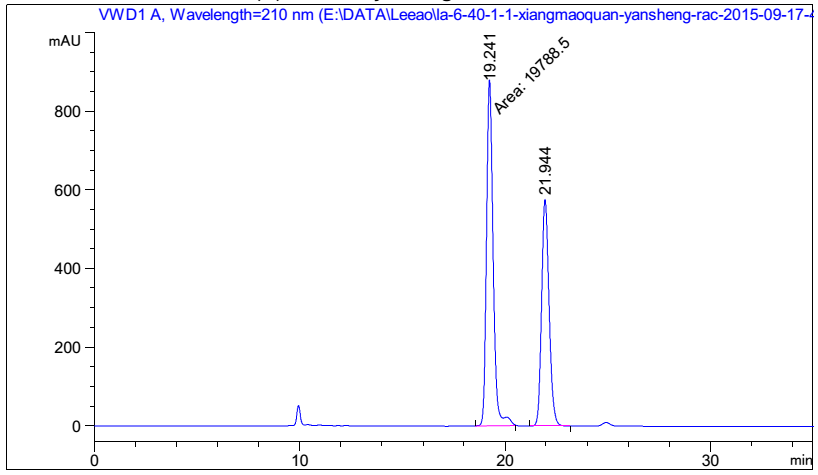


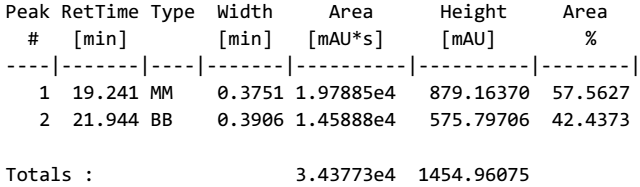


**HPLC (3S,4R,6S)-6,10-dimethyl-3-(trimethylsilyl)undeca-1,9-dien-4-ol (2j, 97%de)**


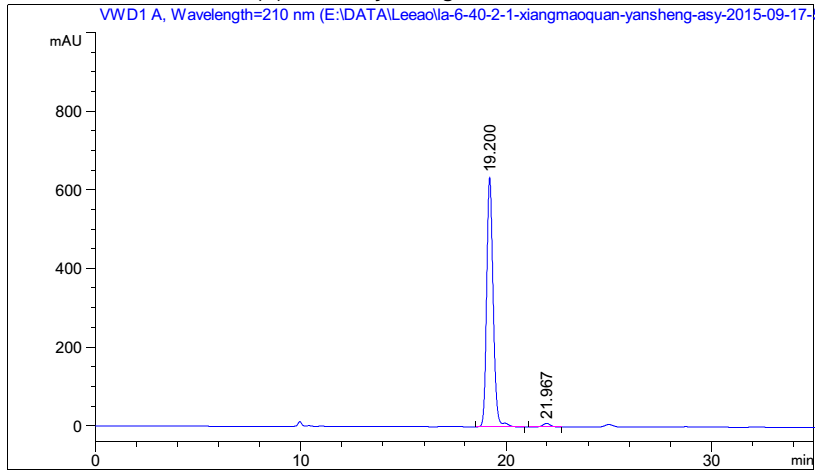


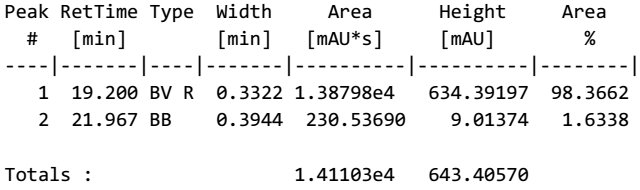


**^1^HNMR (400MHz,CDCl_3_) (3S,4R)-6,8,8-trimethyl-3-(trimethylsilyl)non-1-en-4-ol (2k)**

**
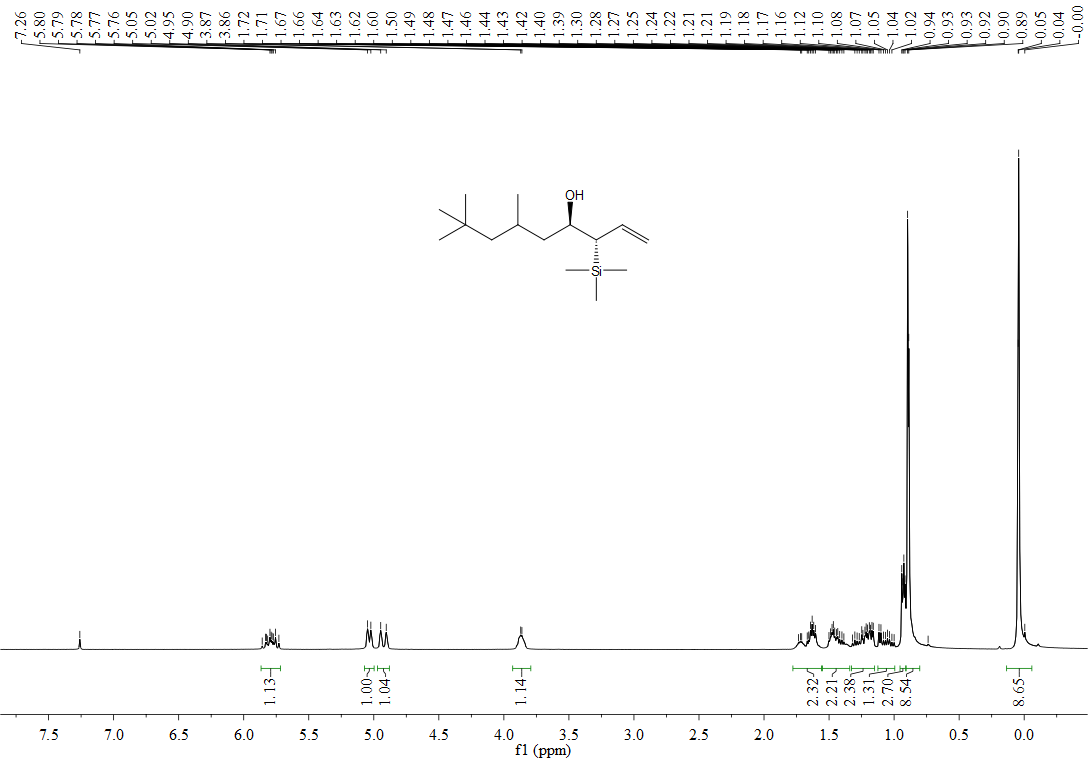
**

**^13^C NMR (101 MHz, CDCl_3_) (3S,4R)-6,8,8-trimethyl-3-(trimethylsilyl)non-1-en-4-ol (2k)**


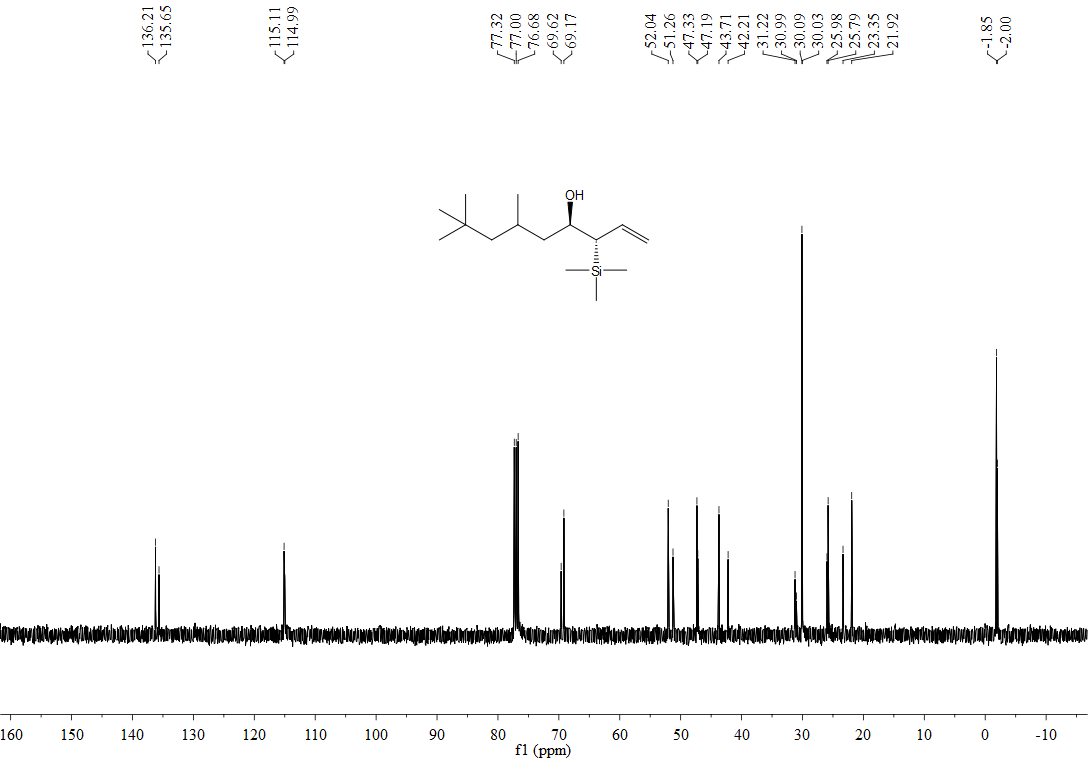


**HPLC (3S,4R)-6,8,8-trimethyl-3-(trimethylsilyl)non-1-en-4-ol (2k, Racemic)**


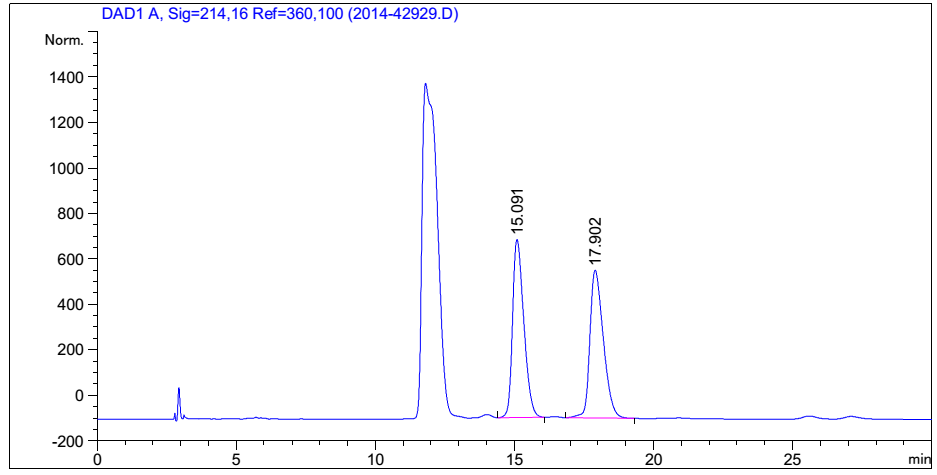


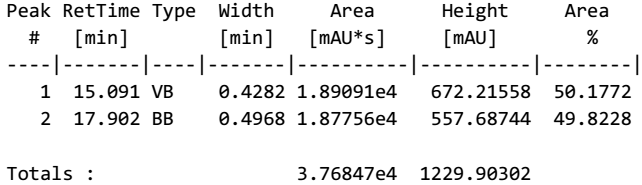


**HPLC (3S,4R)-6,8,8-trimethyl-3-(trimethylsilyl)non-1-en-4-ol (2k, 95%de)**


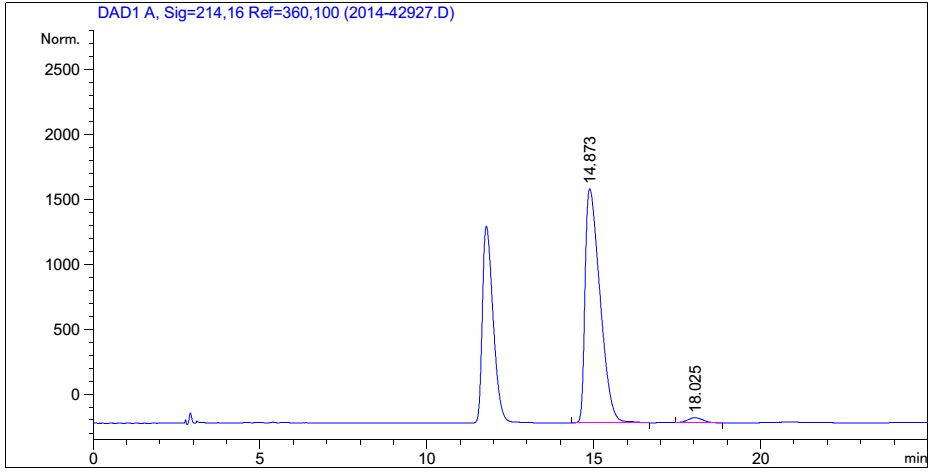


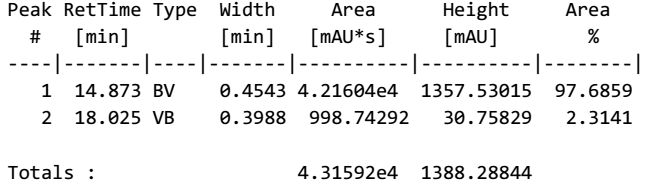


**^1^HNMR (400MHz,CDCl_3_) (1R,2S)-1-phenyl-2-(trimethylsilyl)but-3-en-1-ol (2l)**

**
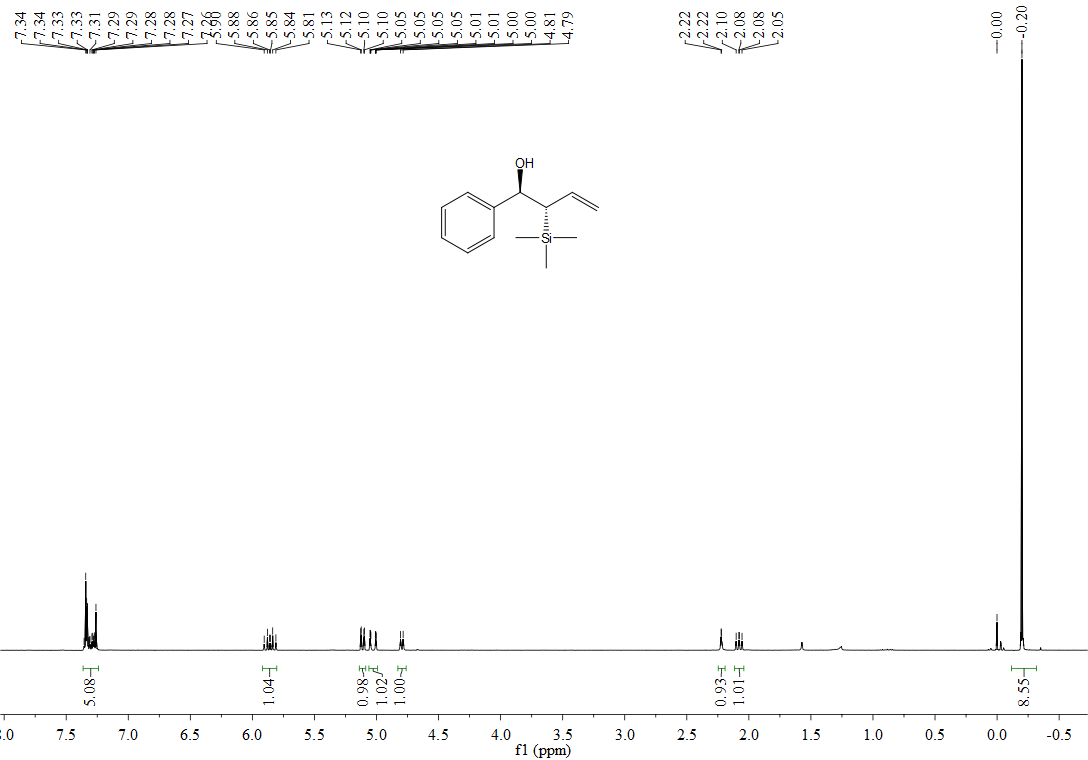
**

**^13^C NMR (101 MHz,CDCl_3_) (1R,2S)-1-phenyl-2-(trimethylsilyl)but-3-en-1-ol (2l)**


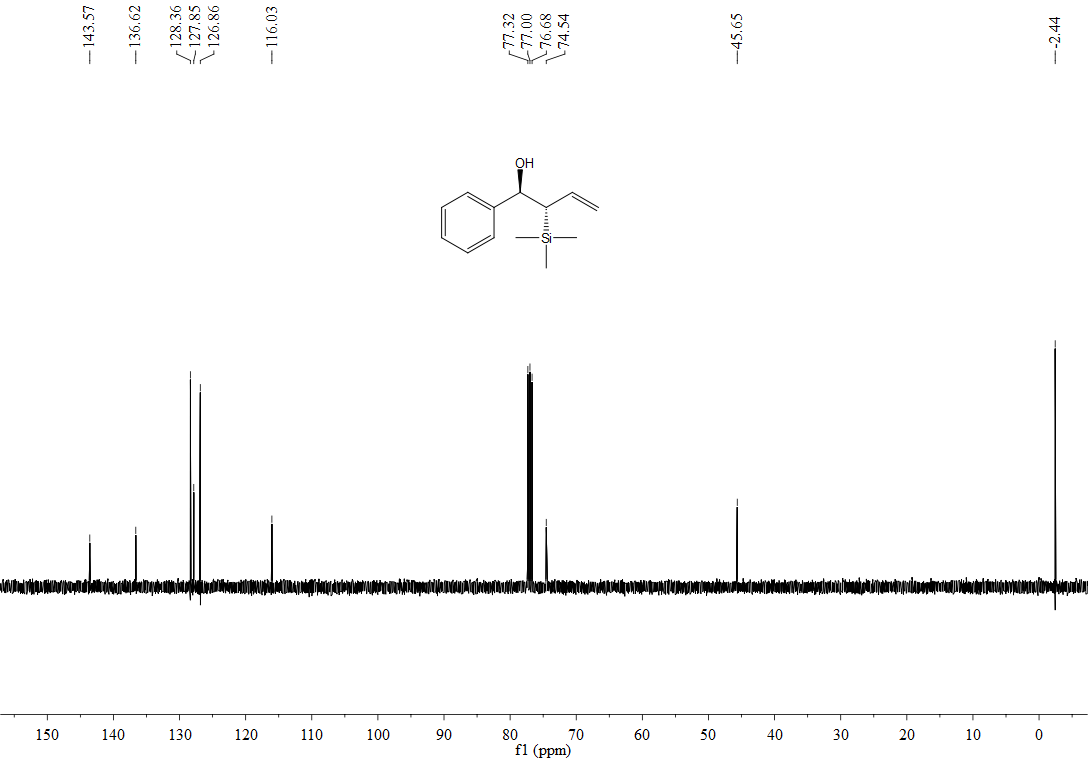


**HPLC (1R,2S)-1-phenyl-2-(trimethylsilyl)but-3-en-1-ol (2l, Racemic)**


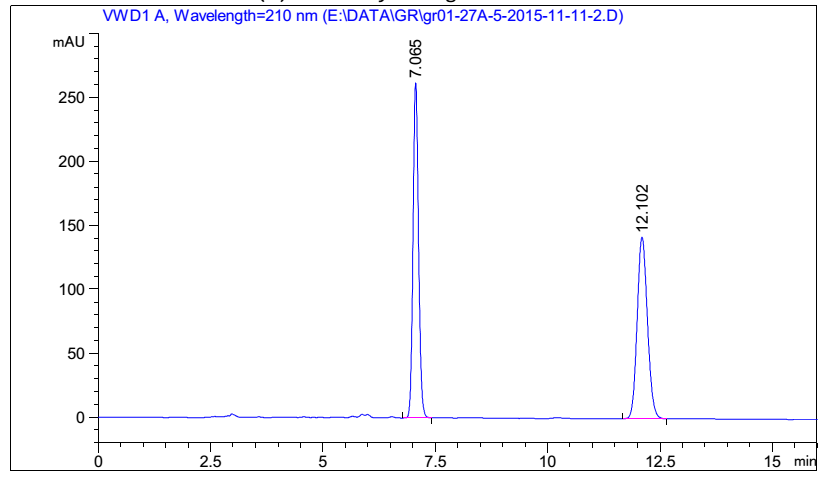


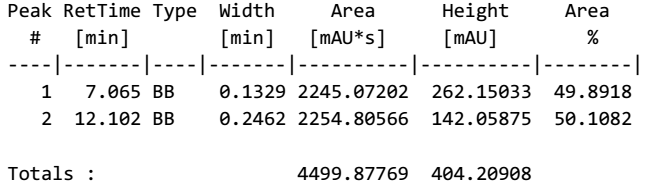


**HPLC (1R,2S)-1-phenyl-2-(trimethylsilyl)but-3-en-1-ol (2l, 93% ee)**


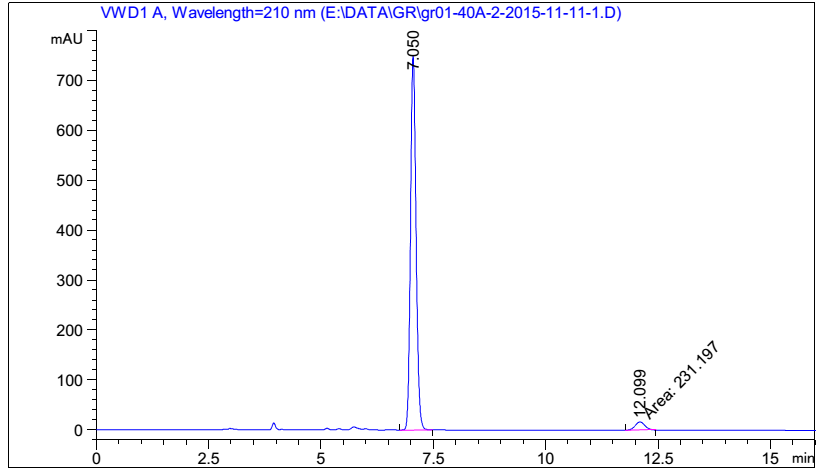


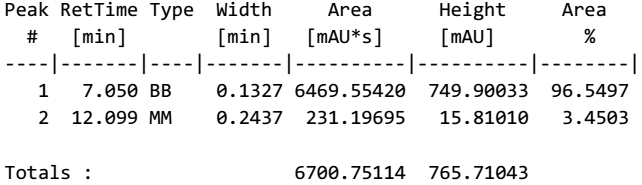


**^1^HNMR (400MHz,CDCl_3_) (1R,2S)-1-(4-bromophenyl)-2-(trimethylsilyl)but**

**-3-en-1-ol (2m)**


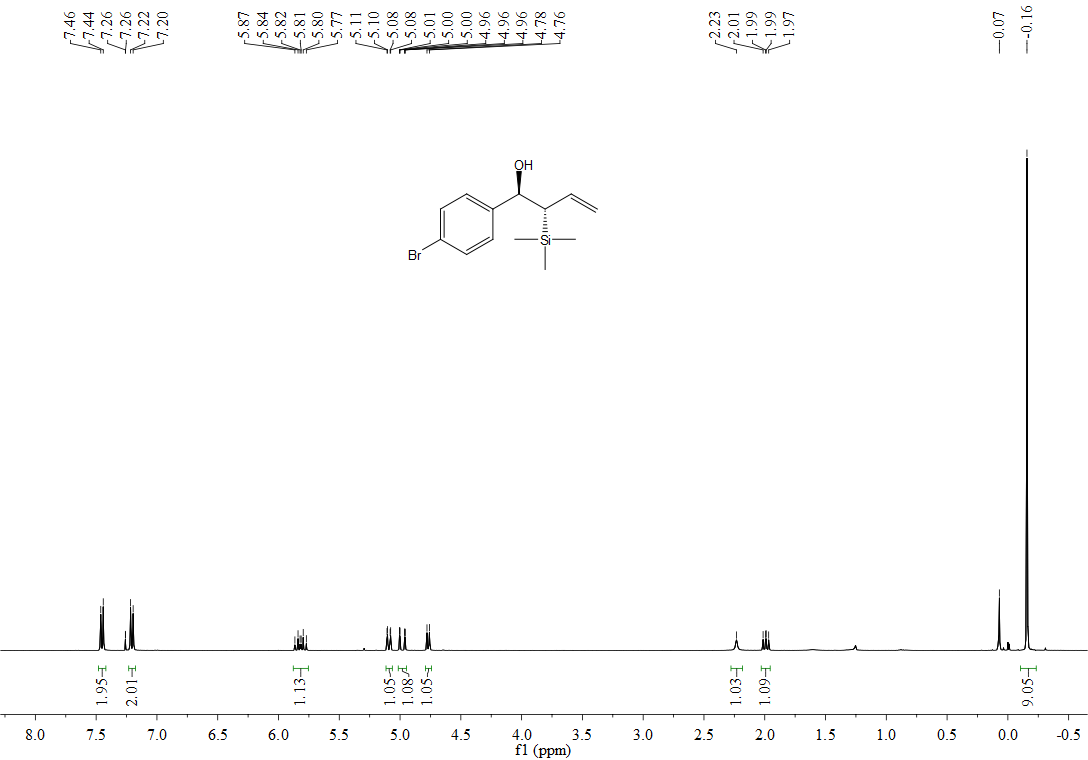


**^13^C NMR (101 MHz,CDCl_3_) (1R,2S)-1-(4-bromophenyl)-2-(trimethylsilyl)but**

**-3-en-1-ol (2m)**


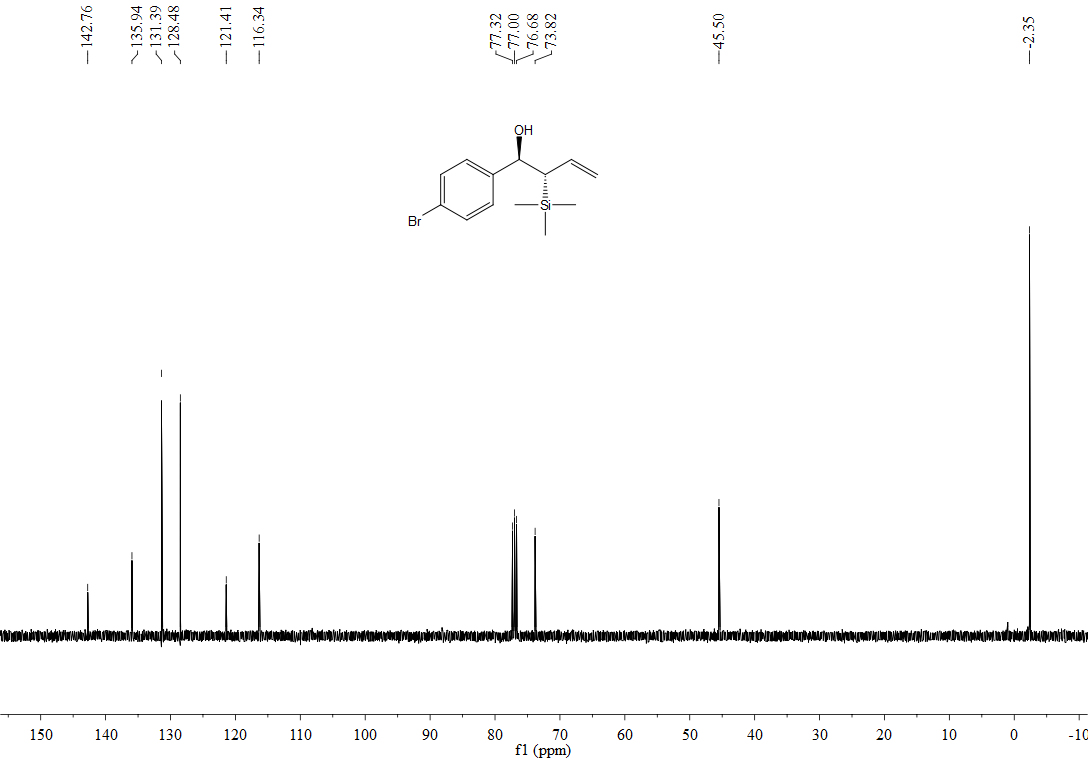


**HPLC (1R,2S)-1-(4-bromophenyl)-2-(trimethylsilyl)but-3-en-1-ol (2m, Racemic)**


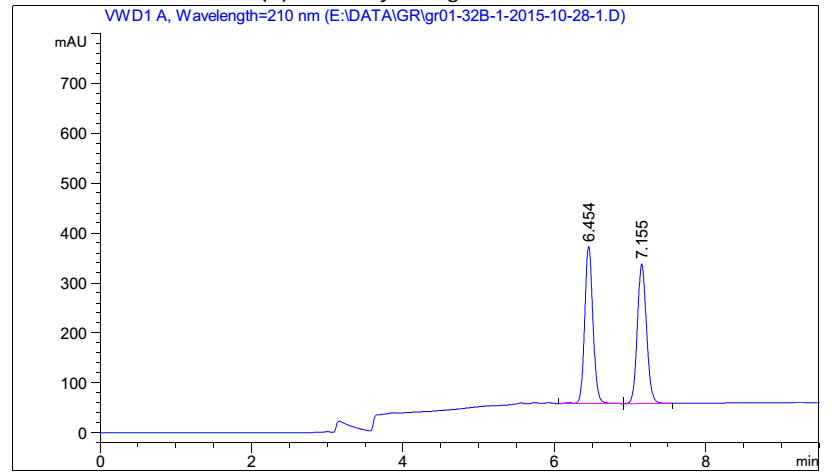


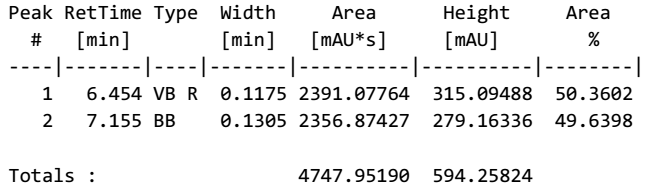


**HPLC (1R,2S)-1-(4-bromophenyl)-2-(trimethylsilyl)but-3-en-1-ol (2m, 92% ee)**


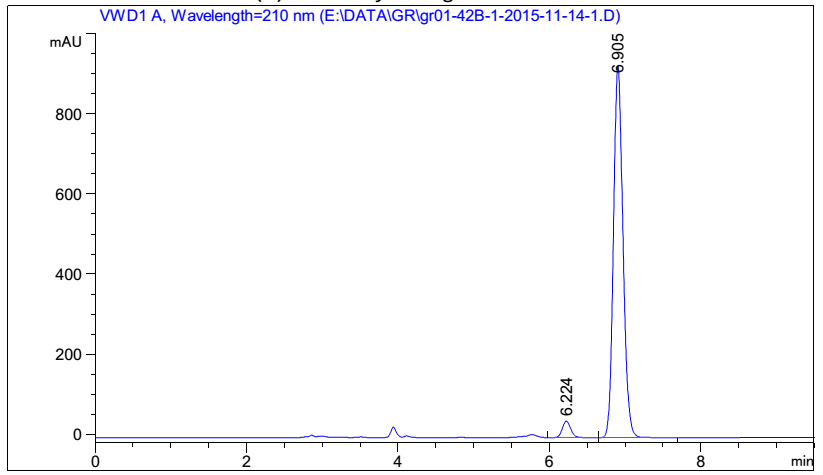


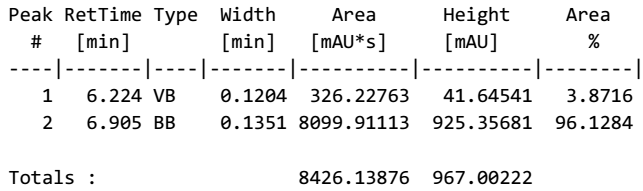


**^1^HNMR (400MHz,CDCl_3_)(1R,2S)-1-(2-fluorophenyl)-2-(trimethylsilyl)but-3-en-1-ol(2n)**


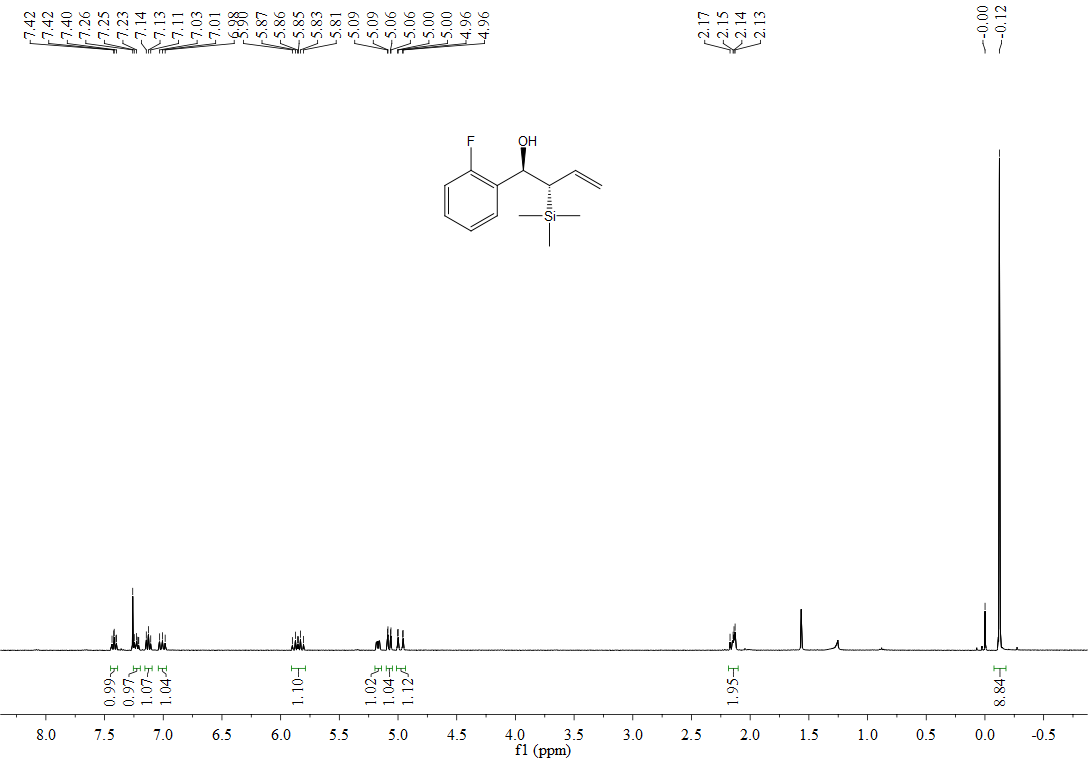


**^13^CNMR(101MHz,CDCl_3_)(1R,2S)-1-(2-fluorophenyl)-2-(trimethylsilyl)but-3-en-1-ol(2n)**


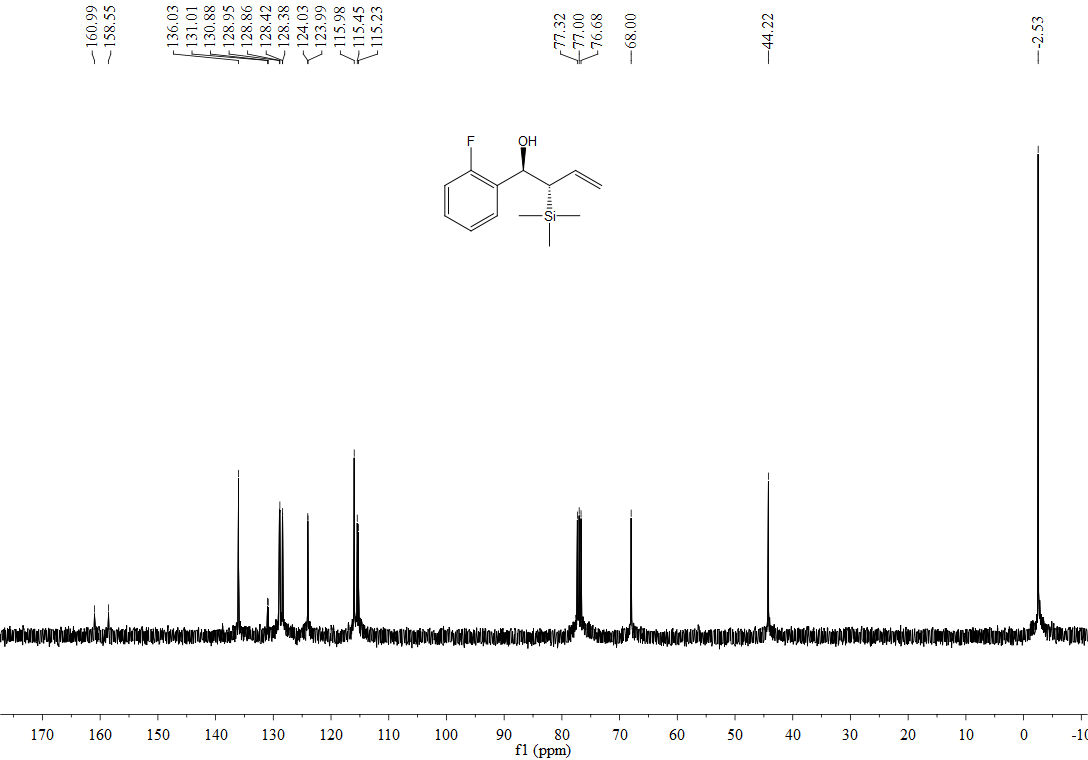


**HPLC (1R,2S)-1-(2-fluorophenyl)-2-(trimethylsilyl)but-3-en-1-ol (2n, Racemic)**


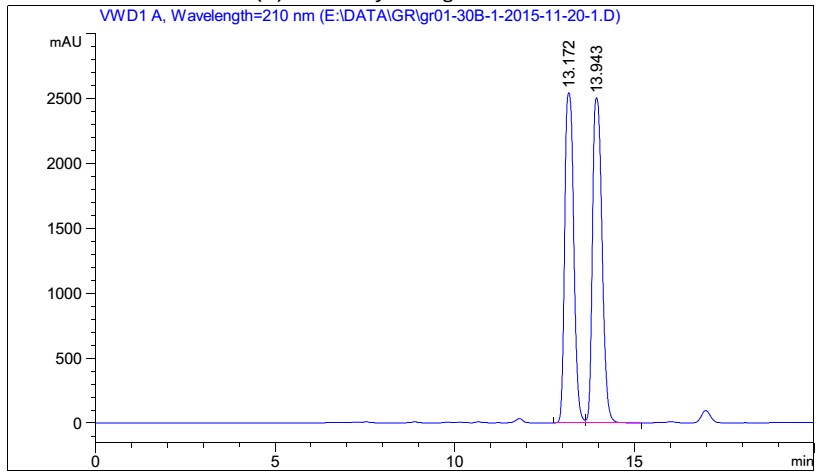


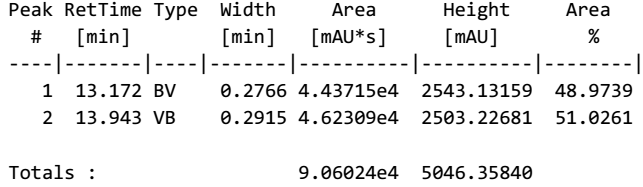


**HPLC (1R,2S)-1-(2-fluorophenyl)-2-(trimethylsilyl)but-3-en-1-ol (2n, 90% ee)**


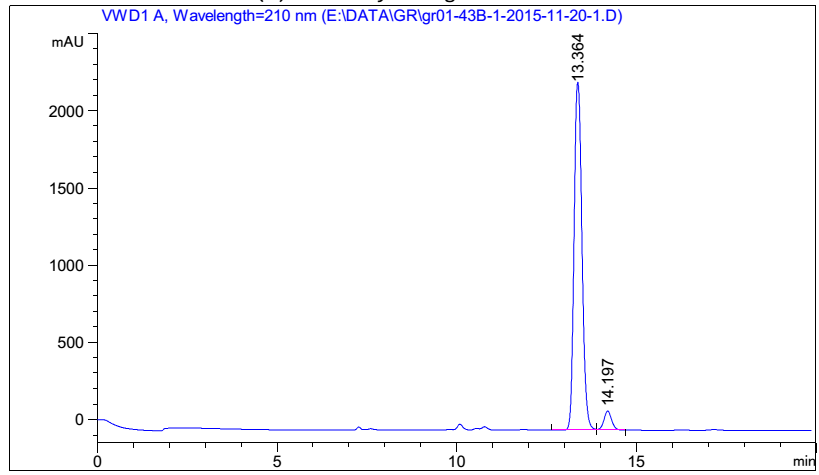


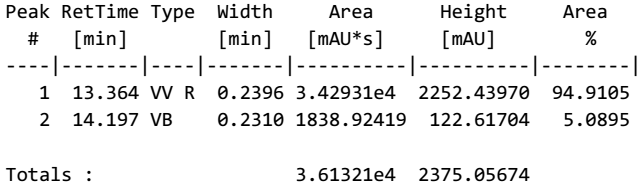


**^1^HNMR (400MHz,CDCl_3_)(1R,2S)-1-(3-fluorophenyl)-2-(trimethylsilyl)but-3-en-1-ol(2o)**

**
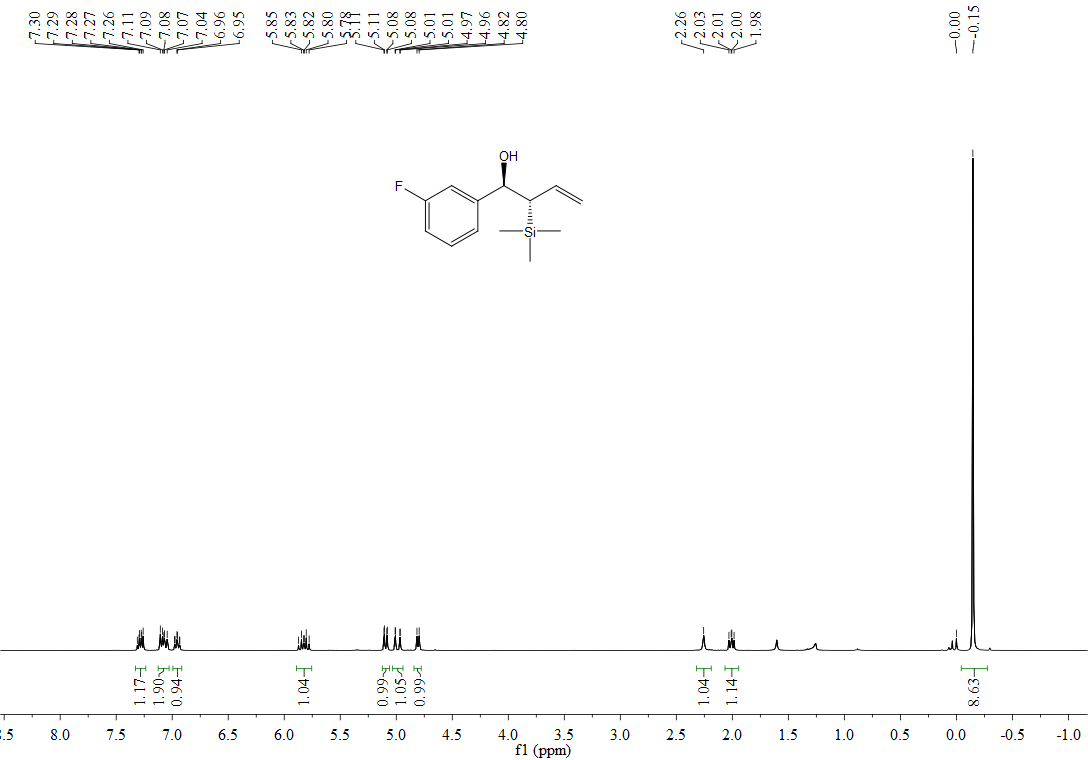
**

**^13^CNMR(101MHz,CDCl_3_)(1R,2S)-1-(3-fluorophenyl)-2-(trimethylsilyl)but-3-en-1-ol(2o)**


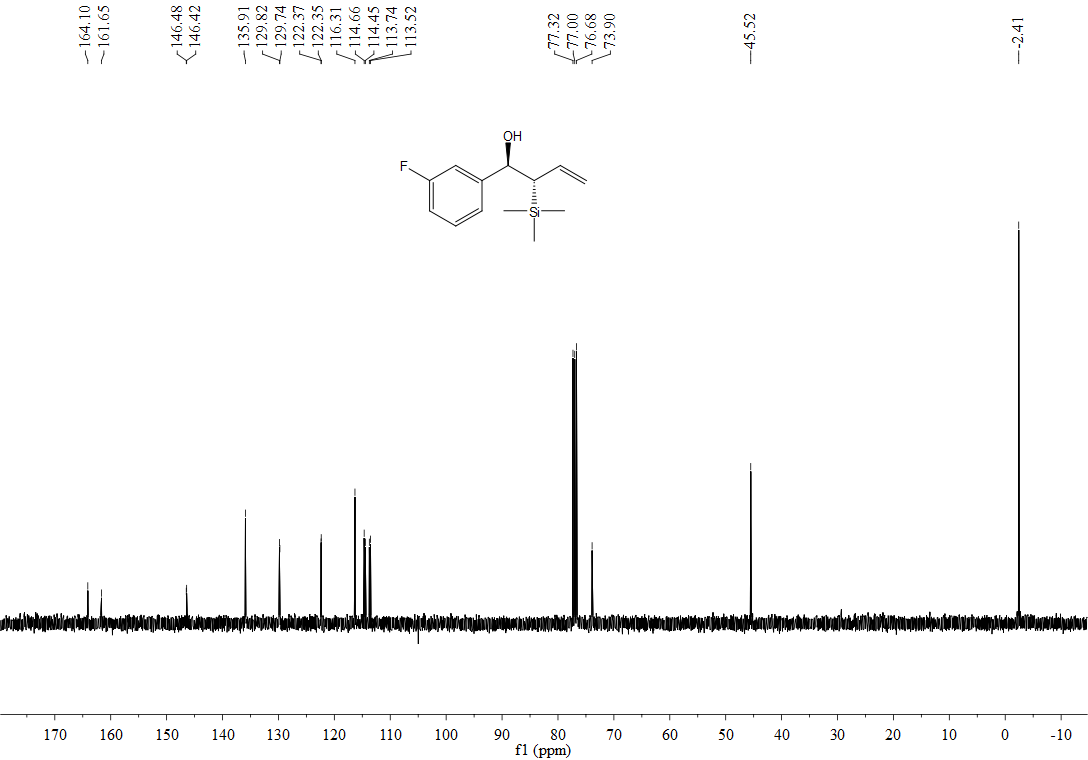


**HPLC (1R,2S)-1-(3-fluorophenyl)-2-(trimethylsilyl)but-3-en-1-ol (2o, Racemic)**


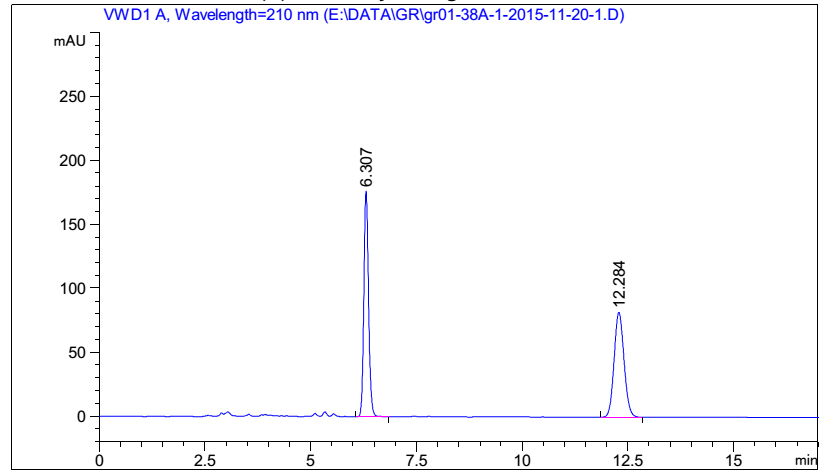


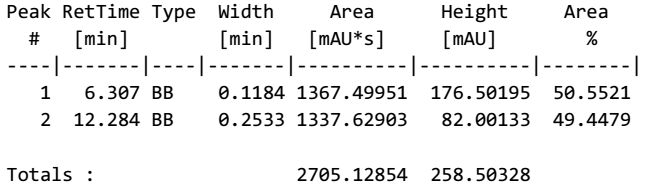


**HPLC (1R,2S)-1-(3-fluorophenyl)-2-(trimethylsilyl)but-3-en-1-ol (2o, 92% ee)**


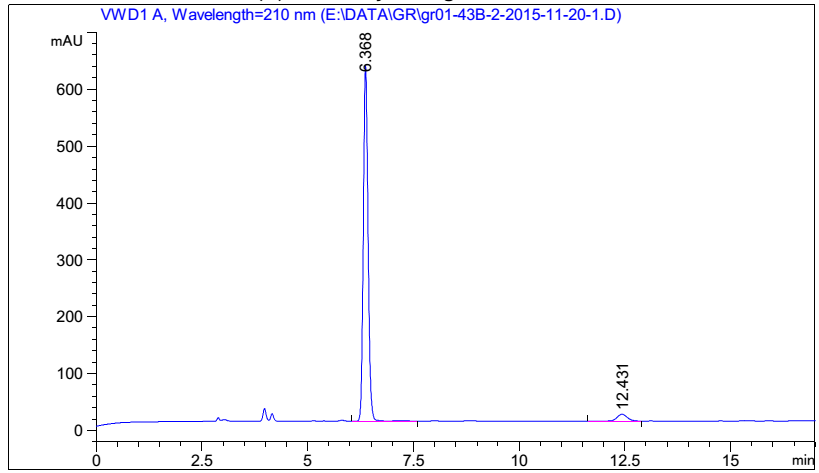


**^1^H NMR (400MHz,CDCl_3_) (1R,2S)-1-(4-chlorophenyl)-2-(trimethylsilyl)but-3-en-1-ol**

**(2p)**

**^13^C NMR (101MHz,CDCl_3_) (1R,2S)-1-(4-chlorophenyl)-2-(trimethylsilyl)but-3-en-1-ol**

**(2p)**

**HPLC (1R,2S)-1-(4-chlorophenyl)-2-(trimethylsilyl)but-3-en-1-ol (2p, Racemic)**

**HPLC (1R,2S)-1-(4-chlorophenyl)-2-(trimethylsilyl)but-3-en-1-ol (2p, 91% ee)**

**^1^H NMR (400MHz,CDCl_3_) (1R,2S)-1-(3-(trifluoromethyl)phenyl)-2-(trimethylsilyl)**

**but-3-en-1-ol (2q)**

**^13^C NMR (101MHz,CDCl_3_) (1R,2S)-1-(3-(trifluoromethyl)phenyl)-2-(trimethylsilyl)**

**but-3-en-1-ol (2q)**

**HPLC (1R,2S)-1-(3-(trifluoromethyl)phenyl)-2-(trimethylsilyl)but-3-en-1-ol**

**(2q, Racemic)**

**HPLC (1R,2S)-1-(3-(trifluoromethyl)phenyl)-2-(trimethylsilyl)but-3-en-1-ol**

**(2q, 91% ee)**

**^1^H NMR (400MHz,CDCl_3_) (1R,2S)-1-(m-tolyl)-2-(trimethylsilyl)but-3-en-1-ol (2r)**

**^13^C NMR (101MHz,CDCl_3_) (1R,2S)-1-(m-tolyl)-2-(trimethylsilyl)but-3-en-1-ol (2r)**

**HPLC (1R,2S)-1-(m-tolyl)-2-(trimethylsilyl)but-3-en-1-ol (2r, Racemic)**

**HPLC (1R,2S)-1-(m-tolyl)-2-(trimethylsilyl)but-3-en-1-ol (2r, 92% ee)**

**^1^H NMR (400MHz,CDCl_3_) (1R,2S)-1-(p-tolyl)-2-(trimethylsilyl)but-3-en-1-ol (2s)**

**^13^C NMR (101MHz,CDCl_3_) (1R,2S)-1-(p-tolyl)-2-(trimethylsilyl)but-3-en-1-ol (2s)**

**HPLC (1R,2S)-1-(p-tolyl)-2-(trimethylsilyl)but-3-en-1-ol (2s, Racemic)**

**HPLC (1R,2S)-1-(p-tolyl)-2-(trimethylsilyl)but-3-en-1-ol (2s, 91% ee)**

**^1^HNMR (400MHz,CDCl_3_) (1S,2S)-1-(thiophen-2-yl)-2-(trimethylsilyl)but-3-en-1-ol (2t)**

**^13^CNMR(101MHz,CDCl_3_) (1S,2S)-1-(thiophen-2-yl)-2-(trimethylsilyl)but-3-en-1-ol (2t)**

**HPLC (1R,2S)-1-(3-fluorophenyl)-2-(trimethylsilyl)but-3-en-1-ol (2t, Racemic)**

**HPLC (1R,2S)-1-(3-fluorophenyl)-2-(trimethylsilyl)but-3-en-1-ol (2t, 92% ee)**

**^1^HNMR (400MHz,CDCl_3_) (3R,4S,E)-1-phenyl-4-(trimethylsilyl)hexa-1,5-dien-3-ol (2u)**

**^13^CNMR(101MHz,CDCl_3_) (3R,4S,E)-1-phenyl-4-(trimethylsilyl)hexa-1,5-dien-3-ol (2u)**

**HPLC (3R,4S,E)-1-phenyl-4-(trimethylsilyl)hexa-1,5-dien-3-ol (2u, Racemic)**

**HPLC (3R,4S,E)-1-phenyl-4-(trimethylsilyl)hexa-1,5-dien-3-ol (2u, 98% ee)**

**^1^HNMR (400MHz,CDCl_3_) (3R,4S,E)-1-(4-fluorophenyl)-4-(trimethylsilyl)hexa**

**-1,5-dien-3-ol (2v)**

**^13^C NMR (101 MHz, CDCl_3_) (3R,4S,E)-1-(4-fluorophenyl)-4-(trimethylsilyl)hexa**

**-1,5-dien-3-ol (2v)**

**HPLC (3R,4S,E)-1-(4-fluorophenyl)-4-(trimethylsilyl)hexa-1,5-dien-3-ol (2v, Racemic)**

**HPLC (3R,4S,E)-1-(4-fluorophenyl)-4-(trimethylsilyl)hexa-1,5-dien-3-ol (2v, 97% ee)**

**^1^H NMR (400 MHz, CDCl_3_) (3R,4S)-1-phenyl-4-(trifluoromethyl)hex-5-en-3-ol (3a)**

**^19^F NMR (376 MHz, CDCl_3_) (3R,4S)-1-phenyl-4-(trifluoromethyl)hex-5-en-3-ol (3a)**

**^13^C NMR (100 MHz, CDCl_3_) (3R,4S)-1-phenyl-4-(trifluoromethyl)hex-5-en-3-ol (3a)**

**HPLC (3R,4S)-1-phenyl-4-(trifluoromethyl)hex-5-en-3-ol (3a, Racemic)**

**HPLC (3R,4S)-1-phenyl-4-(trifluoromethyl)hex-5-en-3-ol (3a, 91% ee)**

**^1^H NMR (400 MHz, CDCl_3_) (3S,4R)-3-(trifluoromethyl)non-1-en-4-ol (3b)**

**^19^F NMR (376 MHz, CDCl_3_) (3S,4R)-3-(trifluoromethyl)non-1-en-4-ol (3b)**

**^13^C NMR (100 MHz, CDCl_3_) (3S,4R)-3-(trifluoromethyl)non-1-en-4-ol (3b)**

**HPLC 3,5-nitrobenzoatederivative of (3S,4R)-3-(trifluoromethyl)non-1-en-4-ol (3b, Racemic)**

**HPLC 3,5-nitrobenzoatederivative of (3S,4R)-3-(trifluoromethyl)non-1-en-4-ol (3b, 95% ee)**

**^1^H NMR (400 MHz, CDCl_3_) (3S,4R)-8-chloro-3-(trifluoromethyl)oct-1-en-4-ol (3c)**

**^19^F NMR (376 MHz, CDCl_3_) (3S,4R)-8-chloro-3-(trifluoromethyl)oct-1-en-4-ol (3c)**

**^13^C NMR (100 MHz, CDCl_3_) (3S,4R)-8-chloro-3-(trifluoromethyl)oct-1-en-4-ol (3c)**

**HPLC 3,5-nitrobenzoatederivative of (3S,4R)-8-chloro-3-(trifluoromethyl)oct-**

**1-en-4-ol (3c, Racemic)**

**HPLC 3,5-nitrobenzoatederivative of (3S,4R)-8-chloro-3-(trifluoromethyl)oct-**

**1-en-4-ol (3c, 95% ee)**

**^1^H NMR (400 MHz, CDCl_3_) (3S,4R)-6,8,8-trimethyl-3-(trifluoromethyl)non-1-en-4-ol (3d)**

**^19^F NMR (376 MHz, CDCl_3_) (3S,4R)-6,8,8-trimethyl-3-(trifluoromethyl)non-1-en-4-ol (3d)**

**^13^C NMR (100 MHz, CDCl_3_) (3S,4R)-6,8,8-trimethyl-3-(trifluoromethyl)non-1-en-4-ol (3d)**

**HPLC 3,5-nitrobenzoatederivative of (3S,4R)-6,8,8-trimethyl-3-(trifluoromethyl)non**

**-1-en-4-ol (3d, racemic)**

**HPLC 3,5-nitrobenzoatederivative of (3S,4R)-6,8,8-trimethyl-3-(trifluoromethyl)non**

**-1-en-4-ol (3d, 98% de)**

**^1^H NMR (400 MHz, CDCl_3_) (2R,3S)-1-(2,2-dimethyl-1,3-dioxolan-4-yl)-3-(trifluoromethyl)pent-4-en-2-ol (3e)**

**^19^F NMR (376 MHz, CDCl_3_) (2R,3S)-1-(2,2-dimethyl-1,3-dioxolan-4-yl)-3-(trifluoromethyl)pent-4-en-2-ol (3e)**

**^13^C NMR (100 MHz, CDCl_3_) (2R,3S)-1-(2,2-dimethyl-1,3-dioxolan-4-yl)-3-(trifluoromethyl)pent-4-en-2-ol (3e)**

**HPLC 3,5-nitrobenzoatederivative of (2R,3S)-1-(2,2-dimethyl-1,3-dioxolan-4-yl)**

**-3-(trifluoromethyl)pent-4-en-2-ol (3e, racemic)**

**HPLC 3,5-nitrobenzoatederivative of (2R,3S)-1-(2,2-dimethyl-1,3-dioxolan-4-yl)**

**-3-(trifluoromethyl)pent-4-en-2-ol (3e, 98% de)**

**^1^H NMR (400 MHz, CDCl_3_) (3S,4R,6R)-6,10-dimethyl-3-(trifluoromethyl)undeca-1,9-dien-4-ol (3f)**

**^19^F NMR (376 MHz, CDCl_3_) (3S,4R,6R)-6,10-dimethyl-3-(trifluoromethyl)undeca-1,9-dien-4-ol (3f)**

**^13^C NMR (100 MHz, CDCl_3_) (3S,4R,6R)-6,10-dimethyl-3-(trifluoromethyl)undeca-1,9-dien-4-ol (3f)**

**HPLC 3,5-nitrobenzoatederivative of (3S,4R,6R)-6,10-dimethyl-3-(trifluoromethyl)**

**undeca-1,9-dien-4-ol (3f, racemic)**

**HPLC 3,5-nitrobenzoatederivative of (3S,4R,6R)-6,10-dimethyl-3-(trifluoromethyl)**

**undeca-1,9-dien-4-ol (3f, 96% ee)**

**^1^H NMR (400 MHz, CDCl_3_) (2S,3S)-1-(benzyloxy)-3-(trifluoromethyl)pent-4-en-2-ol (3g)**

**^19^FNMR (376 MHz, CDCl_3_) (2S,3S)-1-(benzyloxy)-3-(trifluoromethyl)pent-4-en-2-ol (3g)**

**^13^C NMR (100 MHz, CDCl_3_)(2S,3S)-1-(benzyloxy)-3-(trifluoromethyl)pent-4-en-2-ol (3g)**

**HPLC (2S,3S)-1-(benzyloxy)-3-(trifluoromethyl)pent-4-en-2-ol (3g, racemic)**

**HPLC (2S,3S)-1-(benzyloxy)-3-(trifluoromethyl)pent-4-en-2-ol (3g, 90% ee)**

**^1^H NMR (400 MHz, CDCl_3_) (3R,4S,E)-1-phenyl-4-(trifluoromethyl)hexa-1,5-dien-3-ol (3h)**

**^19^F NMR (376 MHz, CDCl_3_) (3R,4S,E)-1-phenyl-4-(trifluoromethyl)hexa-1,5-dien-3-ol (3h)**

**^13^C NMR (100 MHz, CDCl_3_)(3R,4S,E)-1-phenyl-4-(trifluoromethyl)hexa-1,5-dien-3-ol (3h)**

**HPLC (3R,4S,E)-1-phenyl-4-(trifluoromethyl)hexa-1,5-dien-3-ol (3h, Racemic)**

**HPLC (3R,4S,E)-1-phenyl-4-(trifluoromethyl)hexa-1,5-dien-3-ol (3h, 93% ee)**

**^1^H NMR (400 MHz, CDCl_3_) (3R,4S,E)-1-(4-methoxyphenyl)-4-(trifluoromethyl)hexa-1,5-dien-3-ol (3i)**

**^19^F NMR (376 MHz, CDCl_3_) (3R,4S,E)-1-(4-methoxyphenyl)-4-(trifluoromethyl)hexa-1,5-dien-3-ol (3i)**

**^13^C NMR (100 MHz, CDCl_3_) (3R,4S,E)-1-(4-methoxyphenyl)-4-(trifluoromethyl)hexa-1,5-dien-3-ol (3i)**

**HPLC (3R,4S,E)-1-(4-methoxyphenyl)-4-(trifluoromethyl)hexa-1,5-dien-3-ol (3i, racemic)**

**HPLC (3R,4S,E)-1-(4-methoxyphenyl)-4-(trifluoromethyl)hexa-1,5-dien-3-ol (3i, 95% ee)**

**^1^H NMR (400 MHz, CDCl_3_) (3R,4S,E)-1-(4-fluorophenyl)-4-(trifluoromethyl)hexa-1,5-dien-3-ol (3j)**

**^19^F NMR (376 MHz, CDCl_3_) (3R,4S,E)-1-(4-fluorophenyl)-4-(trifluoromethyl)hexa-1,5-dien-3-ol (3j)**

**^13^C NMR (100 MHz, CDCl_3_) (3R,4S,E)-1-(4-fluorophenyl)-4-(trifluoromethyl)hexa-1,5-dien-3-ol (3j)**

**HPLC (3R,4S,E)-1-(4-fluorophenyl)-4-(trifluoromethyl)hexa-1,5-dien-3-ol(3j, racemic)**

**HPLC (3R,4S,E)-1-(4-fluorophenyl)-4-(trifluoromethyl)hexa-1,5-dien-3-ol(3j,93% ee)**

**^1^H NMR (400 MHz, CDCl_3_) (3R,4S,E)-1-(2-methoxyphenyl)-4-(trifluoromethyl)hexa-1,5-dien-3-ol (3k)**

**^19^F NMR (376 MHz, CDCl_3_) (3R,4S,E)-1-(2-methoxyphenyl)-4-(trifluoromethyl)hexa-1,5-dien-3-ol (3k)**

**^13^C NMR (100 MHz, CDCl_3_) (3R,4S,E)-1-(2-methoxyphenyl)-4-(trifluoromethyl)hexa-1,5-dien-3-ol (3k)**

**HPLC (3R,4S,E)-1-(2-methoxyphenyl)-4-(trifluoromethyl)hexa-1,5-dien-3-ol (3k, racemic)**

**HPLC (3R,4S,E)-1-(2-methoxyphenyl)-4-(trifluoromethyl)hexa-1,5-dien-3-ol (3k, 93% ee)**

**^1^H NMR (400 MHz, CDCl_3_)(3S,4R)-3-(difluoromethyl)non-1-en-4-ol (3l)**

**^19^FNMR (376 MHz, CDCl_3_) (3S,4R)-3-(difluoromethyl)non-1-en-4-ol (3l)**

**^13^C NMR (100 MHz, CDCl_3_) (3S,4R)-3-(difluoromethyl)non-1-en-4-ol (3l)**

**HPLC 3,5-nitrobenzoatederivative of (3S,4R)-3-(difluoromethyl)non-1-en-4-ol (3l, racemic)**

**HPLC 3,5-nitrobenzoatederivative of (3S,4R)-3-(difluoromethyl)non-1-en-4-ol (3l, 96% ee)**

**^1^H NMR (400 MHz, CDCl_3_) (3S,4R)-8-chloro-3-(difluoromethyl)oct-1-en-4-ol (3m)**

**^19^F NMR (376 MHz, CDCl_3_) (3S,4R)-8-chloro-3-(difluoromethyl)oct-1-en-4-ol (3m)**

**^13^C NMR (100 MHz, CDCl_3_) (3S,4R)-8-chloro-3-(difluoromethyl)oct-1-en-4-ol (3m)**

**HPLC 3,5-nitrobenzoatederivative of(3S,4R)-8-chloro-3-(difluoromethyl)oct**

**-1-en-4-ol (3m, racemic)**

**HPLC 3,5-nitrobenzoatederivative of(3S,4R)-8-chloro-3-(difluoromethyl)oct**

**-1-en-4-ol (3m, 96% ee)**

**^1^H NMR (400 MHz, CDCl_3_) (3R,4S)-4-(difluoromethyl)-1-phenylhex-5-en-3-ol (3n)**

**^19^FNMR (376 MHz, CDCl_3_) (3R,4S)-4-(difluoromethyl)-1-phenylhex-5-en-3-ol (3n)**

**^13^C NMR (100 MHz, CDCl_3_)(3R,4S)-4-(difluoromethyl)-1-phenylhex-5-en-3-ol (3n)**

**HPLC (3R,4S)-4-(difluoromethyl)-1-phenylhex-5-en-3-ol (3n, racemic)**

**HPLC (3R,4S)-4-(difluoromethyl)-1-phenylhex-5-en-3-ol (3n, 92% ee)**

**^1^H NMR (400 MHz, CDCl_3_) (3S,4R,6R)-3-(difluoromethyl)-6,10-dimethylundeca-1,9-dien-4-ol (3o)**

**^19^F NMR (376 MHz, CDCl_3_) (3S,4R,6R)-3-(difluoromethyl)-6,10-dimethylundeca-1,9-dien-4-ol (3o)**

**^13^C NMR (100 MHz, CDCl_3_)(3S,4R,6R)-3-(difluoromethyl)-6,10-dimethylundeca-1,9-dien-4-ol (3o)**

**HPLC 3,5-nitrobenzoatederivative of (3S,4R,6R)-3-(difluoromethyl)-6,10-**

**Dimethylundeca-1,9-dien-4-ol (3o, racemic)**

**HPLC 3,5-nitrobenzoatederivative of (3S,4R,6R)-3-(difluoromethyl)-6,10-**

**Dimethylundeca-1,9-dien-4-ol(3o, 98% ee)**

**^1^H NMR (400 MHz, CDCl_3_)(3R,4S,E)-4-(fluoromethyl)-1-phenylhexa-1,5-dien-3-ol (3p)**

**^19^F NMR (376 MHz, CDCl_3_) (3R,4S,E)-4-(fluoromethyl)-1-phenylhexa-1,5-dien-3-ol (3p)**

**^13^C NMR (100 MHz, CDCl_3_) (3R,4S,E)-4-(fluoromethyl)-1-phenylhexa-1,5-dien-3-ol (3p)**

**HPLC (3R,4S,E)-4-(fluoromethyl)-1-phenylhexa-1,5-dien-3-ol (3p, racemic)**

**HPLC (3R,4S,E)-4-(fluoromethyl)-1-phenylhexa-1,5-dien-3-ol (3p, 94% ee)**

**^1^H NMR (400 MHz,CDCl_3_) (3R,4S,E)-4-(fluoromethyl)-1-(4-fluorophenyl)hexa-1,5-dien-3-ol (3q)**

**^19^F NMR (376 MHz, CDCl_3_) (3R,4S,E)-4-(fluoromethyl)-1-(4-fluorophenyl)hexa-1,5-dien-3-ol (3q)**

**^13^C NMR (100 MHz, CDCl_3_) (3R,4S,E)-4-(fluoromethyl)-1-(4-fluorophenyl)hexa-1,5-dien-3-ol (3q)**

**HPLC (3R,4S,E)-4-(fluoromethyl)-1-(4-fluorophenyl)hexa-1,5-dien-3-ol (3q, racemic)**

**HPLC (3R,4S,E)-4-(fluoromethyl)-1-(4-fluorophenyl)hexa-1,5-dien-3-ol (3q, 96% ee)**

**^1^H NMR (400 MHz, CDCl_3_) (3R,4S)-1-phenyl-4-(phenylthio)hex-5-en-3-ol (4a)**

**^13^C NMR (101 MHz, CDCl_3_) (3R,4S)-1-phenyl-4-(phenylthio)hex-5-en-3-ol (4a)**

**HPLC (3R,4S)-1-phenyl-4-(phenylthio)hex-5-en-3-ol (4a, Racemic)**

**HPLC (3R,4S)-1-phenyl-4-(phenylthio)hex-5-en-3-ol (4a, 91% ee)**

**^1^H NMR (400 MHz, CDCl_3_) (3S,4R)-3-(phenylthio)non-1-en-4-ol (4b)**

**^13^C NMR (101 MHz, CDCl_3_) (3S,4R)-3-(phenylthio)non-1-en-4-ol (4b)**

**HPLC (3S,4R)-3-(phenylthio)non-1-en-4-ol (4b, Racemic)**

**HPLC (3S,4R)-3-(phenylthio)non-1-en-4-ol (4b, 93% ee)**

**^1^H NMR (400 MHz, CDCl_3_) (3S,4R)-8-chloro-3-(phenylthio)oct-1-en-4-ol (4c)**

**^13^C NMR (101 MHz, CDCl_3_) (3S,4R)-8-chloro-3-(phenylthio)oct-1-en-4-ol (4c)**

**HPLC (3S,4R)-8-chloro-3-(phenylthio)oct-1-en-4-ol (4c, Racemic)**

**HPLC (3S,4R)-8-chloro-3-(phenylthio)oct-1-en-4-ol (4c, 93% ee)**

**^1^H NMR (400 MHz, CDCl_3_) (3S,4R)-3-(phenylthio)octa-1,7-dien-4-ol (4d)**

**^13^C NMR (101 MHz, CDCl_3_) (3S,4R)-3-(phenylthio)octa-1,7-dien-4-ol (4d)**

**HPLC (3S,4R)-3-(phenylthio)octa-1,7-dien-4-ol (4d, Racemic)**

**HPLC (3S,4R)-3-(phenylthio)octa-1,7-dien-4-ol (4d, 92% ee)**

**^1^H NMR (400 MHz, CDCl_3_) (2R,3S)-1-(benzyloxy)-3-(phenylthio)pent-4-en-2-ol (4e)**

**^13^C NMR (101 MHz, CDCl_3_) (2R,3S)-1-(benzyloxy)-3-(phenylthio)pent-4-en-2-ol (4e)**

**HPLC (2R,3S)-1-(benzyloxy)-3-(phenylthio)pent-4-en-2-ol (4e, Racemic)**

**HPLC (2R,3S)-1-(benzyloxy)-3-(phenylthio)pent-4-en-2-ol (4e, 90% ee)**

**^1^H NMR (400 MHz, CDCl_3_)**

**2-((3R,4S)-3-hydroxy-4-(phenylthio)hex-5-en-1-yl)isoindoline-1,3-dione (4f)**

**^13^C NMR (101 MHz, CDCl_3_)**

**2-((3R,4S)-3-hydroxy-4-(phenylthio)hex-5-en-1-yl)isoindoline-1,3-dione (4f)**

**HPLC**

**2-((3R,4S)-3-hydroxy-4-(phenylthio)hex-5-en-1-yl)isoindoline-1,3-dione (4f, Racemic)**

**HPLC**

**2-((3R,4S)-3-hydroxy-4-(phenylthio)hex-5-en-1-yl)isoindoline-1,3-dione (4f, 91% ee)**

**^1^H NMR (400 MHz, CDCl_3_) (3R,4S)-1-(methylthio)-4-(phenylthio)hex-5-en-3-ol (4g)**

**^13^C NMR (101 MHz, CDCl_3_) (3R,4S)-1-(methylthio)-4-(phenylthio)hex-5-en-3-ol (4g)**

**HPLC (3R,4S)-1-(methylthio)-4-(phenylthio)hex-5-en-3-ol (4g, Racemic)**

**HPLC (3R,4S)-1-(methylthio)-4-(phenylthio)hex-5-en-3-ol (4g, 91% ee)**

**^1^H NMR (400 MHz, CDCl_3_) (3S,4R,E)-3-(phenylthio)nona-1,5-dien-4-ol (4h)**

**^13^C NMR (101 MHz, CDCl_3_) (3S,4R,E)-3-(phenylthio)nona-1,5-dien-4-ol (4h)**

**HPLC (3S,4R,E)-3-(phenylthio)nona-1,5-dien-4-ol (4h, Racemic)**

**HPLC (3S,4R,E)-3-(phenylthio)nona-1,5-dien-4-ol (4h, 90% ee)**

**^1^H NMR (400 MHz, CDCl_3_) (3R,4S,E)-1-phenyl-4-(phenylthio)hexa-1,5-dien-3-ol (4i)**

**^13^C NMR (101 MHz, CDCl_3_) (3R,4S,E)-1-phenyl-4-(phenylthio)hexa-1,5-dien-3-ol (4i)**

**HPLC (3R,4S,E)-1-phenyl-4-(phenylthio)hexa-1,5-dien-3-ol (4i, Racemic)**

**HPLC (3R,4S,E)-1-phenyl-4-(phenylthio)hexa-1,5-dien-3-ol (4i, 95% ee)**

**^1^H NMR (400 MHz, CDCl_3_)**

**(3R,4S,E)-1-(4-chlorophenyl)-4-(phenylthio)hexa-1,5-dien-3-ol (4j)**

**^13^C NMR (101 MHz, CDCl_3_)**

**(3R,4S,E)-1-(4-chlorophenyl)-4-(phenylthio)hexa-1,5-dien-3-ol (4j)**

**HPLC (3R,4S,E)-1-(4-chlorophenyl)-4-(phenylthio)hexa-1,5-dien-3-ol (4j, Racemic)**

**HPLC (3R,4S,E)-1-(4-chlorophenyl)-4-(phenylthio)hexa-1,5-dien-3-ol (4j, 93% ee)**

**^1^H NMR (400 MHz, CDCl_3_) (3R,4S,E)-1-(4-bromophenyl)-4-(phenylthio)hexa-1,5-dien-3-ol (4k)**

**^13^C NMR (101 MHz, CDCl_3_) (3R,4S,E)-1-(4-bromophenyl)-4-(phenylthio)hexa-1,5-dien-3-ol (4k)**

**HPLC (3R,4S,E)-1-(4-bromophenyl)-4-(phenylthio)hexa-1,5-dien-3-ol (4k, Racemic)**

**HPLC (3R,4S,E)-1-(4-bromophenyl)-4-(phenylthio)hexa-1,5-dien-3-ol (4k, 93% ee)**

**^1^H NMR (400 MHz, CDCl_3_)**

**(3R,4S,E)-1-(3-fluorophenyl)-4-(phenylthio)hexa-1,5-dien-3-ol (4l)**

**^13^C NMR (101 MHz, CDCl_3_)**

**(3R,4S,E)-1-(3-fluorophenyl)-4-(phenylthio)hexa-1,5-dien-3-ol (4l)**

**HPLC (3R,4S,E)-1-(3-fluorophenyl)-4-(phenylthio)hexa-1,5-dien-3-ol (4l, Racemic)**

**HPLC (3R,4S,E)-1-(3-fluorophenyl)-4-(phenylthio)hexa-1,5-dien-3-ol (4l, 93% ee)**

**^1^H NMR (400 MHz, CDCl_3_) (3R,4S,E)-4-(phenylthio)-1-(o-tolyl)hexa-1,5-dien-3-ol (4m)**

**^13^C NMR (101 MHz, CDCl_3_) (3R,4S,E)-4-(phenylthio)-1-(o-tolyl)hexa-1,5-dien-3-ol (4m)**

**HPLC (3R,4S,E)-4-(phenylthio)-1-(o-tolyl)hexa-1,5-dien-3-ol (4m, Racemic)**

**HPLC (3R,4S,E)-4-(phenylthio)-1-(o-tolyl)hexa-1,5-dien-3-ol (4m, 93% ee)**

**^1^H NMR (400 MHz, CDCl_3_)**

**(3R,4S,E)-1-(2-methoxyphenyl)-4-(phenylthio)hexa-1,5-dien-3-ol (4n)**

**^13^C NMR (101 MHz, CDCl_3_)**

**(3R,4S,E)-1-(2-methoxyphenyl)-4-(phenylthio)hexa-1,5-dien-3-ol (4n)**

**HPLC (3R,4S,E)-1-(2-methoxyphenyl)-4-(phenylthio)hexa-1,5-dien-3-ol (4n, Racemic)**

**HPLC (3R,4S,E)-1-(2-methoxyphenyl)-4-(phenylthio)hexa-1,5-dien-3-ol (4n, 94% ee)**

**^1^H NMR (400MHz,CDCl_3_)**

**(5R,8R,9S,10S,13R,14S,17R)-17-((2R,5R,6S)-5-hydroxy-6-(trimethylsilyl)oct-7-en-2-yl)-10,13-dimethyltetradecahydro-1H-cyclopenta[a]phenanthren-3(2H)-one (5)**

**^13^C NMR (101MHz,CDCl_3_)**

**(5R,8R,9S,10S,13R,14S,17R)-17-((2R,5R,6S)-5-hydroxy-6-(trimethylsilyl)oct-7-en-2-yl)-10,13-dimethyltetradecahydro-1H-cyclopenta[a]phenanthren-3(2H)-one (5)**

**HPLC(5R,8R,9S,10S,13R,14S,17R)-17-((2R,5R,6S)-5-hydroxy-6-(trimethylsilyl)oct-7-en-2-yl)-10,13-dimethyltetradecahydro-1H-cyclopenta[a]phenanthren-3(2H)-one (5, Racemic)**

**HPLC(5R,8R,9S,10S,13R,14S,17R)-17-((2R,5R,6S)-5-hydroxy-6-(trimethylsilyl)oct-7-en-2-yl)-10,13-dimethyltetradecahydro-1H-cyclopenta[a]phenanthren-3(2H)-one (5, 99% de)**

**^1^H NMR (400 MHz, CDCl_3_) (5R,8R,9S,10S,13R,14S,17R)-17-((2R,5R,6S)-5-hydroxy-6-(trifluoromethyl)oct-7-en-2-yl)-10,13-dimethyltetradecahydro-1H-cyclopenta[a]phenanthren-3(2H)-one (6)**

**^19^F NMR (376 MHz, CDCl_3_) (5R,8R,9S,10S,13R,14S,17R)-17-((2R,5R,6S)-5-hydroxy-6-(trifluoromethyl)oct-7-en-2-yl)-10,13-dimethyltetradecahydro-1H-cyclopenta[a]phenanthren-3(2H)-one (6)**

**^13^C NMR (100 MHz, CDCl_3_)(5R,8R,9S,10S,13R,14S,17R)-17-((2R,5R,6S)-5-hydroxy-6-(trifluoromethyl)oct-7-en-2-yl)-10,13-dimethyltetradecahydro-1H-cyclopenta[a]phenanthren-3(2H)-one (6)**

**HPLC3,5-nitrobenzoatederivative of (5R,8R,9S,10S,13R,14S,17R)-17-((2R,5R,6S)-5-**

**hydroxy-6-(trifluoromethyl)oct-7-en-2-yl)-10,13-dimethyltetradecahydro-1H-**

**cyclopenta[a]phenanthren-3(2H)-one(6, racemic)**

**HPLC3,5-nitrobenzoatederivative of (5R,8R,9S,10S,13R,14S,17R)-17-((2R,5R,6S)-5-**

**hydroxy-6-(trifluoromethyl)oct-7-en-2-yl)-10,13-dimethyltetradecahydro-1H-**

**cyclopenta[a]phenanthren-3(2H)-one(6, 90% ee)**

**^1^H NMR (400 MHz, CDCl_3_)**

**(5R,8R,9S,10S,13R,14S,17R)-17-((2R,5R,6S)-5-hydroxy-6-(phenylthio)oct-7-en-2-yl)-10,13-dimethyltetradecahydro-1H-cyclopenta[a]phenanthren-3(2H)-one (7)**

**^13^C NMR (101 MHz, CDCl_3_)**

**(5R,8R,9S,10S,13R,14S,17R)-17-((2R,5R,6S)-5-hydroxy-6-(phenylthio)oct-7-en-2-yl)-10,13-dimethyltetradecahydro-1H-cyclopenta[a]phenanthren-3(2H)-one (7)**

**HPLC (5R,8R,9S,10S,13R,14S,17R)-17-((2R,5R,6S)-5-hydroxy-6-(phenylthio)oct-7-en-2-yl)-10,13-dimethyltetradecahydro-1H-cyclopenta[a]phenanthren-3(2H)-one (7, Racemic)**

**HPLC (5R,8R,9S,10S,13R,14S,17R)-17-((2R,5R,6S)-5-hydroxy-6-(phenylthio)oct-7-en-2-yl)-10,13-dimethyltetradecahydro-1H-cyclopenta[a]phenanthren-3(2H)-one (7, 93% ee)**

**^1^H NMR (400MHz,CDCl_3_) (S,E)-1-(4-bromophenyl)-4-fluorobut-2-en-1-ol (8)**

**^13^C NMR (101MHz,CDCl_3_) (S,E)-1-(4-bromophenyl)-4-fluorobut-2-en-1-ol (8)**

**HPLC (S,E)-1-(4-bromophenyl)-4-fluorobut-2-en-1-ol (8, Racemic)**

**HPLC (S,E)-1-(4-bromophenyl)-4-fluorobut-2-en-1-ol (8, 91% ee)**

**^1^H NMR (400MHz,CDCl_3_) (2S,6R)-2,6-diphenethyl-3,6-dihydro-2H-pyran (9)**

**^13^C NMR (101MHz,CDCl_3_) (2S,6R)-2,6-diphenethyl-3,6-dihydro-2H-pyran (9)**

**HPLC (2S,6R)-2,6-diphenethyl-3,6-dihydro-2H-pyran (9, Racemic)**

**HPLC (2S,6R)-2,6-diphenethyl-3,6-dihydro-2H-pyran (9, 94% ee)**
